# Association of Chemoradiotherapy With Outcomes Among Patients With Stage I to II vs Stage III Small Cell Lung Cancer: Secondary Analysis of a Randomized Clinical Trial

**DOI:** 10.1001/jamaoncol.2018.5335

**Published:** 2018-12-06

**Authors:** Ahmed Salem, Hitesh Mistry, Matthew Hatton, Imogen Locke, Isabelle Monnet, Fiona Blackhall, Corinne Faivre-Finn

**Affiliations:** 1 Division of Cancer Sciences, University of Manchester, Manchester, United Kingdom; 2 Division of Pharmacy, University of Manchester, Manchester, United Kingdom; 3 Weston Park Hospital, Sheffield, United Kingdom; 4 Royal Marsden Hospital, Surrey, United Kingdom; 5 Centre Hospitalier Intercommunal de Créteil, Créteil, France

## Abstract

**Importance:**

There is limited evidence to guide stage I to II small cell lung cancer (SCLC) treatment.

**Objective:**

To examine the characteristics and outcomes among patients with stage I to II SCLC treated with modern chemoradiotherapy.

**Design, Setting, and Participants:**

In this post hoc secondary analysis of the Concurrent Once-Daily vs Twice-Daily Radiotherapy Trial (CONVERT), a multicenter phase 3 trial conducted in patients with limited-stage SCLC from April 7, 2008, to November 29, 2013, patients with TNM stage I to II SCLC were compared with those with stage III disease. Data analysis was performed from November 1, 2017, to February 28, 2018.

**Interventions:**

In CONVERT, patients were randomized to receive twice-daily (45 Gy in 30 fractions) or once-daily (66 Gy in 33 fractions) chemoradiotherapy. Prophylactic cranial irradiation (PCI) was offered, if indicated.

**Main Outcomes and Measures:**

The primary trial end point was overall survival (OS). TNM staging information was collected prospectively; this was an unplanned analysis because stratification was not performed according to TNM stage.

**Results:**

A total of 509 (277 [54.4%] men; mean [SD] age, 61.5 [8.3] years) of 543 patients (93.7%) with TNM staging information were eligible for this subgroup analysis, and 86 of the 509 (16.9%) had TNM stage I to II disease. The median gross tumor volume was smaller in patients with stage I to II disease (38.4 cm^3^; range, 2.2-593.0 cm^3^) compared with patients with stage III disease (93 cm^3^; range, 0.5-513.4 cm^3^) (*P* < .001). No other significant differences were found in baseline and treatment characteristics and chemoradiotherapy adherence between the 2 groups or the number of patients with stage I to II disease (78 [90.7%]) and stage III disease (346 [81.8%]) who received PCI (*P* = .10). Patients with stage I to II disease achieved longer OS (median, 50 months [95% CI, 38 to not reached months] vs 25 months [95% CI, 21-29 months]; hazard ratio, 0.60 [95% CI, 0.44-0.83]; *P* = .001) compared with patients with stage III disease. In patients with stage I to II disease, no significant survival difference was found between the trial arms (median, 39 months in the once-daily arm vs 72 months in the twice-daily arm; *P* = .38). Apart from lower incidence of acute esophagitis in patients with stage I to II disease compared with patients with stage III disease (grade ≥3, 9 [11.3%] vs 82 [21.1%]; *P* < .001), the incidences of acute and late toxic effects were not significantly different.

**Conclusions and Relevance:**

Patients with stage I to II SCLC in CONVERT achieved long-term survival with acceptable toxic effects after chemoradiotherapy and PCI. This study suggests that patients with stage I to II small cell lung cancer treated with modern chemoradiotherapy have better outcomes compared with patients with stage III disease, providing information that practitioners can potentially give their patients to aid clinical decisions.

**Trial Registration:**

ClinicalTrials.gov identifier: NCT00433563

## Key Points

**Question:**

What are the characteristics and outcomes among patients with stage I to II small cell lung cancer treated with chemoradiotherapy?

**Findings:**

In this secondary analysis of the Concurrent Once-Daily vs Twice-Daily Radiotherapy Trial, among 509 patients, those with stage I to II small cell lung cancer achieved longer overall survival than did patients with stage III disease, with acceptable adverse effects after chemoradiotherapy and prophylactic cranial irradiation.

**Meaning:**

The findings suggest that patients with stage I to II small cell lung cancer treated with modern chemoradiotherapy have better outcomes compared with patients with stage III disease, providing information that practitioners can potentially give to their patients to aid clinical decisions.

## Teaser

This secondary analysis of a randomized clinical trial examines the association of modern chemoradiotherapy with survival among patients with stage I to II small cell lung cancer compared with that among patients with stage III small cell lung cancer.

## Introduction

Small cell lung cancer (SCLC) accounts for 10% to 20% of lung cancer cases.^1^ A 2-tier staging system, introduced by the Veterans Administration Lung Cancer Study,^2^ classifies disease into limited or extensive stage according to whether the tumor is localized to one hemithorax. On the basis of several meta-analyses and landmark trials,^3,4,5,6,7^ standard treatment for limited-stage SCLC is chemoradiotherapy using twice-daily radiotherapy delivered early with chemotherapy. Patients with treatment response should also be offered prophylactic cranial irradiation (PCI).^8^

The Union for International Cancer Control/American Joint Committee on Cancer (UICC/AJCC), based on an International Association for the Study of Lung Cancer analysis, recommended the use of TNM staging for SCLC in 2009 because it provides additional prognostic information.^9,10^ Patients with limited-stage SCLC are a heterogeneous population that includes early (TNM stages I-II) and locally advanced (TNM stage III) tumors.

To date, there are no data on the proportion or outcome of patients with stage I to II disease included in trials that established chemoradiotherapy as standard treatment,^6,11,12^ limiting the evidence to guide stage I to II SCLC management. Limited evidence supports a role for surgical resection in patients with resectable stage I to II SCLC,^13,14,15,16,17,18,19,20,21^ which is reflected in oncology practice guidelines.^22,23,24^ However, there is no consensus on the optimal postsurgery adjuvant therapeutic approach for these patients.^25,26^ Thus, the role of surgery in stage I to II SCLC is not clearly defined, and international practice is variable.^17,25^

The outcome of patients with stage I to II SCLC treated with chemoradiotherapy has not been reported, to our knowledge, since the adoption of TNM staging, the use of fludeoxyglucose F 18–labeled positron emission tomography (FDG-PET) for staging, the omission of elective nodal irradiation, and the widespread use of conformal radiotherapy. To address these issues and inform clinical practice, we performed a secondary analysis of the Concurrent Once-Daily vs Twice-Daily Radiotherapy Trial (CONVERT) (ClinicalTrials.gov identifier: NCT00433563) to establish the characteristics, treatment, and outcomes among patients with stage I to II SCLC. CONVERT was a randomized clinical trial that assigned patients with limited-stage SCLC and good performance status to receive twice-daily or once-daily radiotherapy concurrently with chemotherapy. CONVERT was designed to establish a standard chemoradiotherapy regimen for limited-stage SCLC, demonstrating that survival outcomes were not significantly different between twice-daily and once-daily radiotherapy, with lower than expected toxic effects.^4^

## Methods

### Study Design

Full details of the CONVERT design were previously published.^27^ In summary, CONVERT was a multicenter, international, randomized, phase 3 trial. Eligible patients were 18 years or older, had histologically or cytologically confirmed limited-stage SCLC (Veterans Administration Lung Cancer Study definition),^2^ and had an Eastern Cooperative Oncology Group performance status of 0 to 1. Patients with a performance status of 2 because of cancer-related symptoms were included at the discretion of the local investigator. All patients underwent baseline physical examination, chest radiography, thorax and upper abdominal computed tomography (CT), brain imaging (CT or magnetic resonance imaging), and complete blood cell count and biochemical profile. Patients were required to have satisfactory pulmonary functions (forced expiratory volume in 1 second >1 L/40% predicted, transfer factor for carbon monoxide >40% predicted). A maximum of one of the following adverse serum biochemical findings was allowed: alkaline phosphatase level more than 1.5 times the upper limit of normal, sodium level less than the lower limit of normal, and lactate dehydrogenase level greater than the upper limit of normal. Staging FDG-PET was allowed but not mandated. This trial was conducted following National Research Ethics Service Committee North West–Greater Manchester Central approval in accordance with the Declaration of Helsinki^28^ and good clinical practice guidelines. All patients provided written informed consent, and data were deidentified. Additional trial details can be found in the protocol in Supplement 1.

Tumor and nodal stage data were collected at the time of entry into the trial according to AJCC’s *Cancer Staging Manual*, 7th edition.^29^ This current analysis was an unplanned secondary analysis of patients with TNM stage I to II SCLC because stratification was not performed according to TNM stage in CONVERT. We hypothesized that the outcomes among these patients would be significantly superior to those among patients with stage III disease. We analyzed data from CONVERT to establish the characteristics, treatment, and outcomes of patients with stage I to II SCLC compared with patients with stage III disease.

### Treatment and Follow-up

From April 7, 2008, to November 29, 2013, a total of 547 patients were randomly assigned (using minimization method) 1:1 to receive concurrent twice-daily radiotherapy (45 Gy in 30 twice-daily fractions for 3 weeks, 5 days per week [to convert gray to rad, multiply by 100]) or once-daily radiotherapy (66 Gy in 33 daily fractions for 6.5 weeks, 5 days per week) from day 22 of chemotherapy cycle 1. A total of 543 patients were included in the modified intention-to-treat survival analysis in CONVERT (273 in the twice-daily radiotherapy group and 270 in the once-daily radiotherapy group). Four patients were lost to follow-up because centers did not return their case report forms. Detailed trial results were published previously.^4^ Data analysis for this secondary analysis was performed from November 1, 2017, to February 28, 2018.

Three-dimensional conformal radiotherapy was mandated, and intensity-modulated radiotherapy was permitted. Inhomogeneity corrections were applied during the radiotherapy process. Elective nodal irradiation was not allowed. A radiotherapy quality assurance program managed by the UK National Cancer Research Institute Radiotherapy Trials Quality Assurance team was integrated into this trial.^27^ Chemotherapy was identical in both arms and consisted of 4 to 6 cycles (center choice) of cisplatin, 25 mg/m^2^ intravenously on days 1 to 3 or 75 mg/m^2^ intravenously on day 1, and etoposide, 100 mg/m^2^ intravenously on days 1 to 3 repeated every 3 weeks. Prophylactic cranial irradiation was offered to patients without evidence of progressive disease on CT (within 4 weeks of cycle 4) no later than 6 weeks after the last chemotherapy cycle. The PCI dose and fractionation were left to the discretion of the local investigator. Clinical follow-up assessments consisted of weekly review until resolution of acute adverse effects, then 3-monthly reviews until 1 year after randomization and 6-monthly reviews thereafter. Thorax and upper abdominal CT was required at 6 and 12 months after randomization and thereafter as clinically indicated. Radiologic response was assessed using Response Evaluation Criteria in Solid Tumours.^30^

### Statistical Analysis

The primary trial end point was overall survival (OS) (defined as time from randomization to death from any cause). Secondary end points were local progression-free survival (defined as time from randomization to first clinical or radiologic evidence of local progression or death) and metastasis-free survival (defined as time from randomization to first clinical or radiologic evidence of distant metastasis or death), Common Terminology Criteria for Adverse Events (version 3.0)^31^ toxic effects, and chemotherapy and radiotherapy adherence. Acute toxic effects were defined as those occurring from chemotherapy cycle 1 to 3 months after completion, whereas late toxic effects were defined as those occurring between 3 months and 2 years after completion of treatment.

Baseline and treatment characteristics, acute and late toxic effects, and chemoradiotherapy adherence for patients with stage I to II and stage III SCLC were compared using the χ^2^ or Wilcoxon rank sum test. Kaplan-Meier curves were plotted for each group and survival compared using the Mantel-Cox version of the log-rank test. A competing risk regression analysis, using Fine and Gray’s method,^32^ was used to assess the correlation between stage and site of tumor progression in which the competing event was death. Subdistribution hazard ratios (HRs) and *P* values from the Wald test were reported. For all HRs, the proportionality assumption was assessed using Schoenfeld residual plots. In this subgroup analysis, results were reported for all patients on an intention-to-treat basis. *P* < .05 (2-sided) was considered to be statistically significant. All conducted statistical analyses were reported and were performed in R, version 3.4.1 (https://www.r-project.org).

## Results

A total of 509 of 543 patients (93.7%) with TNM staging information were eligible for this secondary analysis, and 86 of the 509 (16.9%) had TNM stage I to II disease. Of these 86 patients, 4 patients (4.7%) were staged as having TNM stage I disease and 82 (95.3%) as having TNM stage II disease. Thirty-eight patients (44.2%) presented with node-positive disease (N1). A breakdown of tumor and nodal staging for these patients is given in eTable 1 in Supplement 2. Thirty-five patients (40.7%) were randomly assigned to receive twice-daily radiotherapy and 51 (59.3%) to receive once-daily radiotherapy (Table 1). Table 2 gives the baseline and treatment characteristics for both groups. The median gross tumor volume was significantly smaller in patients with stage I to II disease (38.4 cm^3^; range, 2.2-593.0 cm^3^) compared with patients with stage III disease (93 cm^3^; range 0.5-513.4 cm^3^; *P* < .001). No other significant differences were found in baseline and treatment characteristics between the 2 groups or the proportion of patients with stage I to II (78 [90.7%]) and stage III (346 [81.8%]) SCLC who received PCI (*P* = .10). Baseline and treatment characteristics for each trial arm in both groups are given in eTable 2 in Supplement 2.

**Table 1.** Treatment Delivered to Each Group and Number Included in Analysis

Variable	Patients, No. (%)	
	Stage I-II Disease (n = 86)	Stage III Disease (n = 423)
Treatment arm		
Twice daily	35 (40.7)	219 (51.8)
Once daily	51 (59.3)	204 (48.2)
Treatment delivered		
Concurrent chemoradiotherapy	79 (91.9)	382 (90.3)
Sequential chemoradiotherapy	2 (2.3)	7 (1.7)
No radiotherapy	5 (5.8)	34 (8.0)
No. of treatment cycles delivered		
0	1 (1.2)	4 (0.9)
1	4 (4.7)	23 (5.4)
2	2 (2.3)	10 (2.4)
3	7 (8.1)	36 (8.5)
4	55 (64.0)	247 (58.4)
5	2 (2.3)	14 (3.3)
6	6 (7.0)	89 (21.0)
Included in survival analysis	86	423
Included in toxic effects analysis	85	412

**Table 2.** Baseline and Treatment Characteristics of the 2 Study Groups^a^

Characteristic	Patients With Stage I-II Disease (n = 86)	Patients With Stage III Disease (n = 423)	*P* Value
Age, median (range), y	62 (29-77)	62 (34-81)	NA
Sex			
Male	51 (59.3)	226 (53.4)	.38^b^
Female	35 (40.7)	197 (46.6)	
Smoking history			
Never	2 (2.3)	4 (0.9)	.33^b^
Ex-smoker	49 (57.0)	269 (63.6)	
Current smoker	35 (40.7)	150 (35.5)	
ECOG PS			
0	49 (57.0)	184 (43.5)^c^	.06^b^
1	34 (39.5)	226 (53.5)^c^	
2	3 (3.5)	13 (3.1)^c^	
MRC dyspnea score			
0	38 (44.2)	139 (32.9)	.24^b^
1-2	35 (40.7)	215 (50.8)	
3-4	8 (9.3)	40 (9.4)	
Not assessed	5 (5.8)	29 (7)	
Staging FDG-PET			
No	28 (32.6)	189 (44.7)	.10^b^
Yes	58 (67.4)	233 (55.1)^c^	
Not known	0	1 (0.2)^c^	
Planned No. of chemotherapy cycles			
4	60 (69.8)	287 (67.8)	.82^b^
6	26 (30.2)	136 (32.2)	
GTV, median (range), cm^3^	38.4 (2.2-593.0)	93 (0.5-513.4)	<.001^d^
Concurrent	79 (91.9)	382 (90.3)	.72^b^
Sequential	2 (2.2)	7 (1.6)	
No radiotherapy	5 (5.8)	34 (8.0)	
IMRT			
Yes	14 (16.3)	67 (15.8)	>.99^b^
No	72 (83.7)	356 (84.2)	
Prophylactic cranial irradiation			
Yes	78 (90.7)	346 (81.8)	.10^b^
No	6 (7.0)	59 (13.9)	
Missing data	2 (2.2)	18 (4.3)	
Minimum PTV dose, median (range), %	90.0 (32-100)	87.5 (0-100)	.05^d^

Abbreviations: ECOG PS, Eastern Cooperative Oncology Group performance status; FDG-PET, fludeoxyglucose F 18–labeled positron emission tomography; GTV, gross tumor volume; IMRT, intensity-modulated radiation therapy, MRC, Medical Research Council; PTV, planning target volume.

^a^ Data are presented as number (percentage) of patients unless otherwise indicated.

^b^ χ^2^ Test.

^c^ Percentages do not sum to 100% because of approximation.

^d^ Wilcoxon rank sum test.

The survival analysis included 86 patients with stage I to II disease and 423 patients with stage III disease. Median OS was 50 months (95% CI, 38 months to not reached) in patients with stage I to II disease and 25 months (95% CI, 21-29 months) in patients with stage III disease (HR, 0.60; 95% CI, 0.44-0.83; *P* = .001). Two-year OS was 64% (95% CI, 54%-75%) in patients with stage I to II disease vs 51% (95% CI, 46%-56%) in patients with stage III disease. Five-year OS was 49% (95% CI, 39%-62%) in patients with stage I to II disease vs 28% (95% CI, 23%-34%) in patients with stage III disease.

Radiologic tumor response (defined as complete or partial response on any follow-up CT) was not significantly different between patients with stage I to II disease (76%; 95% CI, 66%-83%) and patients with stage III disease (77%; 95% CI, 73%-81%) (*P* = .90). At the time of analysis, 35 of 86 patients (40.7%) with stage I to II disease and 243 of 423 patients (57.4%) with stage III disease had disease progression. eTable 3 in Supplement 2 lists the type of progression sites (ie, locoregional vs distant) in the 2 groups and the patients who were dead at the time of analysis. eTable 4 in Supplement 2 lists the sites of tumor progression in patients with stage I to II disease and patients with stage III disease. Competing risk regression showed that although locoregional progression was similar between the 2 groups (subdistribution HR, 1.29; 95% CI, 0.70-2.35; *P* = .41), distant progression was significantly more common in patients with stage III disease (subdistribution HR, 1.60; 95% CI, 1.02-2.51; *P* = .04). Median local progression-free survival was 38 months (95% CI, 21 months to not reached) in patients with stage I to II disease vs 17 months (95% CI, 15-20 months) in patients with stage III disease (HR, 0.63; 95% CI, 0.46-0.85 months; *P* = .003), and median metastatic progression-free survival was 40 months (95% CI, 24 months to not reached) in patients with stage I to II disease vs 16 months (95% CI, 14-19 months) in patients with stage III disease (HR, 0.58; 95% CI, 0.42-0.79; *P* < .001). These results and additional outcome data are shown in the Figure and Table 3.

**Figure.**
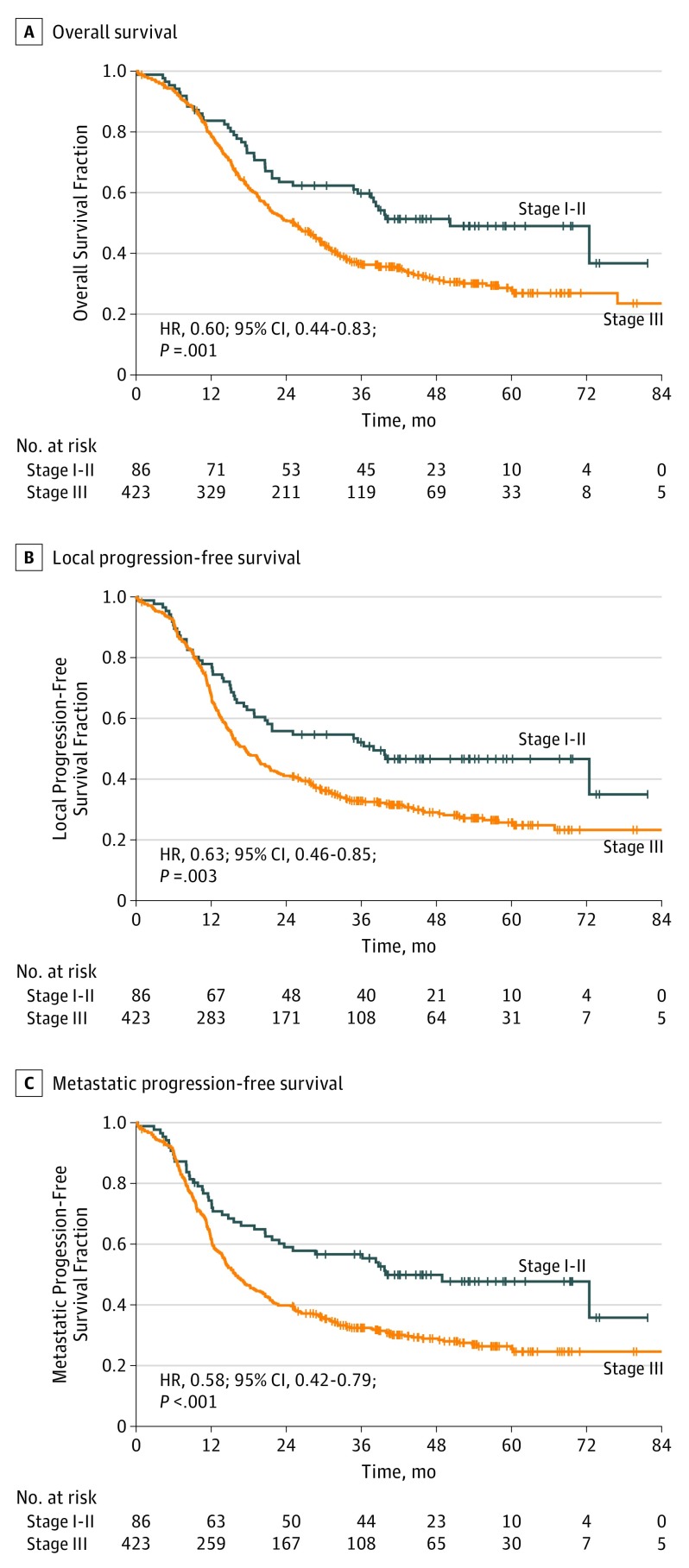
Overall Survival and Local and Metastatic Progression-Free Survival in the 2 Groups Data represent patients treated with both once-daily and twice-daily radiotherapy. HR indicates hazard ratio.

**Table 3.** Comparison of Outcomes Between the 2 Groups

Outcome	Survival, % (95% CI)		Log Rank *P* Value
	Patients With Stage I-II Disease	Patients With Stage III Disease	
Overall survival, y			
1	83 (75-91)	79 (75-83)	.001
2	64 (54-75)	51 (46-56)	
4	51 (42-64)	32 (27-37)	
5	49 (39-62)	28 (23-34)	
Local progression-free survival, y			
1	78 (70-87)	68 (63-72)	.003
2	56 (46-67)	41 (37-46)	
4	47 (37-59)	29 (25-34)	
5	47 (37-59)	26 (21-31)	
Metastatic progression-free survival, y			
1	74 (66-84)	62 (57-67)	<.001
2	59 (49-70)	40 (35-45)	
4	50 (40-62)	29 (25-34)	
5	48 (38-60)	26 (21-31)	

The difference in OS between patients with stage I to II disease and those with stage III disease was upheld irrespective of trial arm (eFigure, A and B in Supplement 2). However, in patients with stage I to II disease, there was no significant difference in OS between trial arms (median of 39 months in once-daily vs 72 months in twice-daily arm; *P* = .38) (eFigure, C in Supplement 2). Similarly, there was no difference in OS between patients with stage I to II disease staged with or without FDG-PET (median of 50 months vs 40 months; *P* = .69) (eFigure, D in Supplement 2).

The optimal number of fractions, as defined per protocol (30 fractions in the twice-daily arm and 33 in the once-daily arm),^27^ were delivered in 69 patients (80.2%) in the stage I to II group and 314 patients (74.2%) in the stage III group (*P* = .60). There were no significant differences in the delivered radiotherapy dose (≥60 Gy to 90 [90.3%] in the once-daily arm vs ≥44 Gy to 362 [85.6%] in the twice-daily arm; *P* = .11) (eTable 5 in the Supplement 2), minimum planning target volume dose (90.0% vs 87.5%; *P* = .05) (Table 2), or delivered number of chemotherapy cycles (4 cycles in 55 [64.0%] vs 247 [58.4%] and 6 cycles in 6 [7.0%] vs 89 [21.0%]; *P* = .98) (Table 1) between patients with stage I to II disease and those with stage III disease.

Apart from a significantly lower incidence of acute esophagitis in patients with stage I to II disease compared with those with stage III disease (grade ≥3, 9 [11.3%] vs 82 [21.1%]; *P* < .001), the incidences of acute and late treatment-related toxic effects were not significantly different between the 2 groups (eTables 6 and 7 in Supplement 2).

## Discussion

Limited-stage SCLC prognosis is poor, with modest survival improvement during the past decades mainly because of radiotherapy advancements and better integration of chemotherapy and radiotherapy.^33^ In this analysis, patients with stage I to II SCLC achieved a median OS of 50 months, with acceptable adverse effects after chemoradiotherapy and PCI. To our knowledge, this is the first study reporting on stage I to II SCLC within a randomized clinical trial in the era of modern radiotherapy. Although our findings were expected, this analysis may benchmark chemoradiotherapy outcome and toxic effects in stage I to II SCLC, providing information that practitioners can relay to their patients to aid clinical decisions.

To our knowledge, there are no randomized data to support PET staging in SCLC. Published studies^34,35,36^ found that FDG-PET upstages a patient proportion and is associated with higher sensitivity (up to 100%) and specificity (up to 83%) compared with CT. However, these results are inconsistent.^37^ A few studies^38,39^ also demonstrated the prognostic value of pretreatment tumor FDG uptake in SCLC. Recent oncology practice guidelines recommend FDG-PET staging in patients with limited-stage SCLC.^22,23,24^ In our analysis, 58 patients with stage I to II SCLC (67.4%) underwent staging with FDG-PET compared with 309 patients (56.9%) overall in CONVERT. We found no statistically significant difference in OS between patients staged with or without FDG-PET. Pretreatment tumor FDG uptake data were unavailable. An analysis of patients staged with or without FDG-PET in CONVERT will be reported in the future.

The roles of surgery and chemoradiotherapy in the management of early limited-stage SCLC have been heavily debated for years.^40^ Randomized clinical trials that established radiotherapy over surgery^41^ and chemoradiotherapy over trimodality treatment^42^ in limited-stage SCLC were conducted more than 20 years ago using currently substandard surgical and radiotherapy techniques.^43^ To our knowledge, there are no contemporary randomized clinical trials comparing chemoradiotherapy with surgery followed by adjuvant therapy for limited-stage SCLC.^26^ Oncology practice guidelines recommend surgical resection for TNM stage T1-2N0 (National Comprehensive Cancer Network^23^) and T1-2N0,1 only after ruling out mediastinal nodal involvement (European Society for Medical Oncology^22^). Published studies^13,14,15,16,17,18,19,20,21^ have reported the role of surgery in patients with limited-stage SCLC. Because of differences in patient selection, staging, and changes in surgical and supportive care standards over time (surgical studies span from 1991 to 2017), it is difficult to compare our findings with findings from those studies.

In line with the main trial,^4^ there was no statistically significant difference in OS between patients with stage I to II SCLC treated with once-daily or twice-daily radiotherapy. However, this study was not designed or powered to compare trial arms among patients with stage I to II disease. For this reason, these results should be regarded as descriptive only. Most patients with stage I to II SCLC (90.7%) in this analysis received PCI, with brain metastasis subsequently detected in 6 patients. Two retrospective series showed that patients with surgically resected SCLC have longer OS after PCI but not patients with stage I disease.^44,45^ However, a recently published population analysis^21^ demonstrated that patients with pT1-2N0M0 treated with surgery alone had worse outcomes compared with those who received adjuvant chemotherapy or chemotherapy and PCI. Cranial irradiation is associated with long-term neuropsychological toxic effects in patients with SCLC.^46^ Omitting PCI could be advantageous in patients with early-stage SCLC who are likely to live longer and experience long-term adverse effects of whole-brain radiotherapy. Neuropsychological toxic effects data after PCI were not collected in CONVERT.

In this trial, treatment was well tolerated in patients with stage I to II SCLC, with a low incidence of severe adverse effects. Grade 3 or higher acute esophagitis was significantly lower in patients with stage I to II SCLC compared with patients with stage III SCLC, likely because of smaller radiotherapy treatment volumes. None of the patients with stage I to II disease had grade 3 or higher acute pneumonitis. The low incidence of severe toxic effects is a valid rationale to consider future radiotherapy dose intensification trials to improve outcomes in this patient group. There were also no reported treatment-related deaths among patients with stage I to II disease.

### Limitations

The main limitations of this study are the unplanned nature of the analysis with a relatively small number of patients, especially for patients with stage I SCLC. This limitation curtailed the investigation of likely prognostic covariates in patients with stage I to II SCLC, such as nodal involvement (N0 vs N1), gross tumor volume, and delivered radiotherapy dose.

## Conclusions

Patients with stage I to II SCLC in CONVERT achieved long-term survival with acceptable toxic effects after chemoradiotherapy. Concurrent chemoradiotherapy followed by PCI may be considered as a treatment option in this patient group. A randomized clinical trial is ultimately required to guide the treatment decision between a surgical and nonsurgical approach for these patients. Innovative translational studies are also required to discover biomarkers that could improve patient selection and delivery of personalized treatment to improve patient outcomes.

## Notes

Supplement 1. Trial Protocol

Supplement 2. **eTable 1.** Tumor and Nodal Staging in Stage I-II Patients **eTable 2.** Baseline and Treatment Characteristics in Stage I-II and Stage III According to Trial Arm **eTable 3.** Type of Tumor Progression in Stage I-II and Stage III **eTable 4.** Site of Tumour Progression in Stage I-II and Stage III **eTable 5.** Comparison of Received Radiotherapy Dose According to Treatment Arm in Stage I-II and Stage III **eTable 6.** Comparison of Acute Toxicity in Stage I-II and Stage III **eTable 7.** Comparison of Late Toxicity in Stage I-II and Stage III **eFigure.** Overall Survival According to Trial Arm and Staging [18F]FDG PET

Supplement 3. Data Sharing Statement
