# Supplementary material for: Association of Chemoradiotherapy With Outcomes Among Patients With Stage I to II vs Stage III Small Cell Lung Cancer: Secondary Analysis of a Randomized Clinical Trial
Source: JAMA Oncol. 2018 Dec 6;5(3):e185335. doi: 10.1001/jamaoncol.2018.5335 (PMC6439849; doi:10.1001/jamaoncol.2018.5335)
Supplement: Supplement 1. — Trial Protocol [file jamaoncol-5-e185335-s001.pdf]

ONC18-1384 - Stage I-II small-cell lung cancer: Subgroup analysis of the Concurrent Once-daily VErsus twice-daily radioTherapy (CONVERT) randomized controlled trial

This supplement contains the following items:

1. Original Protocol Version 1.0, dated 01 Nov 2007
2. Final Protocol Version 3.0, dated 10 JUN 2008
3. Summary of all amendments to the protocols
4. Original and final statistical analysis plan, no changes were made during the course of the trial.

Manchester Lung Cancer Group

CONVERT

Concurrent **ON**ce-daily **VE**rsus twice-daily **R**adio**T**herapy: A 2-arm randomised controlled trial of concurrent chemo-radiotherapy comparing twice-daily and once-daily radiotherapy schedules in patients with limited stage small cell lung cancer (SCLC) and good performance status.

**Protocol Version Number:**	**Final version 1.0**
**Protocol Date:**	**1st November 2007**
**Authorised by:**	[illegible] .... Dr Corinne Faivre-Finn 1.11.2007 .... Date

This document describes the CONVERT clinical trial and provides information about procedures for entering patients. The protocol should not be used as an aide-memoire or guide for the treatment of other patients; every care was taken in its drafting, but corrections or amendments may be necessary. These will be circulated to investigators in the trial, but centres entering patients for the first time are advised to contact the trials centre to confirm they have the most up to date version.

Problems relating to this trial should be referred to the relevant member of the trial management group.

This trial will adhere to the principles outlined in the International Conference on Harmonisation Good Clinical Practice (ICH GCP) guidelines. It will be conducted in compliance with the protocol, the Data Protection Act (DPA G0027154) and other regulatory requirements as appropriate.

PROTOCOL DEVELOPMENT GROUP

Chief Investigator:

Dr Corinne Faivre-Finn
Dept Clinical Oncology
Christie Hospital NHS trust
Wilmslow Road
Manchester
M20 4BX
e-mail:corinne.finn@christie.nhs.uk
telephone: +44 (0)161 446 8290
fax: +44 (0)161 446 8142

Sponsor:

Christie Hospital NHS Trust
Wilmslow Road
Manchester
M20 4BX
telephone: +44 (0)161 446 3000

	Host Institution	e-mail
**Clinical oncologists**		
Professor Allan Price	Western General Hospital, Edinburgh	aprice@staffmail.ed.ac.uk
Dr Michael Snee	Cookridge Hospital, Leeds	michael.snee@leedsth.nhs.uk
Dr Rhona McMenemin	Newcastle	Rhona.McMenemin@nuth.northy.nhs.uk
Dr Nazia Mohammed	Beatson Infirmary, Glasgow	Nazia.Mohammed@NorthGlasgow.Scot.N
**Medical oncologists**		
Dr Fiona Blackhall	Christie Hospital, Manchester	Fiona.Blackhall@christie.nhs.uk
Dr Siow Ming Lee	UCL Hospital, London	siow-ming.lee@uclh.nhs.uk
Dr Paul Lorigan	Christie Hospital, Manchester	Paul.Lorigan@christie.nhs.uk
Professor Penella Woll	Weston Park Hospital, Sheffield	p.j.woll@shef.ac.uk
**Medical Physicist**		
Dr Carl Rowbottom	Christie Hospital, Manchester	Carl.Rowbottom@physics.cr.man.ac.uk
**Pathologist**		
Dr Helen Doran	Wythenshawe Hospital, Manchester	helen.doran@smuht.nwest.nhs.uk
**Chest Physician**		
Dr Jan VanMeerbeeck	University of Gent, Belgium	jan.vanmeerbeeck@ugent.be
**Radiologist**		
Dr Stephen Gwyther	East Surrey Hospital	GwytherSJ@aol.com
**Radiographer**		
Michelle Duffy	Christie Hospital, Manchester	michelle.duffy@christie.nhs.uk
**Patient representative**	Janet Power	

Radiotherapy quality assurance

Ethan Lyn	Mount Vernon Hospital	ethanlyn@mac.com

Research nurse

Lynn Lomax	Christie Hospital, Manchester	Lynn.lomax@christie.nhs.uk

Statisticians

Ric Swindell	Christie Hospital NHS Trust	ric.swindell@christie.nhs.uk
Linda Ashcroft	Christie Hospital NHS Trust	Linda.ashcroft@christie.nhs.uk

Pharmacist

Susan Arrand	Christie Hospital NHS Trust	susan.arrand@christie.nhs.uk

Trial Management

Helen Flight	Christie Hospital, Manchester	helen.flight@christie.nhs.uk

Trial advisor

Richard Stephens	MRC Clinical Trials Unit, London	rs@ctu.mrc.ac.uk

Trial coordination

Manchester Lung Cancer Group
Christie Hospital Cancer Trials Unit
Wilmslow Road
Manchester
England M20 4BX

general enq: +44 (0)161 918 7101
fax: +44 (0)161 446 8148
e-mail: helen.flight@christie.nhs.uk

Randomisation line: +44 (0)161 446 3311

Monday - Friday

09:00 - 17:00 (BST)

**CONTENTS**		Page
	Trial summary	7
	Flowchart	8
1.0	**Background**	9
2.0	**Trial design**	12
2.1	Trial objectives	12
2.2	Overall design	12
2.3	Related sub-studies	12
3.0	**Trial entry**	14
3.1	Centre eligibility	14
3.2	Centre entry criteria	14
3.3	Patient entry	15
3.4	Randomisation	16
4.0	**Treatment plan**	18
4.1	Chemotherapy	18
4.2	Thoracic Radiotherapy	20
4.3	Twice daily thoracic radiotherapy	22
4.4	High Dose once daily thoracic radiotherapy	23
4.5	Prophylactic cranial irradiation	24
4.6	Concomitant medication	24
4.7	Criteria for stopping treatment	25
4.8	Therapy after protocol treatment has stopped	25
4.9	Patients not meeting radiotherapy planning requirements	25
5.0	**Pharmacovigilance**	26
5.1	Definition	26
5.2	Causality	27
5.3	Reporting procedures	27
6.0	**Assessments**	29
6.1	Assessments during treatment	29
6.2	Assessments at follow-up	29
6.3	Cost effectiveness	29
6.4	Summary of protocol assessment visits	30
6.5	Study forms	31
6.6	Data handling	32
7.0	**Statistical considerations**	33
7.1	Sample size calculation	33
7.2	General considerations	33
7.3	Qualifications for efficacy analysis	33

7.4	Qualifications for safety analysis	34
7.5	Qualifications for dose intensity analysis	34
7.6	Patient disposition	35
7.7	Patient characteristics	35
7.8	Concomitant therapy	35
7.9	Subsequent therapy	35
7.10	Treatment compliance	35
7.11	Health outcomes	35
7.12	Stratification	36
8.0	**Informed consent, ethical & regulatory considerations**	37
8.1	Ethical approval	37
8.2	Patient informed consent	37
8.3	Protocol compliance	37
8.4	Indemnity and compensation	37
8.5	Data protection	37
8.6	Duration of the trial	37
8.7	Publication policy	37
9.0	**Independent data monitoring and ethics committee**	38
10.0	**References**	39
11.0	**Glossary**	41
	**Appendices**	
	Appendix 1 - Performance status scale (ECOG)	43
	Appendix 2 – CTCAE v 3.0	44
	Appendix 3 - MRC dyspnoea score	50
	Appendix 4 – Response criteria	51
	Appendix 5 – Cockroft and Gault formula	53
	Appendix 6 - Suggested scheme for administration chemotherapy cycles	54
	Appendix 7 - Chemotherapy agents - safety profiles	55
	Appendix 8 – Expected Adverse Events	59
	Appendix 9 - Compliance with IR(ME)R 2000 Regulations and other QA Requirements	60
	Appendix 10 – Radiotherapy Quality Assurance	61
	Appendix 11 – Site Personnel Form	63
	Appendix 12 – Site Personnel Delegation Log	64

Summary

Type of design

This is a multicentre randomised phase III trial.
Patients are randomised to one of two treatment arms with 1:1 randomisation:
Patients will be randomly allocated to treatment, using permuted blocks within 4 strata.

Patients studied

Histologically or cytologically confirmed SCLC with limited disease (Veterans Administration Lung Cancer Study Group) ie patients whose disease can be encompassed within a radical radiation portal. No pleural or pericardial effusions proven to be malignant. RT target volume acceptable by the local radiotherapist. ECOG performance status 0-2.
For more details refer to section 3.

Trial interventions

Control Arm:
Four to six cycles of Cisplatin 25 mg/m^2 iv D1-3 or 75 mg/m^2 D1 Etoposide 100 mg/m^2 iv D1-3 with concurrent BD radiotherapy 45Gy in 30 twice-daily fractions over 3 weeks, 5 days per week from day 22 of cycle 1

Experimental Arm:
Four to six cycles of_Cisplatin 25 mg/m^2 iv D1-3 or 75 mg/m^2 D1 Etoposide 100 mg/m^2 iv D1-3 with concurrent OD radiotherapy 66Gy in 33 daily fractions over 6.5 weeks, 5 days per week from day 22 of cycle 1

Accrual

At least 532 patients are to be recruited over 4 years.

Outcome measures

Primary end-point

- Overall survival

Secondary end points

- Local progression-free survival
- Metastasis-free survival
- CTCAE v3.0 toxicity
- Chemotherapy dose intensity
- Radiotherapy dose intensity

Duration of assessment

Patients will be assessed prior to each cycle of chemotherapy and at 4 weeks after the final cycle. Follow-up will be at 3 monthly intervals for 12 months and six monthly thereafter until death. Post treatment CT scans thorax and abdomen will be done within 4 weeks of cycle 4 (even if 6 cycles are given) and 6 months after randomisation.

Data collection

Data will be recorded on Case Report Forms (CRFs), a copy should be sent to the Christie Hospital CTU for data entry and a copy kept at the local centre.
For more details refer to section 6.5.

CONVERT trial flowchart

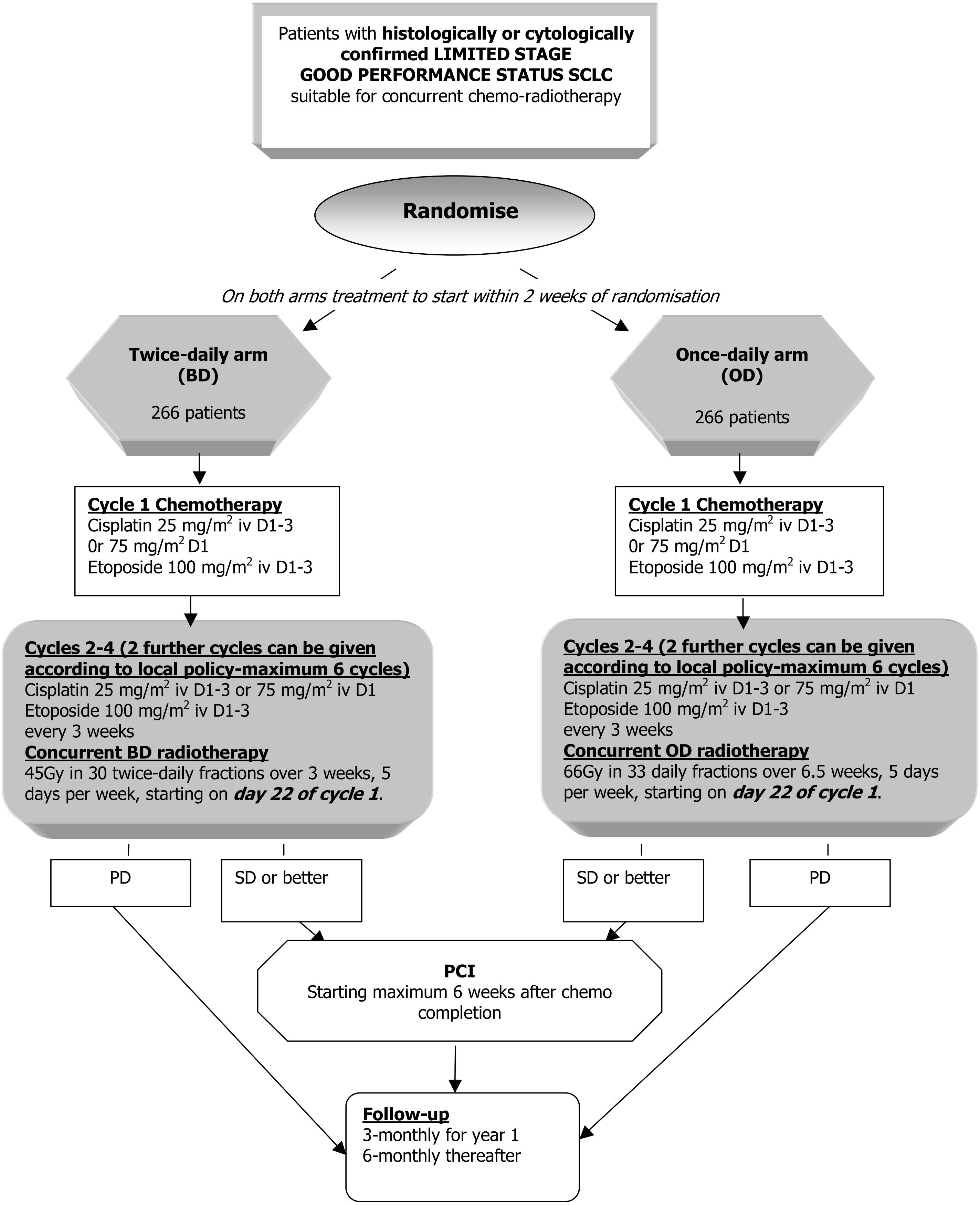

1.0 BACKGROUND

Introduction

Of the 40 000 patients diagnosed with lung cancer each year in Britain, 15 to 20% will have small cell lung cancer. At the time of diagnosis, 30% of patients with small cell carcinoma will have limited stage disease, - that can be encompassed within a tolerable radiation therapy field. However, even in this early stage of disease the outcome is poor, with a median survival of 16 to 24 months with current forms of treatment (1-3).
Combining chemotherapy and thoracic radiotherapy is now the standard treatment for limited stage small cell lung cancer. Indeed two meta-analyses have shown that radiotherapy associated with chemotherapy improves the median survival, the 3 year survival rate and the local control (4,5). In patients who present with small volume limited stage SCLC (ie confined to lung and mediastinum only) there is a real possibility of long-term survival if optimum treatment is given. In this group of patients, a US trial (3) comparing once vs twice daily concurrent chemo-radiotherapy reported >40% survival at 2 years in both arms. This compares with around 15% in the recent UK MRC LU21 trial (6) which used sequential chemo-radiotherapy. There is a real need to improve these UK figures.

Although thoracic radiotherapy is now integrated in the routine treatment of LS SCLC there are numerous questions surrounding the optimum way to combine radiotherapy and chemotherapy. Several important questions remain unanswered including the optimal total radiation dose, radiation fractionation, timing of radiation, sequencing of radiation and volume of irradiation.

Numerous prospective studies have tested various doses, duration, fractionation and timing of thoracic radiotherapy

- Three randomised trials (1,7,8) are in favour of early thoracic irradiation, whereas 3 other randomised studies (9,10,28) did not demonstrate a difference between early and late chest radiotherapy.
- A recent meta-analysis has evaluated early versus late timing of thoracic radiotherapy in combined modality therapy for limited-stage small cell lung cancer. Early radiation therapy was defined as prior to 9 weeks after initiation of chemotherapy (i.e. at the latest with the third cycle of chemotherapy). There was a benefit in overall survival for early radiation therapy versus late radiation therapy (11). In contrast a recent Cochrane review (29) which also included 7 trials (replacing Gregor et al with James et al) concluded that it is unclear whether the timing of chest radiotherapy (beginning within 30 days of the start of chemotherapy or later) affects long term survival. The evidence for starting concurrent radiotherapy with cycle 1 chemotherapy is controversial. In the UK, starting radiotherapy with the first cycle of chemotherapy poses considerable logistical issues. Therefore it appears reasonable to start the radiotherapy treatment during the second or the third cycle of chemotherapy.
- Concurrent chemo-radiotherapy seems to be superior to sequential treatment (7). Indeed if radiotherapy is given early and concurrently with chemotherapy, not only very sensitive cells, but also some less sensitive or highly proliferative cells will be killed, because of the synchronous action of the chemotherapy drugs, together with the effect of radiation. When radiation is given later, often not concurrently with chemotherapy, only the very sensitive cancer cells will be killed by radiotherapy, but not the very rapidly proliferating ones. The Japan Clinical Oncology Group Study (7) is the only trial which has randomised patients between sequential and concurrent chemo-radiotherapy (Sequential: 4 cycles 3-weekly cisplatin 80mg/m2 d1, etoposide 100mg/m2 d1-3, followed by 45Gy/30f BD, Concurrent: 4 cycles 4-weekly cisplatin 80mg/m2 d1, etoposide 100mg/m2 d1-3, with 45Gy/30f BD starting on day 2 of cycle 1). Although the trial showed a survival benefit at 5 years for the concurrent arm (23.7% vs 18.3%) this was not statistically significant (p=0.097) possibly due to the fact that only 228 patients were included in the trial. However the survival benefit was achieved despite the fact that the chemotherapy dose-intensity was inferior in the concurrent arm (given 4-weekly) compared to the sequential arm (given 3-weekly).

- Hyperfractionated radiotherapy has been shown to be more efficacious than standard fractionation in in-vitro studies (13). Fractionating the dose into 2 treatments each day has radiobiological advantages. Small cell lung cancer cells lines are sensitive to small doses of radiation. In addition the use of small doses per fraction will diminish the late damages to the normal tissues. The value of twice daily (BD) thoracic radiotherapy given early and concurrently with PE chemotherapy was examined in a landmark trial (3). The regimens were: once-daily (OD): 4 cycles 3-weekly cisplatin 60mg/m2 d1, etoposide 120mg/m2 d1-3, 45Gy/25f starting d1 of first cycle, BD 4 cycles 3-weekly cisplatin 60mg/m2 d1, etoposide 120mg/m2 d1-3, 45Gy/15f starting d1 of first cycle). This study showed that twice-daily treatment beginning with the first cycle of chemotherapy significantly improves survival as compared with concurrent once daily radiotherapy (p=0.04). After a median follow-up of 8 years, the survival rates for patients receiving once-daily chemotherapy were 41% at 2 years and 21% at 5 years. For patients receiving twice-daily radiotherapy corresponding figures were 47% and 26%. However grade 3 oesophagitis was significantly more frequent with twice-daily radiotherapy, occurring in 27% of patients, as compared with 11% in the once-daily group (p<0.001). No long term strictures were reported.
  One of the main criticisms of the study is that in the once-daily thoracic irradiation arm the rate of local failure was very high (52%), which demonstrated that a total dose of 45 Gy given once-daily is too low. The only other trial comparing OD and BD radiotherapy in this group of patients reported no survival differences although the long-term results for both arms were still encouraging with 5 year survival rates of >20% in each arm (14,15). The regimens compared were OD: 6 cycles 4-weekly cisplatin 30mg/m2 d1-3 and etoposide 100mg/m2 d1-3, with 50.4Gy/28f given concomitantly with chemotherapy cycles 4 and 5, BD: same chemotherapy but with 24Gy/16f BD starting d1 cycle 4, then a 28 day break followed by another 24Gy/16f BD (starting d1 cycle 5). Because of the split-course policy the twice-daily radiotherapy took as long to deliver as conventional once-daily radiotherapy (overall radiotherapy treatment time was 38 days in both arms), resulting in no overall acceleration in the hyper-fractionated arm. An additional potential weakness of the split-course approach is that tumour re-population is likely to occur during the break in treatment allowing chemotherapy resistant clones to develop. The Turrisi trial showed improved survival for the BD arm (47% vs 41% at 2 years, 26% vs 21% at 5 years). However as overall radiotherapy treatment time differed substantially between the two groups (3 weeks for the BD regimen and 5 weeks for the OD regimen), it was unclear whether the survival differences were due to altered fractionation or shorter treatment time or both.

- As far as the total dose of radiation concurrent with chemotherapy is concerned, Choi et al showed that the maximum tolerated dose for thoracic irradiation is at least 70 Gy in 35 fractions over 7 weeks for daily thoracic irradiation and 45 Gy in 30 fractions over 3 weeks for twice-daily thoracic irradiation (16).

Rationale for a phase III trial

The fractionation and overall time of treatment questions have been specifically addressed by the landmark Turissi study published in 1999 (3).
We are currently in the position that as a result of the Takada and Turrisi trials concurrent BD chemo-radiotherapy is widely accepted as standard treatment in the US and Europe for this group of patients. Although the Turrisi trial was published in 1999 it has not been repeated and it is very important to discern whether the survival benefit seen with BD fractionation is a true effect, especially as the RT schedule in control arm of the trial would now be considered sub-optimal, resulting in a very high (52%) local failure rate. More recent studies by Choi et al using once daily radiotherapy and Komaki et al (16, 17) using a concomitant boost technique have suggested that doses of 70Gy over 7 weeks and 61.8Gy over 5 weeks are possible, the former being delivered with 5 cycles of full dose chemotherapy.
Moreover since the Turrisi trial was designed in the late 1980s, progress has been made in radiotherapy techniques. The radiotherapy used in any contemporary trial would be CT-planned conformal treatment with individual blocking, careful calculation of target, lung and oesophageal doses based on modern data on tolerance and response, with verification using on-line portal imaging, correction of set-up errors, and allowance made for the effects of respiratory movement on the position of the target volume. None of these were routine from 1989-1992 when the Turrisi trial was carried out. In addition there has been concern that the toxicity of twice-daily thoracic irradiation concomitant with chemotherapy is too high. In the Turrisi trial grade 3 oesophagitis (defined as inability to swallow more than liquids or to require hospitalisation) occurred

in 27% of patients in the twice-daily group compared with the 11% in the once daily group. However, oesophagitis has not caused permanent or life-threatening damage in the trials using non-anthracycline based regimens.

Before twice-daily thoracic irradiation is accepted as a standard for the treatment of limited stage small cell lung cancer, it is important to compare this schedule with a higher dose of radiation delivered once daily. Indeed the toxicity of twice-daily thoracic irradiation concomitant with chemotherapy is high and the cost and inconvenience of such treatment is also to be considered. Using the modern facilities of conformal 3D radiotherapy and limiting as much as possible the amount of normal tissues irradiated, doses higher than 50 Gy should be evaluated.

In view of the lack of data in the literature addressing the question of the dose and fractionation for limited stage small cell lung cancer, we propose to perform a new randomised phase III trial to try to establish a standard chemoradiotherapy regimen for LS SCLC with good performance status. There are currently no international trials for this group of patients, and thus an opportunity exists to set up a global trial to answer this important question. The results of the trial will be crucial in determining the best international standard treatment for routine clinical use in the treatment of patients with limited-stage SCLC and good performance status. In addition the translational studies carried out in parallel to CONVERT will indicate the hypotheses which need testing in the next generation of trials in this disease.

Choice of chemotherapy regimen

The combination of cisplatin and etoposide (PE) is the standard chemotherapy treatment in this group of patients. The use of PE is supported by 2 reviews: firstly a large systematic review of 7173 randomised patients which found that the inclusion of either cisplatin (22 trials) or etoposide (37 trials) in a chemotherapy regimen was associated with a significant survival improvement, as was the inclusion of both drugs compared to chemotherapy regimens with neither (18), and secondly a meta-analysis of 19 trials of 4054 patients which confirmed an increased survival with the inclusion of platinum of 2.6% and 4.4% at six months and one year respectively (19), and also, in a subset analysis of nine trials, showed a similar survival benefit for the inclusion of etoposide. A subsequent randomised trial of PE vs epirubicin, cyclophosphamide and vincristine in 440 patients supported this conclusion by showing that PE resulted in a better survival (20). Attempts to improve on this combination have met with limited success (21-23). The combination of Etoposide and Cisplatin has become in recent years the treatment of choice as it can be used concurrently at full doses with radiation therapy with acceptable toxicity (1,3,7). It is also simple to use in ambulatory patients.

The Canadian small cell lung cancer guidelines published in 2004 (24) are based on a systematic review and give a range of recommended regimens for concurrent chemo-radiotherapy, including cisplatin 25mg/m2 days 1-3 or 75 mg/m2 d1 with etoposide 100mg/m2 d1-3. There is no good evidence for higher doses of etoposide which result in an increased risk of haematological toxicity, the use of GCSF, and dose delays. The guidelines state that there is insufficient evidence to comment on whether 4 cycles are the same as 6, whether oral is equivalent to iv etoposide, or whether carboplatin can be substituted for cisplatin. Therefore there is a strong rationale for using 4-6 cycles of cisplatin 25mg/m2 d1-3 (or cisplatin 75mg/m2) and etoposide 100mg/m2 d1-3.

Choice of radiotherapy regimen

- BD regimen

Commencing radiotherapy on day 1 of chemotherapy is not feasible as the process of CT planning, volume contouring, treatment optimisation, and set-up checking takes 1-2 weeks. Hence the most feasible BD regimen would be 4-6 cycles of cisplatin 25 mg/m2 d1-3 or 75 mg/m2 d1 and etoposide 100 mg/m2 d1-3, q3w with 45Gy in 30 fractions over 3 weeks commencing on day 1 of cycle 2 of chemotherapy. This would differ from the BD regimen in the Turrisi trial in that the cisplatin dose would be increased (25mg/m2 d1-3 or 75 mg/m2 d1 rather than 60mg/m2 d1), the etoposide dose would be decreased (100mg/m2 d1-3 rather than 120 mg/m2 d1-3), and radiotherapy would start with cycle 2 rather than cycle 1.

- High dose OD regimen

A schedule of 66Gy in 33 daily fractions over 6.5 weeks has been piloted successfully with good compliance and toxicity profile (presented as a poster at the BTOG meeting in Dublin 2005) and is the subject of an existing single centre phase II study in Manchester.

2.0 TRIAL DESIGN

2.1 TRIAL OBJECTIVES

Primary end-point

- Overall survival

Secondary end points

- Local progression-free survival
- Metastasis-free survival
- CTCAE v3.0 toxicity
- Chemotherapy dose intensity
- Radiotherapy dose intensity

2.2 OVERALL DESIGN

This is a multicentre randomised phase III trial.
Patients are randomised to one of two treatment arms with 1:1 randomisation:

A 2-arm randomised trial comparing:

- 45Gy in 30 fractions BD radiotherapy schedule (given concurrently with cisplatin/etoposide)
- 66Gy in 33 fractions OD radiotherapy schedule (given concurrently with cisplatin/etoposide)

2.3 RELATED SUB-STUDIES

- **Correlative molecular studies for the CONVERT trial**

Progress in treatment of SCLC has been hampered by limited understanding of the molecular biology of this disease. It is usually diagnosed on a small biopsy specimen or fine needle aspirate insufficient for detailed molecular studies. Consequently, existing SCLC tumour banks include relatively small series (<100 patients) of samples collected over many years from patients who are heterogeneous with respect to stage and treatment received. The CONVERT trial provides a potentially unique opportunity to prospectively collect a large number of biospecimens from patients of uniform (limited) stage, who are exposed to the same chemotherapy, treated with one of two radiotherapy schedules, and for whom there will be robust clinical outcome data. Although it will still be problematic to obtain large tumour biopsy specimens for many patients, advances in genomic and proteomic technology will enable studies to be performed on blood/serum samples in addition to small biopsy specimens.

- **Biospecimens to be collected**

All patients will be asked to consent for collection of tumour samples (paraffin embedded*)* and blood samples as part of the trial. Blood samples (for genomic and proteomic analysis) will be collected at three timepoints: at baseline prior to any treatment, on day 22 of treatment and on completion of treatment.

- **Molecular studies to be performed**

The objectives of the molecular studies will be to gain improved insight into the molecular biology of SCLC, discover and/or validate candidate biomarkers for response, resistance or toxicity of treatment, and identify novel treatment targets for therapeutic control. The latter is a major objective since the treatments to be evaluated in this trial are anticipated to result in 55% 2 year survival at best. The specific correlative studies have yet to be defined but we anticipate that studies will employ a range of techniques including immunohistochemistry, proteomic profiling (blood/serum) and genetic studies on host and tumour DNA. It is unlikely that there will be sufficient tissue available (and no fresh frozen tissue) for gene expression profiling by cDNA or oligonucleotide microarray. Given that this biobank will be a unique resource the molecular analyses will be conducted where possible taking into account preliminary data and hypotheses generated from studies in SCLC cell lines and xenografts, and from current studies in large patient populations that aim to evaluate host genomic biomarkers of radiation toxicity or cancer predisposition. Samples will be collected globally then stored and analysed in the UK.

3.0 TRIAL ENTRY

3.1 CENTRE ELIGIBILITY

All participating centres must:

a) operate a formal system of Quality Assurance in Radiotherapy (QART), appendix 9.
b) confirm that the initial stage of the radiotherapy quality assurance exercise has been successfully conducted (appendix 10) and that the CONVERT QA questionnaire was completed
c) have conformal radiotherapy planning facilities (defined as multislice CT scanning planning on maximum 5 mm slices, customized blocking, DVH calculations for target and organs at risk, portal image verification) and a radiotherapy verification protocol
d) have discussed suitable patients in a Multi-Disciplinary Meeting, including a lung cancer specialist
e) have a formal protocol for neutropenic sepsis

A centre agreement and checklist must be completed by each centre, to ensure that they are able to comply with the protocol and data collection requirements, prior to site-specific assessment.

3.2 CENTRE ENTRY CRITERIA

3.2.1 Centre Accrediation

The following documentation must be received at the Christie Hospital Clinical Trials Unit in order for an institution to become an approved CONVERT institution:

• Confirmation that the initial stage of the radiotherapy Quality Assurance (QA) exercise has been successfully completed (See Section 3.1)

• Confirmation of favourable local research ethics committee site-specific assessment (SSA)

• A copy of the most recent version of the patient information sheet (appendix 11) and consent form (appendix 12) on local headed paper

• Signed memorandum of understanding (signed by the institution PI)

• Completed Delegation Log (signature list and delegation of responsibilities, appendix 14)

• Full contact details for all site personnel (appendix 13)

Once all of this documentation has been received, confirmation of institution approval will be sent to the Prinicipal Investigator at each institution by the trial team at the Christie Hospital Clinical Trials Unit.

3.3 PATIENT ENTRY

3.3.1 Pre- randomisation evaluation

a) History and examination.
b) Assessment of performance status and dyspnoea score (appendix 1 and 3)
c) CXR
d) Contrasted CT scan of the thorax and upper abdomen (within 4 weeks prior to randomisation)
e) Contrast-enhanced CT (or MRI) scan brain (within 4 weeks prior to randomisation)
f) Pleural aspiration and cytology if possible if patient presents with a pleural effusion
g) Pulmonary function tests [FEV1 and transfer factor (KCO or DLCO/VA)]
h) FBC and serum biochemistry including LDH.
i) Assessment of renal function (see appendix 5)
j) ECG
k) Bone scan need only be performed if there is a specific clinical indication
l) Pregnancy test (if applicable)
m) PET scan or PET-CT, if available. Centres who decide to use PET scans for staging will have to continue doing so for all patients entered in the trial.

3.3.2 Inclusion/exclusion criteria

Queries about eligibility criteria should be addressed prior to randomisation. Patients are eligible for the trial if all of the following criteria are met:

a) Either sex, age ≥18 years
b) Performance status ECOG grade 0-1 (appendix 1). Patients with PS 2 whose general condition is explained by obstructive/bulky disease likely to improve after the first cycle of chemotherapy can be included at the discretion of the local investigator. Patients with PS 2 as a result of comorbid conditions will be excluded.
c) Histologically or cytologically confirmed SCLC
d) No patients with mixed small-cell and non-small-cell histologic features
e) No history of previous malignancy in the last 5 years (except non melanomatous skin or in-situ cervix carcinoma). Patients with previous malignancies (except breast cancer) and in remission for at least 5 years can be included.
f) Limited stage disease (Veterans Administration Lung Cancer Study Group) ie patients whose disease can be encompassed within a radical radiation portal.
g) No pleural or pericardial effusions proven to be malignant
h) RT target volume acceptable by the local radiotherapist
i) Pulmonary function
    a. FEV1 >1 litre or 40% predicted value
    b. KCO (DLCO/VA) >40%predicted
j) Maximum of one of the following adverse biochemical factors:
    a. Serum alkaline phosphatase more than >1.5 times the upper limit of normal (ULN)
    b. Serum sodium < Lower limit of Normal
    c. Serum LDH > ULN

k) Normal serum creatinine and calculated creatinine clearance ≥ 50 ml/min. If calculated creatinine clearance is <50 ml/mn according to the Cockroft and Gault formula (appendix 5), an EDTA clearance should be performed
l) Adequate haematological function
    a. Neutrophils >1.5 x 10^9/l
    b. Platelets >100 x 10^9/l
m) No other previous or concomitant illness or treatment which in the opinion of the clinician will interfere with the trial treatments or comparisons
n) No prior surgical resection of the primary tumour, no prior radiotherapy for lung cancer
o) Considered fit to receive any of the trial regimens
p) Female patients must satisfy the investigator that they are not pregnant, or are not of child-bearing potential, or are using adequate contraception. Men must also use adequate contraception, as etoposide is clastogenic.
q) Patients must not be breastfeeding
r) Patient has read the patient information sheet and has signed the consent form.
s) Patients available for follow-up

3.4 RANDOMISATION

Randomisation will be administered centrally by the trials office at the Christie Hospital. This is a multi-centre randomized phase III trial. Patients will be randomized on a 1:1 basis to one of two treatment arms. The allocation method will be permuted blocks within 4 strata. Randomisation will be implemented in a bespoke computer application at the randomization centre. The factors controlled for in the allocation will be:

Planned number of cycles	(4 or 6)
Performance status	(0/1, 2) {PS2 equivalent to KP50, 60}

Randomisation can only be performed **after** confirmation that the patient is eligible (including recording of LDH, sodium and alkaline phosphatase results) and that the patient has signed consent. The system used does not permit any editing of fields by users after arm allocation has been performed.

Randomisation lines:
Tel: +44 (0)161 446 3311 (open Mon-Fri, 9am - 5pm BST)
Fax: +44 (0)161 446 8148

Randomisation by telephone: The researcher calls the randomisation line and supplies a verbal password for the study. A checklist is checked over the telephone before randomisation. Randomisation will be performed using the computer system, callers will be advised of the patient's trial number and treatment allocation. Please record these on the eligibility checklist and return a copy to the trials office (fax 0161 446 8148). An e-mail confirmation will be sent to pre-arranged e-mail addresses for each centre.

Randomisation by fax: The randomization form must be completed, signed by an authorised member of staff and faxed to the fax randomisation line. On receipt of the fax staff at the trials office will check the eligibility checklist and perform the randomisation. An e-mail confirmation will be sent to pre-arranged e-mail addresses for that centre.

This trial is a comparison of treatment polices, once a patient has been randomised that patient remains in the trial and full documentation is always required. Inevitably some patients will stop treatment early, either

because the patient does not wish to continue or because the responsible clinician feels it is not in the patient's best interest to continue. For patients who stop treatment early the responsible clinician should continue to complete as much clinical information as possible. As a minimum, follow-up data on progression and survival status should be returned regularly (unless explicit consent for this is withdrawn).

4.0 TREATMENT PLAN (For approximate timing of treatments see appendix 6)

4.1 CHEMOTHERAPY

Patients should start chemotherapy within two weeks of randomisation and within two weeks of a clinical assessment of fitness (section 4.1.1). Chemotherapy is cisplatin and etoposide and is given for 4 to 6 cycles. One cycle is given prior to radiotherapy. The second cycle in both arms is given concurrently with radiotherapy if possible. Radiotherapy will start on day 22 of cycle 1. In the BD radiotherapy arm, the third to sixth cycles (if applicable) are given after completion of the thoracic radiotherapy. In OD radiotherapy arm, the third cycle of PE is also given concurrently and the fourth to sixth cycles (if applicable) are given after completion of the thoracic radiotherapy.
Centres will be given the choice to stop chemotherapy after 4 cycles or to continue to up to 6 cycles. Centres who decide to give 6 cycles will have to continue doing so for all patients entered in the trial (unless it is decided that it is not in the patients best interest to receive cycle 5 and 6 or due to patient's choice)

4.1.1 Clinical assessments (see section 6.5)

Assessments should be repeated as necessary, and are required prior to each chemotherapy cycle:

- Clinical examination
- Assessment of performance status (see appendix 1), 'dyspnoea score' (see appendix 3) and toxicity
- FBC, U&Es and LFTs:
    - Bone marrow reserve should be adequate for chemotherapy (i.e. absolute neutrophil count > 1.5 x 10^9/l and platelet count > 100 x 10^9/l)
    - Biochemistry
- Assessment of renal function:
    - Adequate renal function - defined by GFR $\geq$ 50 ml/min (measured by EDTA). The Cockcroft and Gault formula (see appendix 5) may be used to estimate GFR, but if < 50 ml/min then EDTA should be performed.
- Disease status – a CXR will be done prior to cycle 1, 3 and 5 (if 6 cycles of chemotherapy are given). If a CXR shows suspicion of progressive disease a CT scan thorax and abdomen should be done to confirm progression before stopping chemotherapy. A formal assessment with repeat CT scan should be made within 4 weeks after completion of the concurrent chemoradiotherapy.

4.1.2 Chemotherapy Administration

The chemotherapy doses should be based on the patient's calculated pre-treatment body surface area using actual body weight. A detailed plan for administering a cycle of chemotherapy including suggested hydration and anti emetics is given in appendix 6.
In summary, centres can opt for one of the two chemotherapy regimen. Cycles are given at 3 weeks intervals:

- Etoposide 100 mg/m^2 iv D1-3
  Cisplatin 75 mg/m^2 iv D1

Or

- Etoposide 100 mg/m^2 iv D1-3
  Cisplatin 25 mg/m^2 iv D1-3

Patients should be encouraged to take a high oral fluid intake on the day prior to cisplatin chemotherapy.

4.1.3 Dose modifications

The policy should be to delay and give at full dose, rather than reduced dose. The dose modification schedule should be followed, but clinical judgement should be used in individual cases. Common side effects are listed in appendices 7 & 8.

Haematological toxicity

Dose modifications are based on each pre-treatment blood count.

ANC x 10^9/l		Platelets x 10^9/l	Cisplatin /Etoposide
> 1.5	And	>100	Full dose
≤ 1.5	Or	≤ 100	Delay until recovery
Febrile neutropenia or treatment delay for grade 4 neutropenia > 7 days	Or	Grade 4 platelets requiring medical intervention or ≥ grade 2 bleeding with thrombocytopenia	First event: full dose and GCSF support (see paragraph 4.6) is recommended **or** 20% dose reduction<br>Second event: 30% dose reduction<br>Third event: off trial

- If chemotherapy cannot be administered after a three-week delay because of haematological toxicity, chemotherapy should be discontinued.
- Use of GCSF see paragraph 4.6
- If Hb<10 a blood transfusion will be required

Non-haematological toxicity

Hepatic toxicity

AST/ALT		Bilirubin	Cisplatin	Etoposide
2-5 x ULN	and	< 1.5 x ULN	Full dose	Full dose
>5 x ULN	or	>1.5 x ULN	Delay one week then reassess using the same criteria; if delayed for two weeks discontinue	

Renal toxicity

- Request GFR *(EDTA clearance)* before each course of chemotherapy ***If*** creatinine clearance < 50 ml/min
- If GFR re-estimated please dose according to the table:

GFR	Cisplatin	Etoposide
≥ 50 ml/min	Full dose	Full dose
40-49 ml/min	50% dose reduction or substitute with carboplatin AUC 5	20% dose reduction
< 40 ml/min	Off trial	Off trial

Other toxicities (see appendix 2)

Peripheral neuropathy ≥ grade 2	Substitute carboplatin AUC 5 or 50% cisplatin dose reduction after recovery to ≤ grade 1; 100% dose of etoposide
Any grade 3-4 toxicities except mucocitis and alopecia	25% dose reduction for cisplatin and etoposide after recovery to ≤ grade 1

4.1.4 Carboplatin dosing

If carboplatin is to be used, the dose uses target area under the curve of 5 and should be calculated according to the Calvert formula:

Dose (in mg) = Target area under curve x (GFR+ 25)

Carboplatin and etoposide will be given 3 weekly

4.2 THORACIC RADIOTHERAPY

Thoracic radiotherapy should start 21 days after day 1 of the first cycle of chemotherapy (day 22). A delay with the administration of the second cycle of chemotherapy **should not delay** the start of radiotherapy

4.2.1 Clinical assessment (see section 6.5)

Weekly assessments are required during treatment.

- Clinical examination
- Haematological assessments.

4.2.2 General radiotherapy details

Patients should be treated on a linear accelerator operating at 4-10 MV.

The total dose of radiotherapy will be

- BD arm: 45 Gy in 30 twice daily fractions of 1.5 Gy
- OD arm: 66 Gy in 33 daily fractions of 2 Gy

The total dose is prescribed at the ICRU reference point and given according to the recommendations of the EORTC radiotherapy group (25) and ICRU 50 (26). Treatment will be planned with inhomogeneity corrections.

IMRT will be permitted for the centres routinely using it for the treatment of lung cancer.

i. **Radiation Quality Assurance:**

The radiotherapy quality assurance program will be run by the Mount Vernon Hospital QA team (appendix 10). Planning information, copies of portal images (digital print-outs are acceptable) and

dose distribution, including Dose Volume Histogram (DVH), with a copy of the treatment prescription should be available for central review. Details of radiotherapy practice will be established by completion of a questionnaire.

Treatment machine beam output data on the linear accelerators used to treat patients in the trial should have been audited by a recognised ASTRO/ESTRO or UK quality assurance programme within 12 months of the first patient in the centre entering the trial, and be rechecked at least once every 18 months

We recommend that daily verifications should be done with open orthogonal images incorporating stable anatomical structures such as the spine for the first 3 days of treatment followed by weekly verifications. If available cone beam imaging can be used as an alternative to open orthogonal images. The orthogonal images will be checked by a senior radiographer and if possible reviewed by a qualified radiation oncologist, and compared with either the digitally reconstructed images or simulator images. The correction decision will be left to local policy. Differences of less than 0.5 cm from the initial images will be allowed. We suggest that if the difference is more than 0.5 cm the orthogonal images will be repeated prior to patients treatment. The Radiotherapy Quality Assurance Group will approve and monitor each centre procedures.

ii. Patient treatment position:

Supine, with arms above head. Immobilisation using chest board and fixed arm position. The patient should be breathing normally.

iii. Patient data acquisition:

A planning CT scan should be performed in the treatment position, whilst the patient undertakes a normal respiration, using 5 mm slices or less through the entire target volume. The whole thorax (cricoid to L2) should be covered using at least 1 cm slices to allow dose-volume histograms to be calculated for the lung, heart and the oesophagus.
Radiotherapy should be started within 3 weeks of planning.

iv. Planning target volume (PTV)

The CT data will be transferred to the planning system.

- The GTV (gross tumour volume) will be contoured by a qualified radiation oncologist specialised in thoracic malignancies. The contouring should be carried out using the mediastinal and the lung windows. The GTV is defined as identifiable tumour and involved lymph nodes (nodal involvement on CT scan is defined as nodes ≥ 1 cm in short axis). If PET scan is available for staging, the GTV should include PET positive lymph nodes.
- The CTV (clinical target volume) comprises the GTV with a 0.5 cm margin of radiologically normal tissue in all directions. It will take into account microscopic spread. Manual adjustment of CTV is permitted to reduce dose to the spinal cord for example, when disease is adjacent to a structure such as a vertebra but is not thought to invade the structure
- The PTV comprises the CTV with a 1 cm margin superiorly and inferiorly, and 0.8 cm margin laterally, at the 95% isodose. The CTV to PTV expansion should not be reduced as it is allowing for set up errors and organ motion.

Prophylactic nodal irradiation should not be employed.
Field reductions will not be allowed.

v. Treatment planning

Use of 3D conformal technique is required and beam's eye views may be useful in the design of individual shielding. Dose volume histograms (DVH) for the PTV, normal lung, oesophagus, spinal cord and heart will be calculated in order to obtain full knowledge of the 3D dose distribution.

vi. Dose specification and fractionation:

The dose will be specified at the ICRU reference point and fully corrected for heterogeneity. The dose distribution within the PTV should ideally be within ± 5% of the prescribed dose, and no more than ± 7% of the prescribed dose.

vii. Definition of the organs at risk

The spinal cord, lungs, oesophagus and heart will be contoured for dose-volume histograms.

- Both the right and left lungs should be contoured as one structure. Contouring should be carried out using pulmonary windows. All inflated and collapsed lung should be contoured.
- The spinal cord will be contoured based on the bony limits of the spinal canal. The spinal cord should be contoured starting at least 10 cm above the superior extent of the PTV and continuing on every CT slice to at least 10 below the inferior extent of the PTV.
- The oesophagus will be contoured using mediastinal windowing on CTscan to correspond to the mucosal, submucosa, and all muscular layers out to the fatty adventitia. The oesophagus should be fully contoured (from cricoid cartilage to the gastro-oesophageal junction)
- The heart will be contoured along with the pericardial sac. The superior aspect (or base) for purposes of contouring will begin at the level of the superior aspect of the left atrium and extend inferiorly to the apex of the heart.

viii. Beams

Isocentric treatment technique.
The number of beams will vary according to the position and the volume of the PTV in the thorax and to the maximum dose tolerated by the organs at risk.
The treatment plan will be checked by a qualified radiation oncologist after discussion with the planning team. The dose-volume histogram will help to guide that choice.

ix. Set-up verification

Cone beam or orthogonal images will be obtained on days 1 to 3 (or 2 to 4) and weekly thereafter.

4.3 TWICE DAILY THORACIC RADIOTHERAPY

4.3.1 Schedule

- **45 Gy** in **30 twice daily fractions** over a period of **19 days** (radiotherapy to start on a Monday)**,** 5 consecutive days a week
- The optimal overall treatment time should be 19 days, up to 21 days is a protocol deviation and should be recorded, above 21 days is a protocol violation.
- The interfraction interval will be 6 to 8 hours.
- using conformal radiotherapy and 4 to 10 MV photons emitted from linear accelerators.
- Thoracic radiotherapy will start on cycle 1 day 22, if possible concurrently with the second cycle of chemotherapy (within 24 hours of day 1, cycle 2 of PE).

- Concurrent chemotherapy will be administered during the intervals between the 2 daily radiotherapy fractions.

4.3.2 Normal tissue constraints

To reduce late damages to the normal tissues the following rules will be applied

- Dose: **1.5 Gy** per fraction
- Maximum spinal cord dose will not exceed **42 Gy.** The spinal cord position must be identified throughout the PTV
- The percentage of lung minus PTV receiving more than 20 Gy will not exceed **35%** (V20=35%, based on dose-volume histograms). The mean lung dose will be recorded
- The heart can receive the total dose (TD) to < 30% of its volume. For > 50% of cardiac volume, dose < 50% of TD is recommended

4.3.3 Treatment Delays

Every effort should be made to deliver the prescribed dose of radiotherapy in 19 days.

If unavoidable delays occur, that could increase the overall treatment time beyond 19 days, e.g. due to machine breakdown, compensation should if possible be made by one of the following mechanisms:

- treating on a weekend day, ***or***
- adjusting fraction size to deliver the total prescribed dose within 19 days;
  However, fraction size should remain < 2.25 Gy

If the radiation schedule is interrupted for more than 1 week due to intercurrent illness consideration should be given to discontinuing treatment. Further treatment will depend upon the clinical situation and is at the discretion of the responsible clinician. Interruptions for < 1 week due to intercurrent illness or radiation toxicity will be recorded and treatment should be completed as planned.

4.4 HIGH DOSE ONCE DAILY THORACIC RADIOTHERAPY

4.4.1 Schedule

- **66 Gy** in **33 daily fractions** over a period of **45 days** (radiotherapy to start on a Monday)**,** 5 consecutive days a week
- The optimal overall treatment time should be 45 days. Up to 47 days is a protocol deviation and should be recorded, above 47 days is a protocol violation.
- using conformal radiotherapy and 4 to 10 MV photons emitted from linear accelerators.
- Thoracic radiotherapy will start on cycle 1 day 22, if possible concurrently with the second cycle of chemotherapy (within 24 hours of day 1, cycle 2 of PE).

4.4.2 Normal tissue constraints

To reduce late damages to the normal tissues the following rules will be applied

- Dose: **2 Gy** per fraction
- Maximum spinal cord dose will not exceed **48 Gy.** The spinal cord position must be identified throughout the PTV
- The percentage of lung minus PTV receiving more than 20 Gy will not exceed **35%** (V20=35%, based on dose-volume histograms). The mean lung dose will be recorded.
- The heart can receive the total dose (TD) to < 30% of its volume. For > 50% of cardiac volume, dose < 50% of TD is recommended

4.4.3 Treatment Delays

Every effort should be made to deliver the prescribed dose of radiotherapy in 45 days.

If unavoidable delays occur, that could increase the overall treatment time beyond 45 days, e.g. due to machine breakdown, compensation should if possible be made by one of the following mechanisms:

- giving two fractions on a subsequent day, with a minimum interval of six hours between fractions, ***or***
- treating on a weekend day, ***or***
- adjusting fraction size to deliver the total prescribed dose within 45 days;

However, fraction size should remain < 3 Gy.

If the radiation schedule is interrupted for more than 1 week due to intercurrent illness consideration should be given to discontinuing treatment. Further treatment will depend upon the clinical situation and is at the discretion of the responsible clinician. Interruptions for < 1 week due to intercurrent illness or radiation toxicity will be recorded and treatment should be completed as planned.

4.5 PROPHYLACTIC CRANIAL IRRADIATION

No later than 6 weeks after the last cycle of chemotherapy , patients without evidence of progressive disease on CXR or CT scan and with no clinical evidence of brain metastases will be given PCI.
Simulation is mandatory for whole brain irradiation. Patients should be treated in supine position. Immobilisation by individual masks or other means is recommended. Treatment will be delivered with megavoltage machines of energies ranging from 4-10 MV photons. Treatment with a single beam is not acceptable. Doses are specified at the mid-plane of two opposed lateral whole brain fields, prescribed to the isocenter. The dose and fractionation of PCI will be left to the discretion of each principal investigator to allow for variation in local practice.

4.6 CONCOMITANT MEDICATION

- Prophylactic antibiotics with fluoroquinolones (choice left to local investigator) are recommended to start on day 8 of cycle 1 chemotherapy and to continue for 7 days to minimise the risk of neutropenic sepsis and respiratory infection. Consideration should be given to continuing prophylactic antibiotics after subsequent cycles if bronchial obstruction is present.

- Anti-emetics will be routinely used using a combination of a steroid and a 5-HT_3 antagonist.

- The use of GCSF is optional
    - GCSF will be prescribed, if clinically indicated, as per ASCO 2006 guidelines or in line with local policy / guidelines for treatment of febrile or prolonged neutropenia.
    - Secondary prophylaxis with GCSF is recommended
        - weight<70 kg: 300 µg days 5 to 12
        - weight>70 kg: 5 µg/kg days 5 to 12
    - GM-CSF will not be used.

- The use of erythropoietin for the treatment of anaemia will not be permitted

4.7 CRITERIA FOR STOPPING TREATMENT

Protocol treatment may be stopped in the following instances:

- Evidence of progressive disease according to RECIST criteria on CT scan (appendix 4)
- Unacceptable toxicity
- An early toxicity assessment (after 20 patients have completed treatment in each arm) for safety reasons will be carried out. Data will be reviewed by the TMG.
- Intercurrent illness which, in the clinician's opinion, would require discontinuation of protocol therapy.
- If subsequent histological/cytological review is contrary to the original diagnosis
- Patient's request

For patients with measurable disease, evidence of minor disease progression (stable disease by RECIST criteria) is not a requirement to stop treatment in an otherwise clinically and symptomatically stable patient.

4.8 THERAPY AFTER PROTOCOL TREATMENT HAS STOPPED

Treatment after relapse or on withdrawal of patient from protocol treatment is at the discretion of the participating clinician, as deemed appropriate according to the clinical situation. Palliative radiotherapy to areas outside the original radiotherapy treatment volume and second-line chemotherapy can be used according to local practice.

4.9 PATIENTS NOT MEETING THE RADIOTHERAPY PLANNING REQUIREMENTS

If the V20 lung, maximum length of oesophagus receiving 40 Gy, V35 oesophagus, dose to the spinal cord do not meet the protocol requirements, the treatment decision will be left to the local investigator, as some adjustments may be necessary regarding the radiotherapy treatments (eg total dose prescribed, margins…)

These patients, who would have been randomised prior to the first cycle of chemotherapy, will be included in the intention to treat analysis. A subanalysis will be done excluding patients with protocol violations.

5.0 PHARMACOVIGILANCE

5.1 DEFINITIONS

Adverse Event (AE): any untoward medical occurrence in a patient or clinical trial subject administered a medicinal product and which does not necessarily have a causal relationship with this treatment. *An AE can therefore be any unfavourable and unintended sign (including an abnormal laboratory finding), symptom, or disease temporally associated with the use of an investigational medicinal product (IMP), whether or not considered related to the IMP.*

Adverse Reaction (AR): all untoward and unintended responses to an IMP related to any dose administered. *All AE's judged by either the reporting investigator or the sponsor as having reasonable causal relationship to a medicinal product qualify as adverse reactions. The expression reasonable causal relationship means to convey in general that there is evidence or argument to suggest a causal relationship.*

Unexpected Adverse Reaction: an AR, the nature and severity of which is not consistent with the applicable product information (i.e. summary of product characteristics (SmPC) for cisplatin and etoposide which are authorised products*). When the outcome of the adverse reaction is not consistent with the applicable product information this adverse reaction should be considered as unexpected. Side effects documented in the SmPC, which occur in a more severe form that anticipated, are also considered to be unexpected.*

Serious Adverse Event (SAE) or **Serious Adverse Reaction** (SAR) **or Suspected Unexpected Serious Adverse Reaction** (SUSAR) any untoward medical occurrence or effect that at any dose **(please report to chief investigator within 24 hours, with the exception of neutropenic sepsis episodes which are to be documented on the CRF)**

- causes **grade 3 and 4 radiation oesophagitis**
- causes **grade 3 and 4 radiation pneumonitis**
- results in death
- is life-threatening*
- requires hospitalisation**, or prolongation of existing hospitalisation.
- results in persistent or significant disability or incapacity
- is a congenital anomaly or birth defect

Medical judgement should be exercised in deciding whether an AE/AR is serious in other situations. Important AE/AR's that are not immediately life-threatening or do not result in death or hospitalisation but may jeopardise the subject or may require intervention to prevent one of the other outcomes listed in the definition above, should also be considered serious.

*The term 'life-threatening' in the definition of 'serious' refers to an event in which the patient was at risk of death at the time of the event; it does not refer to an event which hypothetically might have caused death if it were more severe.
** Hospitalisation is defined as an inpatient admission, regardless of length of stay, even if the hospitalisation is a precautionary measure for continued observation. Hospitalisations for a pre-existing condition, including elective procedures that have not worsened, do not constitute an SAE.

5.2 CAUSALITY

Most adverse events and adverse drug reactions, that occur in this trial, whether they are serious or not, will be expected treatment related toxicities due to either of the drugs used in this trial (see appendix 8 for list). The assignment of causality should be made using the definitions in the table below.

If any doubt about the causality exists the investigator should inform the trials centre who will notify the Chief Investigators. The pharma company/ies and/or other clinicians may be asked to advise in difficult cases.

In the case of discrepant views on causality between the investigator and others, all parties will discuss the case.

Relationship	Description	RESPONSE
Unrelated	There is no evidence of any causal relationship	Yes or No
Unlikely	There is little evidence to suggest there is a causal relationship (e.g. the event did not occur within a reasonable time after administration of the trial medication). There is another reasonable explanation for the event (e.g. the patient's clinical condition, other concomitant treatment).	Yes or No
Possible	There is some evidence to suggest a causal relationship (e.g. because the event occurs within a reasonable time after administration of the trial medication). However, the influence of other factors may have contributed to the event (e.g. the patient's clinical condition, other concomitant treatments).	Yes or No
Probable	There is evidence to suggest a causal relationship and the influence of other factors is unlikely.	Yes or No
Definitely	There is clear evidence to suggest a causal relationship and other possible contributing factors can be ruled out.	Yes or No
Not assessable	There is insufficient or incomplete evidence to make a clinical judgement of the causal relationship.	Yes or No

5.3 REPORTING PROCEDURES

All adverse events/reactions should be reported. Depending on the nature of the event the reporting procedures below should be followed. Any questions concerning adverse event reporting should be directed to the trials centre in the first instance.

5.3.1 Non-serious adverse events

All toxicities, whether expected or not, should be recorded in the toxicity section of the relevant case report form. These should be reported on the case report forms and sent to the Trial Centre within one month of the form being due. Progressive disease is not considered to be an adverse event.

5.3.2 Serious adverse events

SAEs and SARs fall into two separate categories:

a) SAEs Not Requiring Immediate Reporting

A list of reactions that are expected with the agents used in this trial, or with thoracic radiotherapy, are listed in appendix 8. **Reactions listed in appendix 8 do NOT require immediate reporting**. These reactions should be recorded in the toxicity section of the relevant case report form.

The most recent Summary of Product Characteristics (SPC) for each of the agents being used in this trial is listed in appendix 7. SPC's of the agents can also be obtained from:http://www.medicines.org.uk/.

b) SAEs requiring Immediate Reporting

All SAEs and SARs not listed in appendix 8 must be reported immediately by the local Investigator to the Chief Investigator. The site should:

- Either, complete the SAE case report form & send it immediately (within 24 hours or the next working day, preferably by fax 0161 446 8148), signed and dated to the trial centre together with relevant treatment forms and anonymised copies of all relevant investigations.
- Or, contact the Trial Centre by phone and then send the completed SAE form to the Trial Centre within the following 24 hours as above.

An SAE form should be completed for all SAEs (as defined in section 5.1, but excluding those listed in appendix 8). Progressive disease or death due to SCLC, and hospitalisations for elective treatment of a pre-existing condition, or for expected morbidities associated with treatment do not need reporting as SAEs - unless they are life threatening or fatal. If death occurs within 4 weeks of treatment, consideration must be made, by the responsible consultant, of whether the treatment has caused or contributed to the death. Any death which is suspected of being related to trial treatment must be notified immediately.

Fatal or life threatening SAE's should be reported on the day that the site is aware of the event. The form asks for nature of event, date of onset, severity, corrective therapies given, outcome and causality (i.e. unrelated, unlikely, possible, probably, definitely and not assessable). The responsible investigator should sign the causality of the event. Additional information should be sent within 5 days if the reaction has not resolved at the time of reporting.

The Trial Centre will notify the Main REC of all SUSARs occurring in the trial within 15 days of notification, and will provide the Main REC with an annual report of all SAEs. Investigators should report any SAEs and/or SARs as required by their Main Research Ethics Committee and/or Research & Development Office.

To report an SAE, an SAE form must be completed and returned **within 24 hours** of the clinician becoming aware of the event.

Fax: 0161 446 8148 for the attention of Dr C Faivre-Finn

Please note, all toxicities (whether or not they are SAEs) should also be recorded in the toxicity sections of the relevant case report forms.

6.0 ASSESSMENTS

6.1 ASSESSMENTS DURING TREATMENT

Please see section 4.0 for additional clinical visits required during treatment for each arm. During treatment the following information will be required and relevant details reported on case report forms (3-weekly):

- Clinical examination, performance status (see appendix 1), dyspnoea score (see appendix 3) and weight
- Haematology and biochemistry results
- Calculated creatinine clearance before each cycle of chemotherapy (see section 4.1.1)
- Details of any non cancer related treatment received
- Assessment of toxicity graded according to CTCAE v3.0 (see appendix 2)

6.2 ASSESSMENTS AT FOLLOW-UP

After completion of treatment, patients should be reviewed weekly until acute side effects have resolved then 3 monthly until 1 year, 6 monthly thereafter.

Follow up visits at more frequent intervals should be undertaken at the discretion of the participating clinician.

Assessments to include:

- Clinical examination, performance status (see appendix 1), dyspnoea score (see appendix 3) and weight
- Assessment of any continuing or new toxicity (see appendix 2)
- Assessment of disease status
- Assessment of response by a contrasted CT scan thorax and abdomen within 4 weeks of cycle 4 (even if 6 cycles are given) and 6 months after randomisation
- Details of any additional cancer treatment received
- Late toxicity (CTCAE v3.0)

Participating sites should make all necessary efforts to ensure that follow-up information is obtained for all participants e.g. this may include methods such as tracking the patient through a national cancer registry, GP records network etc if contact is lost.

6.3 COST EFFECTIVENESS

The radiotherapy schedules being compared may have different adverse event profiles and efficacy, and that might translate into different costs, effects and cost-effectiveness.
Therefore the following data will be collected routinely on clinical report forms:

- details of protocol medications
- use of antibiotics, blood & platelet transfusions, GCSF
- management of serious adverse events
- hospital admission episodes

6.4 Summary of protocol assessment visits

PROTOCOL ACTIVITIES	SCREEN	CYCLE 1 TREATMENT	ADDITIONAL CYCLES			END OF TREATMENT (within 4 weeks of last cycle)	FOLLOW-UP[f]
			Chemotherapy – both arms (3 weekly)	Once Daily XRT Arm (45 days)	Twice Daily XRT Arm (19 days)		
**Check eligibility**	X						
**Informed Consent**	X						
**Medical History**	X						
**Physical Examination**	X	X	X[a]			X	X
**Pulmonary function tests**	X						
**Bronchoscopy or CT guided biopsies**	X						
**Pleural Aspiration / cytology**	X[d]						
**CT Planning Scan**	X						
**Randomisation**	X						
**Haematology**	X	X	X[a]			X	
**Biochemistry**	X	X	X[a]			X	
**Creatinine Clearance**	X	X	X[a]				
**CXR**	X	X	Prior to cycle 3,5				X
**ECG**	X						
**CT Scan– Chest/Upper Abdo**	X[e]					After cycle 4	X (6 months only or on relapse)
**CT/MR Scan-Brain**	X [e]						
**Chemotherapy**		X	X[b]				
**Radiotherapy**				X	X		
**PCI (if indicated)**						X[c]	
**Adverse Events/Toxicities**	X	X (baseline)	X	X	X	X	X
**Radiation Toxicities**			X	X	X	X	X
**Research blood specimen**		X				X	
**Tumour sample**		X				X	X (relapse)

[a] Measured as per local policy but at a minimum of 3-weekly
[b] Cycle 1 of chemotherapy to start within 2 weeks of randomisation and assessment of clinical fitness
[c] PCI- to be performed a maximum of 6 weeks after chemotherapy completion
[d] Pleural aspiration/ cytology- to be performed if possible in patients with pleural effusion
[e] Screening chest/ upper abdo CT & screening brain CT/MR- to be performed within 4 weeks prior to randomisation
[f] Follow-up visit scheduling- weekly review until resolution of acute side effects, then 3-monthly until 1 year post randomisation, 6 monthly thereafter (or more frequently at discretion of the investigator)

6.5 STUDY FORMS

**Eligibility checklist**	To be completed at the time of randomisation, prior to phoning the trials office.	**Form Pre-a**
**Pre-treatment form**	To be completed at baseline prior to cycle 1	**Form Pre-b**
**Tumour assessment form**	To be completed at baseline prior to cycle 1	**Form Pre-c**
**Treatment form**	To be completed on day 1 of each cycle. Data collection includes: protocol treatment(s) received, toxicity, reasons for reduction/delay/omission, performance status<br><br>***Complete forms for all cycles (1-4 or 6)***	**Form A**
**Toxicity form**	To be completed at the end of each cycle given, prior to next cycle and 30 days after completion of last cycle of chemotherapy<br><br>***Complete forms post cycle to capture all data***	**Form B**
**Radiotherapy worksheet**	To be completed during and after completion of radiotherapy	**Form C**
**Post treatment form**	To be completed 30 days after last cycle of chemotherapy	**Form Post treatment**
**Follow-up forms**	To be completed at each follow-up visit 3 months after post cycle 4(6) visit	**Form FU**
**Serious Adverse Event form**	To be completed for all serious adverse events	**Form SAE**
**Prog/Rel/Death Form**	To be completed on death of patient.	**Form Prog/Rel/Death**

Essential Forms

**Pre randomisation**	**Pre treatment**	**Pre Cycle 1-6**	**Post Cycle 1-6**	**Post cycle 4 (6)**	**3, 6, 9, 12 months (then 6 monthly until death)**
Pre-a	Pre-b<br>Pre-c	A	B<br>C (2– BD)<br>C (4- OD)	Post treatment	FU

Additional Forms (if required)

**Any serious events**	**Progression**	**Relapse**	**Death**
SAE-SUSAR	Prog/Rel/Death	Prog/Rel/Death	Prog/Rel/Death

6.6 DATA HANDLING

A copy of all trial forms will be returned to the trials centre for statistical analysis. The remaining copy is to be retained at the local centre.

All forms will be scanned and entered into a study defined database for which some consistency checking will be programmed in. Data managers will check for missing and invalid data using SQL queries and statistical programs. Any queries will be highlighted on the forms and returned to the centres for correction. The data will at all times be password protected.
The CRF forms will be due as described in section 6.5.
On completion of the study the data will be written onto CD and archived in a safe and secure location within the department.
Paper copies of the CRF's will be retained on site for at least fifteen years following the last patient entered or if all are deceased may be archived off site. All paper data will be destroyed after fifteen years on the approval of the chief investigator.

The trials centre staff will be in regular contact with local centre personnel to check on progress and to help with any queries that may arise. Incoming forms will be checked for completeness, consistency, timeliness and compliance with the protocol. Centres may be withdrawn from further recruitment in the event of serious and persistent non-compliance.

7.0 STATISTICAL CONSIDERATIONS

7.1 Sample size calculation

It is considered that a survival benefit of 12% at 2 years (in favour of once-daily radiotherapy) would be clinically significant. Using Friedman's sample size calculation based on a 2-arm trial, with a 5% significance level, 2-sided test, 80% power, and hazard ratio of 0.70, a 12% overall survival benefit at 2 years from 44% with the control arm to 56% in the experimental arm, requires a total of 506 patients. An additional 5% will be added to allow for ineligible patients giving a total of 532 patients required.

7.1.1 Primary end-point

- Overall survival

Secondary end points

- Local progression-free survival
- Metastasis-free survival
- CTCAE v3.0 toxicity
- Chemotherapy dose intensity
- Radiotherapy dose intensity

7.2 General Considerations

For data analyses, patient data will be grouped by treatment arm according to the treatment assignments made via the Christie Hospital randomization line(described in Section 3.3).

7.2.1 Interim analyses

The only planned interim analyses will be performed for the independent Data Monitoring Committee (see section 9.0). A report will be given to the IDMC approximately 12 months after the first patient is randomised and then when requested by the IDMC.

7.3 Qualifications for Efficacy Analysis

7.3.1 Qualifications for Analysis of Time-to-Event and Other Efficacy

Parameters

The following are the qualifications for analysis of time-to-event efficacy parameters:

• All randomized patients will be included in the analysis of OS and Local PFS (ITT).

Overall survival and local progression-free survival.

Overall Survival is the time between date of randomisation and date of death of any cause. Survivors will be censored on the last date known to be alive. ***Local progression-free survival (local control)*** will

be calculated from the date of randomisation to the date of first clinical evidence of progressive disease at the primary site, or death.
Kaplan-Meier curves (Kaplan and Meier 1958) will be drawn for each treatment group. Overall survival and local progression-free survival will be compared using the Mantel-Cox version of the log rank test.

• All randomized patients treated with at least one study dose of cisplatin and etoposide will be included in the comparison of proportions of grade 3 and 4 toxicities.

Toxicity will be assessed according to NCI Common Terminology Criteria for Adverse Events v 3.0 (see appendix 2). The proportions of patients experiencing a grade of 3 or above acute toxicity, including acute radiation morbidity, or late radiation morbidity will be compared between the treatment groups using Chi-squared and Fisher Exact tests. Acute toxicity will be defined as toxicities occurring from commencement of treatment to 3 months after completion, late toxicity will be defined as toxicities occurring between 3 months and 2 years after completion of treatment.

7.3.2 Qualifications for Analysis of Tumour Response

All patients meeting the following criteria will be evaluated for tumour response and included in the analysis of tumour response rates:

• treatment with at least one dose of cisplatin and etoposide.

Response will be assessed according to RECIST criteria (appendix 4) and the proportion of patients in each treatment group whose best response up to, approximately 28 days post cycle 4 or, if stopped prior to cycle 4, approximately 28 days after last chemotherapy cycle given, from randomisation is complete or partial will be compared using Chi-squared and Fisher Exact tests.

7.4 Qualifications for Safety Analysis

Safety analyses will be performed for all randomized patients treated with at least one dose of study drug. Adverse effects will be summarized and compared between the two arms.

7.5 Qualifications for Dose Intensity Analysis

The relative chemotherapy and radiotherapy dose intensity will be summarised by calculating the median, standard deviation, interquartile range and range for patients in each randomised treatment group. The RDI will be compared between the treatment groups by using the Wilcoxon Rank Sum test.

Chemotherapy RDI:
Relative dose intensity (RDI) is defined as the dose intensity achieved by a patient relative to a schedule that would have given the intended PE chemotherapy on a fixed 3-weekly schedule. This is given a proportion of the fixed 3 weekly schedule and will be calculated assuming that each component of the chemotherapy schedule is equipotent. The duration of the chemotherapy for this calculation will run from the first day of chemotherapy to the last dose of the final cycle. The RDI will therefore be calculated as:

$$\frac{1}{2}\left(\frac{\text{Platinum}_{AT}}{\text{Platinum}_{PT}}+\frac{\text{Etoposide}_{AT}}{\text{Etoposide}_{PT}}\right)\cdot\left(\frac{\text{Time}_{PT}}{\text{Time}_{AT}}\right)$$

where Platinum_{AT}, Etoposide_{AT}, are the actual total received doses and Platinum_{PT}, Etoposide_{PT} are the respective planned total doses.

Radiotherapy RDI:
Relative dose intensity (RDI) is defined as the dose intensity achieved by a patient relative to a schedule that would have given the intended radiotherapy on a fixed 45 day schedule for OD and 19 day schedule for BD. The duration of the radiotherapy for this calculation will run from the first day of radiotherapy to the last day of the radiotherapy. The RDI will therefore be calculated as:

$$\left(\frac{totaldose_{AT}}{totaldose_{PT}}\right) \cdot \left(\frac{time_{PT}}{time_{AT}}\right)$$

where $totaldose_{AT}$, is the actual total dose received and $totaldose_{PT}$ is the respective planned total dose received.

7.6 Patient Disposition

A detailed description of patient disposition will include a summary of the following:
- all patients entered and enrolled: overall, by treatment arm, and by country
- reasons for patients entered, but not enrolled
- all enrolled patients treated with study drug, by treatment arm
- reasons patients enrolled, but not treated with study drug
- reasons patients discontinued study drug treatment
- all important protocol violations.

7.7 Patient Characteristics

Patient characteristics will include a summary of the following:
- patient demographics
- baseline disease characteristics
- number of chemotherapy cycles given

Other patient characteristics will be summarized as deemed appropriate.

7.8 Concomitant Therapy

Use of antibiotics and growth factors will be summarized for all randomized patients per treatment arm.

7.9 Subsequent Therapy

Subsequent treatment will be summarized for all randomized patients per treatment arm.

7.10 Treatment Compliance

Dose omissions, reductions, and delays will be summarized for all randomized patients per treatment arm.

7.11 Health Outcomes

These analyses will be performed in an exploratory manner and hence will not introduce multiplicity to primary and other secondary efficacy analyses.
- details of protocol medications
- other anti-cancer treatments
- management of serious adverse events
- hospital admission episodes

7.12 Stratification

The stratification factors include centre, performance status (0-1 versus 2), LDH, sodium, Alkaline Phosphatase. Comparison of data by centre will be scrutinised to identify any data inconsistencies, it will also be used to identify those centres planning to give four cycles of chemotherapy and those planning to give six, analysis will be carried out to identify any differences between to the two schedules. The other factors have been identified in previous studies to be prognostic factors and will be used to calculate the Manchester score which gives three groupings of good, intermediate and poor prognosis, these scores will be used to compare OS and response rate.

8.0 INFORMED CONSENT, ETHICAL & REGULATORY CONSIDERATIONS

8.1 ETHICAL APPROVAL

This trial will adhere to the principles outlined in the International Conference on Harmonisation Good Clinical Practice (ICH GCP) guidelines. It will be conducted in compliance with the protocol, MRC GCP, the Data Protection Act (DPA G0027154) and other regulatory requirements, as appropriate.

Multicentre Research Ethics Committee (MREC) approval has been obtained for this trial, and Site Specific Assessments (SSAs) will be performed at participating centres. The trials centre will maintain contact with NRES and will submit any protocol amendments. The trials centre will forward any resulting documentation to local centres.

8.2 PATIENT INFORMED CONSENT

The local investigator is required to explain the nature and purpose of the trial to the patient prior to trial entry. A detailed patient information sheet and consent form will be given to the patient and written informed consent obtained before trial entry.

8.3 PROTOCOL COMPLIANCE

Christie CTU office staff will be in regular contact with local centre personnel to check on progress and to help with any queries that may arise. Incoming forms will be checked for completeness, consistency, timeliness and compliance with the protocol. Centres may be withdrawn from further recruitment in the event of serious and persistent non-compliance.

8.4 INDEMNITY & COMPENSATION

The CONVERT trial is an investigator led and designed trial co-ordinated by the Christie Clinical Trials Unit and MRC Clinical Trials Unit (Cancer Division). The principal investigator, local investigators and co-ordinating centres do not hold insurance against claims for compensation for injury caused by participation in a clinical trial and they cannot offer any indemnity. The Association of the British Pharmaceutical Industry (ABPI) guidelines will not apply. However, in terms of liability, NHS Trust and Non-Trust Hospitals have a duty of care to patients treated, whether or not the patient is taking part in a clinical trial. Therefore compensation is available in the event of clinical negligence being proven.

8.5 DATA PROTECTION

The trials centre will act to preserve patient confidentiality and will not disclose or reproduce any information by which patients could be identified. Data will be stored in a secure manner and our trials are registered in accordance with the Data Protection Act 1998 with the Data Protection Officer at the Christie Hospital.

8.6 DURATION OF TRIAL

The 'active' phase of the trial will finish when the last patient has completed their protocol treatment. The planned duration of the 'active' phase is 4 years. The 'follow up' phase of the trial will then begin; patients will be followed until death.

8.7 PUBLICATION POLICY

Data from all centres will be analysed together and published as soon as possible. Individual participants may not publish data concerning their patients that are directly relevant to questions posed by the trial until the Trial Management Group (TMG) has published its report. The TMG will form the basis of the Writing Committee and advise on the nature of publications.

All publications shall include a list of participants, and if there are named authors, these should include the principal investigator, clinical trial coordinator(s), and statistician(s) involved in the trial and contributors of more than 5% of participants. If there are no named authors then a writing committee will be identified.

9.0 INDEPENDENT DATA MONITORING AND ETHICS COMMITTEE (IDMC), INDEPENDENT TRIAL STEERING COMMITTEE (TSC) AND TRIAL MANAGEMENT GROUP (TMG)

The data will be reviewed (at least annually) by an IDMC, consisting of at least two clinicians not entering patients into the trial and an independent statistician. The IDMC will be asked to recommend whether the accumulated data from the trial, together with results from other relevant trials, justifies continuing recruitment of further patients. A decision to discontinue recruitment, in all patients or in selected subgroups will be made only if the result is likely to convince a broad range of clinicians including participants in the trial and the general clinical community. If a decision is made to continue, the IDMC will advise on the frequency of future reviews of the data on the basis of accrual and event rates. The IDMC will make confidential recommendations to the TSC.

The role of the TSC is to act on behalf of the funder, to provide overall supervision for the trial, to ensure that it is conducted in accordance with GCP, and to provide advice through its independent Chairman. This independent committee will review the recommendations from the IDMC and will decide on continuing or stopping the trial, or modifying the protocol.

The Trial Management Group coordinates and manages the trial’s day-to-day activities. The TMG is comprised of health professionals as listed on page 3 of this protocol.

10.0 REFERENCES

1. Murray N, Coy P, Pater JL, et al.: Importance of timing for thoracic irradiation in the combined modality treatment of limited-stage small-cell lung cancer. Journal of Clinical Oncology 11(2): 336-344, 1993.
2. Johnson BE, Bridges JD, Sobczeck M, et al. Patients with limited-stage small-cell lung cancer treated with concurrent twice-daily chest radiotherapy and etoposide/cisplatin followed by cyclophosphamide, doxorubicin, and vincristine. Journal of Clinical Oncology 14(3): 806-813, 1996.
3. Turrisi AT III, Kim K, Blum R, et al.: Twice-daily compared with once-daily thoracic radiotherapy in limited small-cell lung cancer treated concurrently with cisplatin and etoposide. New England Journal of Medicine 340(4): 265-271, 1999.
4. Pignon JP, Arriagada R, Ihde DC, et al.: A meta-analysis of thoracic radiotherapy for small-cell lung cancer. N Engl J Med 1999, 340, 265-271
5. Warde P, Payne D: Does thoracic irradiation improve survival and local control in limited-stage small-cell carcinoma of the lung? A meta-analysis. J Clin Oncol 10(6): 890-895, 1992.
6. Thatcher N, Qian W, Clark P, et al. Ifosfamide, carboplatin, and etoposide with midcycle vincristine versus standard chemotherapy in patients with small-cell lung cancer and good performance status: clinical and quality-of-life results of the British Medical Research Council multicenter randomized LU21 trial. J Clin Oncol 2005; 23:8371-79
7. Takada M, Fukuoka M, Kawahara M, et al. Phase III Study of Concurrent Versus Sequential Thoracic Radiotherapy in Combination with Cisplatin and Etoposide for Limited-Stage Small Cell Lung Cancer : The Japan Clinical Oncology Group Study 9104. J Clin Oncol 20 (14): 3054-60, 2002.
8. Jeremic B, Shibamoto Y, Acimovic L et al. Initial versus delayed accelerated hyperfractionated radiation therapy and concurrent chemotherapy in limited small-cell lung cancer: a randomized study. J Clin Oncol 1997 15 (3): 893-900.
9. Gregor A, Drings P, Burghouts J et al.Randomised trial or alterning versus sequential radiotherapy/chemotherapy in limited disease patients with small cell lung cancer: a EORTC Lung Cancer Cooperative Group study. J Clin Oncol 1997; 15: 2840-49.
10. Work E, Nielson OS, Bentzen SM et al. Randomised study of initial versus late chest irradiation combined with chemotherapy in limited stage small cell lung cancer. J Clin Oncol 1997 15: 3030-37.
11. Fried B, Morris D, Hensing T et al. Systematic review evaluating the timing of thoracic radiation therapy in combined modality therapy for limited stage small-cell lung cancer. J Clin Oncol 2004, 22, 4785-93
12. Pijls-Johannesma MCG et al. Early versus late chest radiotherapy for limited stage small cell lung cancer. The Cochrane Database of Systematic Reviews; Issue 4. Art. No: CD004700, 2004.
13. Carney DN, Mitchel BJ, Kinsella TJ. In vitro radiation and chemotherapy sensitivity of established cell lines of human small cell lung cancer and its large cell morphologic variants. Cancer Research 1983 43: 2806-11
14. Bonner JA, Sloan JA, Shanahan TG, Brooks BJ, Marks RS, Krook JE, Gerstner JB, Maksymiuk A, Levitt R, Mailliard JA, Tazelaar HD, Hillman S and Jett JR. Phase III comparison of twice-daily split-course irradiation versus once-daily irradiation for patients with limited stage small-cell lung carcinoma. J Clin Oncol 1999, 17, 2681-2691
15. Schild SE et al. Long-term results of a phase III trial comparing once-daily radiotherapy with twice-daily radiotherapy in limited-stage small-cell lung cancer. Int J Radiat Oncol Biol Phys 2004, 59, 943-51
16. Choi NC, Herndon III JE, Rosenman J, Carey RW, Chung CT, Bernard S, Leone L, Seagren S, Green M. Phase I study to determine the maximum-tolerated dose of radiation in standard daily and hyperfractionated-accelerated twice-daily radiation schedules with concurrent chemotherapy for limited-stage small-cell lung cancer. J Clin Oncol 1998, 16, 3528-3537
17. Komaki R, Swann RS, Ettinger DS, et al. Phase I study of thoracic radiation dose escalation with concurrent chemotherapy for patients with limited small-cell lung cancer: Report of Radiation Therapy Oncology Int J Radiat Oncol Biol Phys 2005 ;62(2):342-50
18. Mascaux C, Paesmans M, Berghmans T, Branle F, Lafitte JJ, Lemaitre F, Meert AP, Vermylen P and Sculier JP. A systematic review of the role of etoposide and cisplatin in the chemotherapy of small cell lung cancer with methodology assessment and meta-analysis. Lung Cancer 2000, 30, 23-36
19. Pujol J-L, Carestia L and Daures J-P. Is there a case for cisplatin in the treatment of small-cell lung cancer? A meta-analysis of randomised trials of a cisplatin-containing regimen versus a regimen without this alkylating agent. Br J Cancer 2000, 83, 8-15

20. Sundstrom S. Bremnes RM. Kaasa S. Aasebo U. Hatlevoll R. Dahle R. Boye N. Wang M. Vigander T. Vilsvik J. Skovlund E. Hannisdal E. Aamdal S. Norwegian Lung Cancer Study Group. Cisplatin and etoposide regimen is superior to cyclophosphamide, epirubicin, and vincristine regimen in small-cell lung cancer: results from a randomized phase III trial with 5 years' follow-up. J Clin Oncol 2002, 20, 4665-4672
21. Bremnes RM, Sundstrom S, Vilsvik J, Aasebo U; Norwegian Lung Cancer Study Group. Multicenter phase II trial of paclitaxel, cisplatin, and etoposide with concurrent radiation for limited-stage small-cell lung cancer. J Clin Oncol 2001, 19, 3532-3538
22. Thatcher N, Girling DJ, Hopwood P, Sambrook RJ, Qian W, Stephens RJ. Improving survival without reducing quality of life in small-cell lung cancer patients by increasing the dose-intensity of chemotherapy with granulocyte colony-stimulating factor support: results of a British Medical Research Council Multicenter Randomized Trial. Medical Research Council Lung Cancer Working Party. J Clin Oncol 2000, 18, 395-404
Pujol JL, Douillard JY, Riviere A, Quoix E, Lagrange JL, Berthaud P, Bardonnet-Comte M, Polin V, Gautier V, Milleron B, Chomy F, Chomy P, Spaeth D, Le Chevalier T. Dose-intensity of a four-drug chemotherapy regimen with or without recombinant human granulocyte-macrophage colony-stimulating factor in extensive-stage small-cell lung cancer: a multicenter randomized phase III study. J Clin Oncol 1997, 15, 2082-2089
23. Johnson DH, Carbone DP. Increased dose-intensity in small-cell lung cancer: a failed strategy? J Clin Oncol 1999, 17, 2297-2299
24. Laurie SA, Logan D, Markman BR, Mackay JA, Evans WK. Practice guidelines for the role of combination chemotherapy in the initial management of limited-stage small cell lung cancer. Lung Cancer 2004, 43, 223-240
25. Senan, S., et al., Literature-based recommendations for treatment planning and execution in high-dose radiotherapy for lung cancer. Radiother Oncol, 2004. 71(2): 139-46.
26. ICRU (The International Commission on Radiation Units and Measurements) Report 50: Prescribing, Recording and Reporting Photon Beam Therapy. Landberg T, Chavandra J, Dobbs HJ, Hanks G, Johansson KA, Muller R, Purdy J. 1993. ICRU Publications, Bethesda.
27. Royston P, and Babiker A. A menu-driven facility for complex sample size calculation in randomized controlled trials with a survival or a binary outcome. Stata Journal 2002, 2, 151-163
28. Perry MC, Eaton WL, Propert KJ, et al. Chemotherapy with or without radiation therapy in limited small-cell carcinoma of the lung. N Engl J Med. 1987 Apr 9;316(15):912-8
29. Pijls-Johannesma MCG et al. Early versus late chest radiotherapy for limited stage small cell lung cancer. The Cochrane Database of Systematic Reviews; Issue 4. Art. No: CD004700, 2004.

11.0 GLOSSARY

**ADL**	Activities of Daily Living
**AE**	Adverse event
**ALT**	Alanine transaminase
**ANC**	Absolute Neutrophil Count
**AR**	Adverse reaction
**AST**	Aspartate aminotransferase
**ASTRO**	American Society for Therapeutic Radiology and Oncology
**AUC**	Area Under the Curve
**BD**	Twice daily
**CI**	Chief Investigator
**CR**	Complete Response
**CRF**	Case Report Form
**CT**	Computed Tomography
**CTU**	Clinical Trial Unit
**CXR**	Chest X-Ray
**D**	Day
**DLCO**	Gas Transfer Factor for Carbon Monoxide (Lung diffusion test)
**DPA**	Data Protection Act
**DVH**	Dose Volume Histograms
**ECG**	Electrocardiogram
**ECOG**	Eastern Cooperative Oncology Group
**EDTA**	Ethylene Diamine Tetra Acetate
**ESTRO**	European Society for Therapeutic Radiology and Oncology
**FBC**	Full Blood Count
**FEV1**	Forced Expiratory Volume in 1 second
**GCSF**	Granulocyte Colony Stimulating Factor
**GFR**	Glomerular Filtration Rate
**γGT**	Gamma Glutamiyl –Transferase
**GTV**	Gross Tumour Volume
**Gy**	Gray (unit of absorbed radiation dose)
**Hb**	Haemoglobin
**ICH GCP**	International Conference of Harmonisation - Good Clinical Practice
**ICRU**	The International Commission on Radiation Units and Measurements
**IDMC**	Independent Data Monitoring and Ethics Committee
**IMP**	Investigational Medicinal Product
**IPEM**	Institute of Physics and Engineering in Medicine
**IR(ME)R**	Ionising Radiations (Medical Exposure) Regulations
**ITT**	Intention-to-treat
**iv**	Intravenous
**KCL**	Potassium Chloride
**LD**	Longest Diameter
**LDH**	Lactic Dehydrogenase
**LFT**	Liver Function Tests
**LS**	Limited stage
**M**	Distant metastasis
**mmols**	Millimoles
**mmols/L**	Millimoles per litre
**μmol/L**	Micromoles per litre
**MHRA**	Medicines and Healthcare Related products Agency
**MRC**	Medical Research Council
**MV**	Megavolts
**N**	Nodal Involvement
**NCI CTCAE**	National Cancer Institute Common Terminology Criteria for Adverse Events
**NHS**	National Health Service

**NRES**	National Research Ethics Service
**OS**	Overall Survival
**PA**	Posteroanterior
**PCI**	Prophylactic cranial irradiation
**PD**	Progressive Disease
**PET**	Positron Emission Tomography
**PFS**	Progression-Free Survival
**PI**	Principal Investigator (named clinician at each centre)
**PO**	By mouth
**PR**	Partial Response
**PS**	Performance Status
**PTV**	Planning Target Volume
**PE**	Cisplatin and etoposide
**QL**	Quality of Life
**QALYS**	Quality-Adjusted Life-Years
**QART**	Quality Assurance in Radiotherapy
**REC**	Research Ethics Committee
**RECIST**	Response Evaluation Criteria in solid Tumours
**RT (#)**	Radiotherapy (fractions)
**SAE**	Severe Adverse Event
**SCLC**	Small cell lung cancer
**SD**	Stable Disease
**SmPC**	Summary of product characteristics
**SSA**	Site Specific Assessment
**SUSAR**	Suspected Unexpected Serious Adverse Events
**T**	Primary Tumour
**TD**	Total Dose
**TMG**	Trial Management Group
**TSC**	Trial Steering Committee
**U+E**	Urea + Electrolytes
**ULN**	Upper Limit of Normal
**V_{20} lung**	Volume of lung tissue receiving $\geq$ 20 Gy

APPENDIX 1

PERFORMANCE STATUS SCALE (ECOG)

GRADE

0 - Fully active, able to carry on all predisease performance without restriction (Karnofsky 90-100).

1 - Restricted in physically strenuous activity but ambulatory and able to carry out work of a light or sedentary nature, e.g. light house work, office work (Karnofsky 70-80).

2 - Ambulatory and capable of all self-care but unable to carry out any work activities. Up and about more than 50% of waking hours (Karnofsky 50-60).

3 - Capable of only limited self-care, confined to bed or chair more than 50% of waking hours (Karnofsky 30-40).

4 - Completely disabled. Cannot carry on any self-care. Totally confined to bed or chair (Karnofsky 10-20).

ref: - Oken, M.M., Creech, R.H., Tormey, D.C., Horton, J., Davis, T.E., McFadden, E.T., Carbone, P.P.: Toxicity And Response Criteria Of The Eastern Cooperative Oncology Group. Am J Clin Oncol 5:649-655, 1982.

ASSESSMENT OF PERFORMANCE STATUS (PS)

Performance Status (PS) is a critical prognostic factor in small cell lung cancer and an essential guide for therapy. Measurement of PS is an evaluation of the patient's activity levels at a specific point in time, and should not be influenced by either the patient's self perception based on previous levels of activity or subjective impressions of the patient's general appearance by the assessor. Specific questions by the clinician should include enquiry about how much time the patient has spent sitting or lying down during daylight hours in the past week (this can be expressed as a percentage or fraction of the day) and the most strenuous activity undertaken by the patient in the past few days. A list of activities performed in the previous three days can be useful. If the clinician is uncertain as to the patient's exact PS, then the patient should be asked to keep an activity diary for seven days and reassessed a week later.

APPENDIX 2

Selected NCI Common Terminology Criteria for Adverse Events v3.0 (revised 2003)

Full CTCAE available at http://ctep.cancer.gov (under Reporting Guidelines)

		Grade				
**Adverse Event**	**Short name**	**1**	**2**	**3**	**4**	**5**
**Allergy**						
Allergic reaction/ Hypersensitivty (including drug fever)	Allergic reaction	Transient flushing or rash, drug fever $< 38\ ^{o}C$	Rash, flushing urticaria, dyspnoea, drug fever $\geq 38\ ^{o}C$	Symptomatic bronchospasm, with or without urticaria; parenteral medication(s) indicated; allergy -related edema/angioedema; or hypotension	Anaphylaxis	Death
Drug fever should be coded under allergy/hypersensitivity. If fever is due to infection, code infection only						
**Blood/bone marrow**						
Haemoglobin (g/dl)	Haemoglobin	10 - <LLN	8.0 - 9.9	6.5 - 7.9	<6.5	Death
Neutrophils/ granulocytes ($x\ 10^{9}/L$)	Neutrophils	1.5 - <LLN	1.0 - 1.4	0.5 - 0.9	<0.5	Death
Leucocytes (total WBC) ($x\ 10^{9}/L$)	Leucocytes	3.0 - <LLN	2.0 - 2.9	1.0 - 1.9	<1.0	Death
Platelets - ($x\ 10^{9}/L$)	Platelets	75.0 - <LLN	50.0 - 74.9	25 - 49.9	<25.0	Death
**Cardiac**						
Cardiac General – Other (Specify)	Cardiac General – Other (Specify)	Mild	Moderate	Severe	Life threatening; disabling	Death
**Constitutional symptoms**						
Fatigue (asthenia, lethargy, malaise)	Fatigue	Mild fatigue over baseline	Moderate, some difficulties with ADL	Severe, interfering with ADL	Disabling	-
Fever (in absence of neutropenia ANC <1.0 x $10^{9}/L$)	Fever	$38.0 – 39.0\ ^{0}C$	$>39.0 – 40.0\ ^{0}C$	$> 40.0\ ^{0}C$ for $\leq$ 24 hours	$> 40.0\ ^{0}C$ for > 24 hrs	Death
Fever felt to be caused by a drug should be coded as allergic reaction/hypersensitivity (including drug fever). If fever is due to infection, code infection only						

		Grade				
**Adverse Event**	**Short name**	**1**	**2**	**3**	**4**	**5**
Rigors/chills	Rigors/chills	Mild	Moderate, narcotics indicated	Severe or prolonged, not responsive to narcotics	-	-
**Dermatological/skin**						
Dry Skin	Dry Skin	Asymptomatic	Symptomatic, not interfering with ADL	Interfering with ADL	-	-
Hair loss/ Alopecia (scalp or body)	Alopecia	Thinning or patchy	Complete	-	-	-
Pruritus/itching	Pruritus/itching	Mild or localised	Intense or widespread	Intense or widespread and interfering with ADL	-	-
Rash/desquamation	Rash	Macular or papular eruption or erythema without associated symptoms	Macular or papular eruption or erythema with pruritis or other associated symptoms; localised desquamation or other lesions covering <50% of body surface area (BSA)	Severed, generalised erythroderma or macular, papular or vesicular eruption, desquamation covering ≥50% BSA	Generalised exfoliative, ulcerative, or bullous dermatitis	Death
Rash:<br>Dermatitis associated with radiation<br>*- Select:*<br>- Chemoradiation<br>- Radiation	Dermatitis<br>*- Select*	Faint erythema or dry desquamation	Moderate to brisk erythema; patchy moist desquamation, mostly confined to skin folds and creases; moderate oedema	Moist desquamation other than skin folds and creases; bleeding induced by minor trauma or abrasion	Skin necrosis or ulceration of full thickness dermis; spontaneous bleeding from involved site	Death
Rash:<br>Erythema multiforme (e.g. Stevens-Johnson syndrome, toxic epidermal necrolysis)	Erythema multiforme	-	Scattered, but not generalised eruption	Severe (e.g. generalised rash or painful stomatitis); IV fluids, tube feedings or TPN indicated	Life threatening, disabling	Death
Please note:	*Rash due to allergy is coded under Allergic reaction/ Hypersensitivty (including drug fever).*<br>*Rashes due to radiation, erythema multiforme, or the hand-foot skin reaction are coded separately. See full CTCAE*					
Dermatology/Skin<br>- Other (Specify)	Dermatology/Skin<br>- Other (Specify)	Mild	Moderate	Severe	Life threatening; disabling	Death

		Grade				
**Adverse Event**	**Short name**	**1**	**2**	**3**	**4**	**5**
**Gastro-Intestinal**						
Anorexia	Anorexia	Loss of appetite without alteration in eating habits	Oral intake altered without significant weight loss or malnutrition; oral nutritional supplements indicated	Associated with significant weight loss/malnutrition; IV fluids, tube feeding or TPN indicated	Life threatening consequences	Death
Constipation	Constipation	Occasional or intermittent symptoms; occasional use of stool softeners, laxatives, dietary modification, or enema	Persistent symptoms with regular use of laxatives or enemas indicated	Symptoms interfering with ADL; obstipation with manual evacuation indicated	Life-threatening consequences (eg obstruction, toxic megacolon)	Death
Diarrhoea	Diarrhoea	Increase of <4 stools/day over baseline; mild increase in ostomy output compared to baseline	Increase of 4-6 stools/day over baseline; IV fluids indicated < 24 hrs; moderate increase in ostomy output compared to baseline; not interfering with ADL	Increase of ≥ 7 stools/day over baseline; incontinence; IV fluids or parenteral support ≥ 24hrs; hospitalization; severe increase in ostomy output compared to baseline; interfering with ADL	Life-threatening consequences e.g. haemodynamic collapse	Death
Ileus, GI (functional obstruction of bowel, i.e. neuroconstipation)	Ileus	Asymptomatic, radiographic findings only	Symptomatic; altered GI function (e.g. altered dietary habits); IV fluids indicated <24hrs	Symptomatic and severely altered GI function; IV fluids, tube feeding, or TPN indicated ≥ 24hrs	Life-threatening consequences	Death
Mucositis/Stomatitis/ (clinical exam)<br><br>- *Select:*<br>- Anus<br>- Oesophagus<br>- Etc	Mucositis (clinical exam)<br>– *Select*	Erythema of the mucosa	Patchy ulceration or pseudomembranes	Confluent ulceration/pseudomebranes, or bleeding with minor trauma	Tissue necrosis, significant spontaneous bleeding, or life threatening consequences	Death

		Grade				
**Adverse Event**	**Short name**	**1**	**2**	**3**	**4**	**5**
Nausea	Nausea	Loss of appetite without alteration in eating habits	Oral intake decreased without signs of weight loss/dehydration, malnutrition; IV fluids indicated < 24 hrs	Inadequate oral caloric or fluid intake; IV fluids, tube feeding , or TPN indicated ≥ 24 hrs	Life threatening consequences	Death
Oesophagitis	Oesophagitis	Asymptomatic pathologic, radiographic, or endoscopic findings only	Symptomatic; altered eating/swallowing (e.g. altered dietary habits, oral supplements); IV fluids indicate < 24 hrs	Symptomatic and severely altered eating/swallowing (e.g. indadequate oral intake); IV fluids tube feedings, or TPN indicated ≥ 24 hrs	Life-threatening consequences	Death
Vomiting	Vomiting	1 episode in 24 hrs	2-5 episodes in 24 hrs. IV fluids for <24 hrs	≥ 6 episodes in 24 hrs or IV fluids or TPN ≥ 24 hrs	Life threatening consequences	Death
**Infection**						
Febrile neutropenia (fever of unknown origin without clinically or microbiologically documented infection (ANC <1.0 x 10^9L, fever ≥ 38.5 ^{0}C)	Febrile neutropenia	-	-	Present	Life threatening consequences eg septic shock, hypotension, acidosis, necrosis	Death
Infection (documented clinically or microbiologically) with Grade 3 or 4 neutrophils (ANC <1.0 x 10^9L) *- Select*	Infection (documented clinically) *- Select*	-	Localized, local intervention indicated	IV antibiotic, antifungal or antiviral interventional or operative intervention	Life threatening consequences eg septic shock, hypotension, acidosis necrosis	Death
Infection with normal ANC or Grade 1 or 2 neutrophils *- Select*	Infection with normal ANC *- Select*	-	Localized, local intervention indicated	IV antibiotic, antifungal or antiviral intervention or operati intervention	Life threatening consequences eg septic shock, hypotension, acidosis necrosis	Death
Please refer to the end of the CATEGORY on the full CTCAE ver 3.0 for list of AEs to select						

		Grade				
**Adverse Event**	**Short name**	**1**	**2**	**3**	**4**	**5**
**Muscolosketetal/soft tissue**						
Muscolosketetal/soft tissue - Other (Specify)	Muscolosketetal - Other (Specify)	Mild	Moderate	Severe	Life Threatening, Disabling	Death
**Neurological**						
Dizziness	Dizziness	With head movements or nystagmus only, not interfering with function	Interfering with function, but not with ADL	Interfering with ADL	Disabling	-
Myelitis	Myelitis	Asymptomatic, mild signs eg Babinski's or L'Hermitte's sign	Weakenss or sensory loss not interfering with ADL	Weakenss or sensory loss interfering with ADL	Disabling	Death
Neuropathy: sensory	Neuropathy: sensory	Asymptomatic; loss of deep tendon reflexes or parasthesia (including tingling) not interfering with function	Sensory alteration of paresthesia (including tingling), interfering with function, but not interfering but not ADL	Sensory alteration or parasthesia interfering with ADL	Disabling	Death
Somnolence/ Depressed level of consciousness	Somnolence	-	Somnolence or sedation interfering with function, but not with ADL	Obtundation or stupor, difficult to arouse, interfering with ADL	Coma	Death
**Pain**-*select*						
Pain<br>- *Select*	- Pulmonary/ Upper respiratory<br>- *Select*<br>- Chest wall<br>- Chest/thorax NOS	Mild pain not interfering with function	Moderate pain; pain or analgesics interfering with function, but not interfering with ADL	Severe pain; pain or analgesics severely interfering with ADL	Disabling	-

		Grade				
**Adverse Event**	**Short name**	**1**	**2**	**3**	**4**	**5**
**Pulmonary**						
Pneumonitis/pulmonary infiltrates	Pneumonitis	Asymptomatic, radiographic findings only	Symptomatic, not interfering with ADL	Symptomatic, interfering with ADL; O_2 indicated	Life-threatening; ventilatory support indicated	Death
Pneumonitis fibrosis	Pneumonitis fibrosis	Minimal radiographic findings (or patchy bibasilar changes) with estimated radiographic proportion of the total lung volume that is fibrotic of <25 %	Patchy or bi-basilar changes with estimated radiographic proportion of the total lung volume that is fibrotic of 25-<50 %	Dense or widespread infiltrates/consolidation with estimated radiographic proportion of the total lung volume that is fibrotic of 50 -<75 %	Estimated radiographic proportion of the total lung volume that is fibrotic is ≥ 75 %; honeycombing	Death
**Renal**						
Renal/Genitourinary<br>- Other (Specify)	Renal<br>- Other (Specify)	Mild	Moderate	Severe	Disabling, life threatening	Death
Other – Specify - eg Creatinine: Mild >- 1.5 x ULN ; Moderate >1.5 – 3.0 x ULN; Severe >3.0 – 6.0 x ULN; Disabling >6.0 x ULN						
**Vascular**						
Thrombosis/thrombus/ embolism	Thrombosis/thrombus/ embolism	-	Deep vein thrombosis or cardiac thrombosis; intervention (e.g. anticoagulation, lysis) not indicated	Deep vein thrombosis, or cardiac thrombosis; intervention (e.g. anticoagulation, lysis) indicated	Embolic event including pulmonary embolism or life-threatening thrombus	Death

APPENDIX 3

MRC DYSPNOEA SCORE

GRADE

0 - Climbs hills or stairs without dyspnoea

1 - Walks any distance on flat without dyspnoea

2 - Walks over 100 yards without dyspnoea

3 - Dyspnoea on walking 100 yards or less

4 - Dyspnoea on mild exertion, eg undressing

Ref: MRC Lung Cancer Working Party. 'A randomised trial of three or six courses of etoposide cyclophosphamide methotrexate and vincristine or six courses of etoposide and ifosfamide in small cell lung cancer (SCLC) 1: survival and prognostic factors. (1993) BJC 68, 1150-56

APPENDIX 4

RESPONSE CRITERIA (RECIST)

Baseline documentation of "Target" and "Non-Target" lesions

- All measurable lesions up to a maximum of five lesions per organ and 10 lesions in total, representative of all involved organs should be identified as ***target lesions*** and recorded and measured at baseline.
- Target lesions should be selected on the basis of their size (lesions with the longest diameter) and their suitability for accurate repeated measurements (either by imaging techniques or clinically).
- A sum of the longest diameter (LD) for *all target lesions* will be calculated and reported as the baseline sum LD. The baseline sum LD will be used as reference by which to characterize the objective tumor.
- All other lesions (or sites of disease) should be identified as ***non-target lesions*** and should also be recorded at baseline. Measurements of these lesions are not required, but the presence or absence of each should be noted throughout follow-up.

Response Criteria

	**Evaluation of target lesions**
**Complete Response (CR):**	Disappearance of all target lesions
**Partial Response (PR):**	At least a 30% decrease in the sum of the LD of target lesions, taking as reference the baseline sum LD
**Progressive Disease (PD):**	At least a 20% increase in the sum of the LD of target lesions, taking as reference the smallest sum LD recorded since the treatment started or the appearance of one or more new lesions
**Stable Disease (SD):**	Neither sufficient shrinkage to qualify for PR nor sufficient increase to qualify for PD, taking as reference the smallest sum LD since the treatment started
	**Evaluation of non-target lesions**
**Complete Response (CR):**	Disappearance of all non-target lesions and normalization of tumor marker level
**Incomplete Response/ Stable Disease (SD):**	Persistence of one or more non-target lesion(s) or/and maintenance of tumor marker level above the normal limits
**Progressive Disease (PD):**	Appearance of one or more new lesions and/or unequivocal progression of existing non-target lesions (1)

(1) Although a clear progression of "non target" lesions only is exceptional, in such circumstances, the opinion of the treating physician should prevail and the progression status should be confirmed later on by the review panel (or study chair).

Evaluation of best overall response

The best overall response is the best response recorded from the start of the treatment until disease progression/recurrence (taking as reference for PD the smallest measurements recorded since the treatment started). In general, the patient's best response assignment will depend on the achievement of both measurement and confirmation criteria

Target lesions	Non-Target lesions	Evaluation of non-target lesions	Overall response
CR	CR	No	CR
CR	Incomplete response/SD	No	PR
PR	Non-PD	No	PR
SD	Non-PD	No	SD
PD	Any	Yes or No	PD
Any	PD	Yes or No	PD
Any	Any	Yes	PD

- Patients with a global deterioration of health status requiring discontinuation of treatment without objective evidence of disease progression at that time should be classified as having "symptomatic deterioration". Every effort should be made to document the objective progression even after discontinuation of treatment.
- In some circumstances it may be difficult to distinguish residual disease from normal tissue. When the evaluation of complete response depends on this determination, it is recommended that the residual lesion be investigated (fine needle aspirate/biopsy) to confirm the complete response status.

Ref: Therasse et al 'New guidelines to evaluate the response to treatment in solid tumours' (2000) JNCI. 92 (3); 205-16

A quick reference guide is given at: **www3.cancer.gov/bip/RECIST.htm**

APPENDIX 5

COCKCROFT & GAULT FORMULA

If creatinine measured in μmol/l:

Males:

$$\frac{1.23 \times (140 - \text{age}) \times \text{weight(kg)}}{\text{serum creatinine } (\mu\text{mol/l})}$$

Females:

$$\frac{1.05 \times (140 - \text{age}) \times \text{weight(kg)}}{\text{serum creatinine } (\mu\text{mol/l})}$$

If creatinine measured in mg/%

Males:

$$\frac{(140 - \text{age}) \times \text{wt (kg)}}{72 \times \text{serum creatinine}}$$

Females:

$$\frac{(140 - \text{age}) \times \text{wt (kg)} \times 0.85}{72 \times \text{serum creatinine}}$$

APPENDIX 6

SUGGESTED SCHEME FOR ADMINISTRATION OF CHEMOTHERAPY CYCLES

Etoposide and Cisplatin every 21 days for 4-6 cycles

Etoposide

100 mg/m^2 days 1-3 BSA daily administered as an intravenous infusion over 1 hour every 3 weeks

Cisplatin

75 mg/m^2 day 1 or 25 mg/m^2 days 1-3 BSA administered as an intravenous infusion over 2 hours every 3 weeks

OPTION 1: Cisplatin given on day 1 only
Day 1

Administer anti-emetics according to local guidelines

Time	Drug	Fluid
0.00	-	1 litre 0.9% sodium chloride over 2 hours with 20 mmol of KCl
When urine output $\geq$ 100ml/hour:		
2.00	cisplatin 75mg/m^2	Given in 1 litre normal saline with 1g MgCl 20mmol over 2 hours
4.00	Etoposide 100mg/m^2	1 litre 0.9% sodium chloride over 1 hour
5.30	Post – cisplatin hydration	1 litre iv normal saline with KCl 20mmol and magnesium sulphate 10 mmol over 2 hours

Maintain oral intake of 1-2 litres of fluid for 6 hours after iv fluids discontinued.

Days 2 and 3

Administer anti-emetics according to local guidelines.

0.00	Etoposide 100 mg/m^2	1 litre 0.9% sodium chloride over 1 hour

OPTION 2: Cisplatin given day 1 to 3

Days 1-3

Time	Drug	Fluid
0.00	Cisplatin 25 mg/m2 d1-3	500ml normal saline over 1 hour
1.00	Etoposide 100 mg/m2 d1-3	500ml normal saline over 1 hour

Patients to have an oral intake of 2 litres of fluid days 1 to 3

APPENDIX 7

CHEMOTHERAPY AGENTS - safety profiles

Etoposide - http://emc.medicines.org.uk (as per SmPC)

Safety profile
The main toxicities associated with etoposide are:

Gastrointestinal toxicity: Nausea and vomiting are the major gastro-intestinal toxicities. The severity of such nausea and vomiting is generally mild to moderate with treatment discontinuation required in 1% of patients. Diarrhoea, anorexia and mucositis may occur. Constipation and swallowing disorder have been observed rarely.

Haematoxicity: Myelosuppression is dose limiting, with granulocyte nadirs occurring 5 to 15 days after drug administration and platelet nadirs occurring 9 to 16 days after drug administration. Bone marrow recovery is usually complete by day 21, and no cumulative toxicity has been reported.

Allergic reactions: Seldom hypersensitivity reactions caused by benzyl alcohol may occur. Anaphylactic-like reactions characterised by flushing, tachycardia, bronchospasm, and hypotension have been reported (incidence 0.7-2%), also apnoea followed by spontaneous recurrence of breathing after withdrawal of etoposide infusion, increase in blood pressure. The reactions can be managed by cessation of the infusion and administration of pressor agents, corticosteroids, antihistamines and/or volume expanders as appropriate.

Hypotension: Transient hypotension following rapid intravenous administration has been reported in 1% to 2% of patients. It has not been associated with cardiac toxicity or electrocardiographic changes. To prevent this rare occurrence, it is recommended that etoposide is administered by slow intravenous infusion over a 30- to 60-minute period. If hypotension occurs, it usually responds to supportive therapy after cessation of the administration. When restarting the infusion, a slower administration rate should be used.

Other toxicities: Reversible alopecia, sometimes progressing to total baldness was observed in up to 70% of patients. The following adverse reactions have been infrequently reported:
peripheral neuropathy, paresthesia, increased liver function tests with high doses, radiation "recall" dermatitis, hand-foot syndrome, central nervous effects (fatigue, drowsiness) 0-3%, hyperuricemia, taste impairment, fever, rash, urticaria, skin discoloration, pruritus, abdominal pain. The following adverse events have been reported after administration of etoposide (a causal relationship has not been established): Stevens-Johnson syndrome, rhythm disorders, myocardial infarction, reversible loss of vision.

Cisplatin - http://emc.medicines.org.uk (as per SmPC)

Safety profile
The main toxicities associated with cisplatin are:

Nephrotoxicity: Renal toxicity has been noted in about one third of patients given a single dose of cisplatin when prior hydration has not been employed. It is first noted during the second week after a dose and is manifested by elevations in plasma urea and serum creatinine, serum uric acid and/or decrease in creatinine clearance.

Renal toxicity becomes more prolonged and severe with repeated courses of the drug. Renal function must return to acceptable levels before another dose of cisplatin can be given.

Renal function impairment has been associated with renal tubular damage. The administration of cisplatin using a 6-8 hour infusion with intravenous hydration and mannitol has been used to reduce nephrotoxicity. However renal toxicity still can occur after utilisation of these procedures.

Gastrointestinal toxicity: Nausea and vomiting occur in the majority of patients, usually starting within 1 hour of treatment and lasting up to 24 hours. Anorexia, nausea and occasional vomiting may persist for up to a week.

Ocular toxicity: There have been reports of optic neuritis, papilloedema and cerebral blindness following treatment with cisplatin. Improvement and/or total recovery usually occurs following immediate discontinuation. Blurred vision and altered colour perception have been reported after the use of regimens with higher doses of cisplatin or greater dose frequencies than those recommended.

Ototoxicity: Ototoxicity has occurred in up to 31% of patients treated with a single dose of cisplatin 50 mg/m^2. Ototoxicity may be more severe in children and more frequent and severe with repeated doses.

Careful monitoring should be performed prior to initiation of therapy and prior to subsequent doses of cisplatin.

Unilateral or bilateral tinnitus, which is usually reversible, and/or hearing loss in the high frequency range may occur.

The overall incidence of audiogram abnormalities is 24%, but large variations exist. These abnormalities usually appear within 4 days after drug administration and consist of at least a 15 decibel loss in pure tone threshold. The damage seems to be cumulative and is not reversible. The audiogram abnormalities are most common in the 4000-8000 Hz frequencies.

Haematoxicity: Myelosuppression is observed in about 30% of patients treated with cisplatin. Leucopenia and thrombocytopenia are more pronounced at higher doses. The nadirs in circulating platelets and leucocytes generally occur between days 18-23 (range 7.3 to 45) with most patients recovering by day 39 (range 13 to 62). Leucopenia and thrombocytopenia are more pronounced at doses greater than 50 mg/m^2. Anaemia (decreases of greater than 2 g% haemoglobin) occurs at approximately the same frequency, but generally with a later onset than leucopenia and thrombocytopenia. Subsequent courses of cisplatin should not be instituted until platelets are present at levels greater than 100,000/mm^2 and white cells greater than 4,000/mm^2. A high incidence of severe anaemia (range 9-40%) requiring transfusion of packed red cells has been observed in patients receiving combination chemotherapy including cisplatin.

Anaphylaxis: Reactions possibly secondary to cisplatin therapy have been occasionally reported in patients who were previously exposed to cisplatin. Patients who are particularly at risk are those with a prior history or family history of atopy. Facial oedema, wheezing, tachycardia, hypotension and skin rashes of urticarial non-specific maculopapular type can occur within a few minutes of administration. Serious reactions seem to be controlled by IV adrenaline, corticosteroids or antihistamines.

Serum electrolyte disturbances: Hypomagnesemia, hypocalcemia, hyponatremia, hypokalemia and hypophosphatemia have been reported to occur in patients treated with cisplatin and are probably related to renal tubular damage. Hypomagnesaemia and hypocalcaemia may result in tetany. Generally, normal serum electrolyte levels are restored by administering supplemental electrolytes and discontinuing cisplatin. Inappropriate antidiuretic hormone syndrome has also been reported.

Neurotoxicity: Usually characterised by peripheral neuropathies and paresthesia in both upper and lower extremities. Peripheral neuropathy, while reversible, may take a year or more to recover. Loss of taste and seizures have also been reported. Neuropathies resulting from cisplatin treatment may occur after prolonged therapy; however, neurological symptoms have been reported to occur after a single dose. The neuropathy may progress after stopping treatment.

Hyperuricemia: Hyperuricemia occurring with cisplatin is more pronounced with doses greater than 50 mg/m^2. Allopurinol effectively reduces uric acid levels.

Other toxicities: Vascular toxicities coincident with the use of cisplatin in combination with other antineoplastic agents have been reported rarely. These events may include myocardial infarction, cerebrovascular accident, thrombotic microangiopathy (haemolytic uraemic syndrome) or cerebral arteritis. There are also reports of Raynaud's phenomenon occurring in patients treated with the combination of bleomycin, vinblastine with or without cisplatin. It has been suggested that hypomagnesaemia developing coincident with the use of cisplatin may be an added, although not essential factor, associated with this event. However the cause of this Raynaud's phenomenon is currently unknown.

Other toxicities reported to occur infrequently are cardiac abnormalities including tachycardia, postural hypotension and arrhythmia.

Local soft tissue toxicity has been reported rarely following extravasation of cisplatin. Infiltration of solutions of cisplatin may result in tissue cellulitis, fibrosis and necrosis.

Carboplatin - http://emc.medicines.org.uk (as per SmPC)

Safety profile
The main toxicities associated with carboplatin are:

Haematological toxicity: Myelosuppression is the dose-limiting toxicity of carboplatin. At maximum tolerated dosages of carboplatin administered as a single agent, thrombocytopenia, with nadir platelet counts of less than 50 x 10^9/L, occurs in about a quarter of the patients. The nadir usually occurs between days 14 and 21, with recovery within 35 days from the start of therapy. Leucopenia has also occurred in approximately 14% of patients but its recovery from the day of nadir (day 14-28) may be slower and usually occurs within 42 days from the start of therapy. Neutrogena with granulocyte counts below 1 x 10^9/L occurs in approximately one fifth of patients. Anaemia with haemoglobin values below 11g/dL has been observed in more than two-thirds of patients with normal base-line values.
Myelosuppression may be more severe and prolonged in patients with impaired renal function, extensive prior treatment, poor performance status and age above 65. Myelosuppression is also worsened by therapy combining carboplatin with other compounds that are myelosuppressive.
Myelosuppression is usually reversible and not cumulative when carboplatin is used as a single agent and at the recommended dosages and frequencies of administration.
Infectious complications have occasionally been reported. Haemorrhagic complications, usually minor, have also been reported.

Nephrotoxicity: Renal toxicity is usually not dose-limiting in patients receiving carboplatin, nor does it require preventive measures such as high volume fluid hydration or forced diuresis. Nevertheless, increasing blood urea or serum creatinine levels can occur. Renal function impairment, as defined by a decrease in the creatinine clearance below 60 ml/min, may also be observed. The incidence and severity of nephrotoxicity may increase in patients who have impaired kidney function before carboplatin treatment. It is not clear whether an appropriate hydration programme might overcome such an effect, but dosage reduction or discontinuation of therapy is required in the presence of severe alteration of renal function tests.

Decreases in serum electrolytes (sodium, magnesium, potassium and calcium) have been reported after treatment with carboplatin but have not been reported to be severe enough to cause the appearance of clinical signs or symptoms.
Cases of hyponatraemia have been reported. Haemolytic uraemic syndrome has been reported rarely.

Gastrointestinal toxicity: Nausea without vomiting occurs in about 15% of patients receiving carboplatin; vomiting has been reported in over half of the patients and about one-fifth of these suffer severe emesis. Nausea and vomiting usually disappear within 24 hours after treatment and are usually responsive to (and may be prevented by) anti-emetic medication. A fifth of patients experience no nausea or vomiting.

Cases of anorexia have been reported.

Allergic reactions: Infrequent allergic reactions to carboplatin have been reported, e.g., erythematous rash, fever with no apparent cause or pruritus. Rarely, anaphylaxis, angio-oedema and anaphylactoid reactions, including bronchospasm, urticaria and facial oedema have occurred.

Ototoxicity: Subclinical decrease in hearing acuity, consisting of high-frequency (4000-8000 Hz) hearing loss determined by audiogram, has been reported in 15% of the patients treated with carboplatin. However, only 1% of patients present with clinical symptoms, manifested in the majority of cases by tinnitus. In patients who have been previously treated with cisplatin and have developed hearing loss related to such treatment, the hearing impairment may persist or worsen.
At higher than recommended doses in combination with other ototoxic agents, clinically significant hearing loss has been reported to occur in paediatric patients when carboplatin solution was administered.

Neurotoxicity: The incidence of peripheral neuropathies after treatment with carboplatin is 4%. In the majority of the patients neurotoxicity is limited to paraesthesia and decreased deep tendon reflexes. The frequency and intensity of this side effect increases in elderly patients and those previously treated with cisplatin.

Paraesthesia present before commencing carboplatin therapy, particularly if related to prior cisplatin treatment, may persist or worsen during treatment with carboplatin.

Ocular toxicity: Transient visual disturbances, sometimes including transient sight loss, have been reported rarely with platinum therapy. This is usually associated with high dose therapy in renally impaired patients.

Other: Abnormalities of liver function tests (usually mild to moderate) have been reported with carboplatin in about one-third of the patients with normal baseline values. The alkaline phosphatase level is increased more frequently than SGOT, SGPT or total bilirubin. The majority of these abnormalities regress spontaneously during the course of treatment.

Infrequent events consisting of taste alteration, asthenia, alopecia, fever and chills without evidence of infection have occurred.

APPENDIX 8

EXPECTED ADVERSE EVENTS

	Thoracic Radiotherapy	Cisplatin	Etoposide
**Haematological**			
Anaemia	✓	✓	✓
Leucopenia	✓	✓	✓
Neutropenia			✓
Thrombocytopenia	✓	✓	✓
Febrile neutropenia		✓	✓
**Gastrointestinal**			
Oesophagitis	✓(unless grade 3 or 4)		
Abnormal liver transaminases		✓	✓
Anorexia	✓	✓	✓
Constipation			✓
Diarrhoea		✓	✓
Nausea	✓	✓	✓
Vomiting		✓	✓
**Renal**			
Hyperuricemia		✓	✓
Nephrotoxicity		✓	
**Allergic**			
Rash		✓	✓
Pruritus		✓	✓
Anaphylactic-like (flushing facial oedema, wheezing, tachycardia, hypotension, skin rash, fever)		✓	✓
**Respiratory**			
Bronchospasm		✓	✓
Pneumonitis	✓(unless grade 3 or 4)		
Pulmonary fibrosis	✓		
Dyspnoea	✓	✓	
**Neurotoxicity**			
Peripheral neuropathy		✓	✓
Loss of taste		✓	
Convulsions		✓	
**Skin**			
Desquamation	✓		
Erythema	✓		
**Other**			
Flu like symptoms	✓		
Fatigue	✓	✓	✓
Alopecia		✓	✓
Oral toxicity (soreness & erythema)		✓	✓
Ototoxicity-Tinnitus, hearing loss		✓	
Ocular toxicity		✓	
Vascular toxicity – cerebrovascular accident, myocardial infarction, cerebral arteritis		✓	
Cardiotoxicity – tachycardia,arrhythmia, postural hypotension		✓	
Hypotension			✓
Serum electrolyte disturbances		✓	

APPENDIX 9

COMPLIANCE WITH IR(ME)R 2000 REGULATIONS AND OTHER QA REQUIREMENTS.

All participating centres must comply with the requirements of the IR(ME)R 2000 Regulations and the Medical and Dental Guidance Notes, 2002.

Participating centres must have prior approval by the Local Research Ethics Committee. This will include general approval of the protocols for patient inclusion, patient consent and the justification of radiation exposures (for planning and treatment purposes).

Participating centres must operate a formal system of Quality Assurance in Radiotherapy (QART), which may include registration to an external standard (such as ISO9001:2000).

As part of the QART system, and in compliance with IR(ME)R 2000, participating centres must follow written protocols for radiotherapy treatment planning, prescription and delivery. In these protocols, there should be clear description of compliance with regard to the role of the employer, referrer and operator. The process for justification and authorisation of planning and treatment exposures must be clearly described (appendix 9).

Participating centres must participate in an external programme of dosimetry audit (such as that performed by IPEM). There must be no unresolved dosimetry discrepancies.

References:

IR(ME)R: Ionising Radiation (Medical Exposure) Regulations 2000 (SI 2000 No 1059), HMSO, London.

Medical and Dental Guidance Notes, 2002, Institute of Physics and Engineering in Medicine, York.

International Standard EN ISO 9001:2000. Quality Management Systems – Requirements. BSI, London, 2000.

APPENDIX 10

RADIOTHERAPY QUALITY ASSURANCE

Participating centres should be experienced in the selection of patients with limited stage small cell lung cancer (SCLC) for treatment with curative intent using high dose radiotherapy and chemotherapy, and the administration of such treatment.

The trial will be subject to a quality assurance programme. Centres will have to pass an initial assessment before patients may be randomised in the trial and there will be further assessments afterwards.

The initial assessment will consist of :

- Completion and return of a questionnaire detailing the facilities available to the centre.
- Return of 2 radiotherapy treatment plans for a patient with limited stage SCLC, previously treated in the centre with radical intent, who satisfied the eligibility criteria for the CONVERT trial, and has been replanned according to the CONVERT protocol for each treatment arm; 66Gy in 33 daily fractions and 45Gy in 30 fractions treated twice per day.

If there are several participating clinicians in each centre then a centre may begin to randomise patients after the Principal Investigator has passed the initial QA assessment but all clinicians will be required to submit plans for patients previously treated by themselves before they may independently randomise patients.

The Trial Office will subsequently send each participating centre clinical histories with results of investigations; bronchoscopy, respiratory function tests, CT and PET scans, along with diagnostic and planning CT scans on a compact disc.
In one exercise the participating clinicians will outline the Gross Tumour Volume (GTV). The GTV will be expanded to give the Clinical Target Volume (CTV) and the Planning Target Volume (PTV).
In another exercise the GTV will already be outlined and the dosimetrist will perform expansions to give CTV and PTV (part of a phantom exercise).

Further exercises will include a right sided lung cancer with ipsilateral mediastinal lymphadenopathy and a left sided lung cancer with contralateral mediastinal lymphadenopathy. Clinicians will outline the GTV, expansions will then be performed to give the PTV and dosimetrists will plan for treatment.

During the trial plans will be randomly requested from each clinician in each centre as part of continuing quality assurance.

Participating centres will have to agree to address uncertainties revealed by the QA programme.

The quality assurance program is based at Mount Vernon Hospital
Responsible clinician Dr Ethan Lyn (ethanlyn@mac.com)

RADIOTHERAPY QUALITY ASSURANCE QUESTIONNAIRE

Centre :

Clinicians :

Dosimetrists (Physicists or Radiographers, specify) :

Dedicated Radiographer :

Research Nurse :

Number of patients with limited stage Small Cell Lung Cancer treated with radical intent by centre per year :

Equipment :

Linear Accelerators (LAs) :

Number and type (specify):

Network :

Provision for servicing/breakdown :

Quality Assurance Programme for LAs :

Immobilisation Device (specify) :

Validation study Yes/No

Treatment Planning System (TPS) :

Algorithm (specify type) :

Inhomogeneity correction (specify method) :

Quality Assurance Program for TPS :

APPENDIX 11

SITE PERSONNEL FORM

Full contact details of all trial personnel

Hospital: ______________________ **Principal Investigator:** ______________________

Address: ______________________ **Main contact person** ______________________
(e.g. data queries, general correspondence)

____

____

Complete this form for all trial personnel and return with a brief CV for each
Notify the Christie Hospital CTU of any contact or trial personnel changes
Use additional sheets if necessary

Name ______________________
(title, first name, surname, position)
Department ______________________
Phone ______________________
Fax ______________________
E-mail ______________________
Address ______________________
(if different from above) ______________________

Name ______________________
(title, first name, surname, position)
Department ______________________
Phone ______________________
Fax ______________________
E-mail ______________________
Address ______________________
(if different from above) ______________________

Name ______________________
(title, first name, surname, position)
Department ______________________
Phone ______________________
Fax ______________________
E-mail ______________________
Address ______________________
(if different from above) ______________________

CONVERT: Signature list and delegation of responsibilities

APPENDIX 12

Institution: ____________________

This form must be completed by all personnel managing patients and those responsible for completing CRFs (e.g oncologists, surgeons, pathologists and research nurses/data managers). <u>Only staff who are included on this form will be authorised to sign CRFs.</u>

**Name**	**Job title**	**Sample signature**	**Sample short signature** (initials)	**Responsibilities** (please tick all applicable boxes)
				☐☐ Medical care of patients<br>☐☐ Adverse event reporting<br>☐☐ Ethics/regulatory approval<br>☐☐ Registration/Randomisation<br>☐☐ Informed consent<br>☐☐ CRF completion<br>☐ Trial master file maintenance<br>☐ Drug accountability<br>☐ Pathology specimen processing<br>☐ Pharmacy<br>☐ Laboratory
				☐☐ Medical care of patients<br>☐☐ Adverse event reporting<br>☐☐ Ethics/regulatory approval<br>☐☐ Registration/Randomisation<br>☐☐ Informed consent<br>☐☐ CRF completion<br>☐ Trial master file maintenance<br>☐ Drug accountability<br>☐ Pathology specimen processing<br>☐ Pharmacy<br>☐ Laboratory
				☐☐ Medical care of patients<br>☐☐ Adverse event reporting<br>☐☐ Ethics/regulatory approval<br>☐☐ Registration/Randomisation<br>☐☐ Informed consent<br>☐☐ CRF completion<br>☐ Trial master file maintenance<br>☐ Drug accountability<br>☐ Pathology specimen processing<br>☐ Pharmacy<br>☐ Laboratory
				☐☐ Medical care of patients<br>☐☐ Adverse event reporting<br>☐☐ Ethics/regulatory approval<br>☐☐ Registration/Randomisation<br>☐☐ Informed consent<br>☐☐ CRF completion<br>☐ Trial master file maintenance<br>☐ Drug accountability<br>☐ Pathology specimen processing<br>☐ Pharmacy<br>☐ Laboratory

Please notify us of any changes to trial personnel by updating your original completed copy of this form and sending it to the Christie Hospital CTU.

CONVERT

Concurrent **ON**ce-daily **VE**rsus twice-daily **R**adio**T**herapy: A 2-arm randomised controlled trial of concurrent chemo-radiotherapy comparing twice-daily and once-daily radiotherapy schedules in patients with limited stage small cell lung cancer (SCLC) and good performance status.

**Protocol Version Number:**	**Version 3**
**Protocol Date:**	**10th June 2008**
**Authorised by:**	C Faivre-Finn<br>Dr Corinne Faivre-Finn<br>10/06/08<br>Date

CONVERT is sponsored by Christie Hospital NHS Foundation Trust. The trial is run in collaboration with:

This document describes the CONVERT clinical trial and provides information about procedures for entering patients. The protocol should not be used as an aide-memoire or guide for the treatment of other patients; every care was taken in its drafting, but corrections or amendments may be necessary. These will be circulated to investigators in the trial, but centres entering patients for the first time are advised to contact the trials centre to confirm they have the most up to date version.

Problems relating to this trial should be referred to the relevant member of the trial management group.

This trial will adhere to the principles outlined in the International Conference on Harmonisation Good Clinical Practice (ICH GCP) guidelines. It will be conducted in compliance with the protocol, the Data Protection Act (DPA G0027154) and other regulatory requirements as appropriate.

PROTOCOL DEVELOPMENT GROUP

Chief Investigator:

Dr Corinne Faivre-Finn
Dept Clinical Oncology
Christie Hospital NHS trust
Wilmslow Road
Manchester
M20 4BX
e-mail:corinne.finn@christie.nhs.uk
telephone: +44 (0)161 446 8290
fax: +44 (0)161 446 8142

Sponsor:

Christie Hospital NHS Trust
Wilmslow Road
Manchester
M20 4BX
telephone: +44 (0)161 446 3000

	Host Institution	e-mail
**Clinical oncologists**		
Professor Allan Price	Western General Hospital, Edinburgh	aprice@staffmail.ed.ac.uk
Dr Michael Snee	Cookridge Hospital, Leeds	michael.snee@leedsth.nhs.uk
Dr Rhona McMenemin	Newcastle	Rhona.McMenemin@nuth.northy.nhs.uk
Dr Nazia Mohammed	Beatson Infirmary, Glasgow	Nazia.Mohammed@NorthGlasgow.Scot.N
**Medical oncologists**		
Dr Fiona Blackhall	Christie Hospital, Manchester	Fiona.Blackhall@christie.nhs.uk
Dr Siow Ming Lee	UCL Hospital, London	siow-ming.lee@uclh.nhs.uk
Dr Paul Lorigan	Christie Hospital, Manchester	Paul.Lorigan@christie.nhs.uk
Professor Penella Woll	Weston Park Hospital, Sheffield	p.j.woll@shef.ac.uk
**Medical Physicist**		
Dr Carl Rowbottom	Christie Hospital, Manchester	Carl.Rowbottom@physics.cr.man.ac.uk
**Pathologist**		
Dr Helen Doran	Wythenshawe Hospital, Manchester	helen.doran@smuht.nwest.nhs.uk
**Chest Physician**		
Dr Jan VanMeerbeeck	University of Gent, Belgium	jan.vanmeerbeeck@ugent.be
**Radiologist**		
Dr Stephen Gwyther	East Surrey Hospital	GwytherSJ@aol.com
**Radiographer**		
Michelle Duffy	Christie Hospital, Manchester	michelle.duffy@christie.nhs.uk
**Patient representative**	Janet Power	

**Radiotherapy quality assurance**		
Ethan Lyn	Mount Vernon Hospital	ethanlyn@mac.com
**Research nurse**		
Tina Pritchard	Christie Hospital, Manchester	tina.pritchard@christie.nhs.uk
**Statisticians**		
Ric Swindell	Christie Hospital NHS Trust	ric.swindell@christie.nhs.uk
Linda Ashcroft	Christie Hospital NHS Trust	Linda.ashcroft@christie.nhs.uk
**Pharmacist**		
Susan Arrand	Christie Hospital NHS Trust	susan.arrand@christie.nhs.uk
**Trial Management**		
Helen Flight (maternity leave from Jun08, covered by Sally Falk)	Christie Hospital, Manchester	helen.flight@christie.nhs.uk (maternity leave from Jun08, covered by sally.falk@christie.nhs.uk)
**Trial advisor** Richard Stephens	MRC Clinical Trials Unit, London	rs@ctu.mrc.ac.uk

Trial coordination

Manchester Lung Cancer Group
Christie Hospital Cancer Trials Unit
Wilmslow Road
Manchester
England M20 4BX

general enq: +44 (0)161 918 7101
fax: +44 (0)161 446 8148
e-mail: helen.flight@christie.nhs.uk/
sally.falk@christie.nhs.uk

Randomisation line: +44 (0)161 446 3311

Monday - Friday

09:00 - 17:00 (BST)

Contact details for collaborating groups:

EORTC
Benedicte Marchal benedicte.marchal@eortc.be

Grupo Español de Cáncer de Pulmón
Felipe Cardenal fcardenal@iconcologia.net

Groupe Français de Pneumo-Cancérologie and Intergroupe Francophone de Cancérologie Thoracique
Pierre Fournel Pierre.FOURNEL@icloire.fr

National Cancer Institute of Canada Clinical Trials Group (Trial ID: NCIC CTG BR.28)

Study Chair:	Andrea Bezjak	andrea.bezjak@rmp.uhn.on.ca
Physician Coordinator:	Lesley Seymour	LSeymour@ctg.queensu.ca
Study Coordinator:	Anne Farley	AFarley@ctg.queensu.ca

**CONTENTS**		Page
	Trial summary	7
	Flowchart	8
1.0	**Background**	9
2.0	**Trial design**	12
2.1	Trial objectives	12
2.2	Overall design	12
2.3	Related sub-studies	12
3.0	**Trial entry**	14
3.1	Centre eligibility	14
3.2	Centre entry criteria	14
3.3	Patient entry	15
3.4	Randomisation	16
4.0	**Treatment plan**	18
4.1	Chemotherapy	18
4.2	Thoracic Radiotherapy	20
4.3	Twice daily thoracic radiotherapy	22
4.4	High Dose once daily thoracic radiotherapy	23
4.5	Prophylactic cranial irradiation	24
4.6	Concomitant medication	24
4.7	Criteria for stopping treatment	25
4.8	Therapy after protocol treatment has stopped	25
4.9	Patients not meeting radiotherapy planning requirements	25
5.0	**Pharmacovigilance**	26
5.1	Definition	26
5.2	Causality	27
5.3	Reporting procedures	27
6.0	**Assessments**	29
6.1	Assessments during treatment	29
6.2	Assessments at follow-up	29
6.3	Cost effectiveness	29
6.4	Summary of protocol assessment visits	30
6.5	Study forms	31
6.6	Data handling	32
7.0	**Statistical considerations**	33
7.1	Sample size calculation	33
7.2	General considerations	33
7.3	Qualifications for efficacy analysis	33

7.4	Qualifications for safety analysis	34
7.5	Qualifications for dose intensity analysis	34
7.6	Patient disposition	35
7.7	Patient characteristics	35
7.8	Concomitant therapy	35
7.9	Subsequent therapy	35
7.10	Treatment compliance	35
7.11	Health outcomes	35
8.0	**Informed consent, ethical & regulatory considerations**	37
8.1	Ethical approval	37
8.2	Patient informed consent	37
8.3	Protocol compliance	37
8.4	Indemnity and compensation	37
8.5	Data protection	37
8.6	Duration of the trial	37
8.7	Publication policy	37
9.0	**Independent data monitoring and ethics committee**	38
10.0	**References**	39
11.0	**Glossary**	41
	**Appendices**	
	Appendix 1 - Performance status scale (ECOG)	43
	Appendix 2 – CTCAE v 3.0	44
	Appendix 3 - MRC dyspnoea score	50
	Appendix 4 – Response criteria	51
	Appendix 5 – Cockroft and Gault formula	53
	Appendix 6 - Suggested scheme for administration chemotherapy cycles	54
	Appendix 7 - Chemotherapy agents - safety profiles	55
	Appendix 8 – Expected Adverse Events	59
	Appendix 9 - Compliance with IR(ME)R 2000 Regulations and other QA Requirements	60
	Appendix 10 – Radiotherapy Quality Assurance	61
	Appendix 11 – Site Personnel Form	62
	Appendix 12 – Site Personnel Delegation Log	63
	Appendix 13 – Record of changes made to protocol	64

Summary

Type of design
This is a multicentre randomised phase III trial.
Patients are randomised to one of two treatment arms with 1:1 randomisation:
Patients will be randomly allocated to treatment, using a minimization procedure.

Patients studied
Histologically or cytologically confirmed SCLC with limited disease (Veterans Administration Lung Cancer Study Group) ie patients whose disease can be encompassed within a radical radiation portal. No pleural or pericardial effusions proven to be malignant. RT target volume acceptable by the local radiotherapist. ECOG performance status 0-2.
For more details refer to section 3.

Trial interventions

Control Arm:
Four to six cycles of Cisplatin 25 mg/m^2 iv D1-3 or 75 mg/m^2 D1 Etoposide 100 mg/m^2 iv D1-3 with concurrent BD radiotherapy 45Gy in 30 twice-daily fractions over 3 weeks, 5 days per week
from day 22 of cycle 1

Experimental Arm:
Four to six cycles of_Cisplatin 25 mg/m^2 iv D1-3 or 75 mg/m^2 D1 Etoposide 100 mg/m^2 iv D1-3 with concurrent OD radiotherapy 66Gy in 33 daily fractions over 6.5 weeks, 5 days per week
from day 22 of cycle 1

Accrual
At least 532 patients are to be recruited over 4 years (this is a total for all countries, including patients contributed by collaborating groups).

Outcome measures

Primary end-point

- Overall survival

Secondary end points

- Local progression-free survival
- Metastasis-free survival
- CTCAE v3.0 toxicity
- Chemotherapy dose intensity
- Radiotherapy dose intensity

Duration of assessment
Patients will be assessed prior to each cycle of chemotherapy and at 4 weeks after the final cycle. Follow-up will be at 3 monthly intervals for 12 months and six monthly thereafter until death. Post treatment CT scans thorax and abdomen will be done within 4 weeks of cycle 4 (even if 6 cycles are given) and 6 months after randomisation.

Data collection
Data will be recorded on Case Report Forms (CRFs), a copy should be sent to the Christie Hospital CTU for data entry and a copy kept at the local centre.
For more details refer to section 6.5.

CONVERT trial flowchart

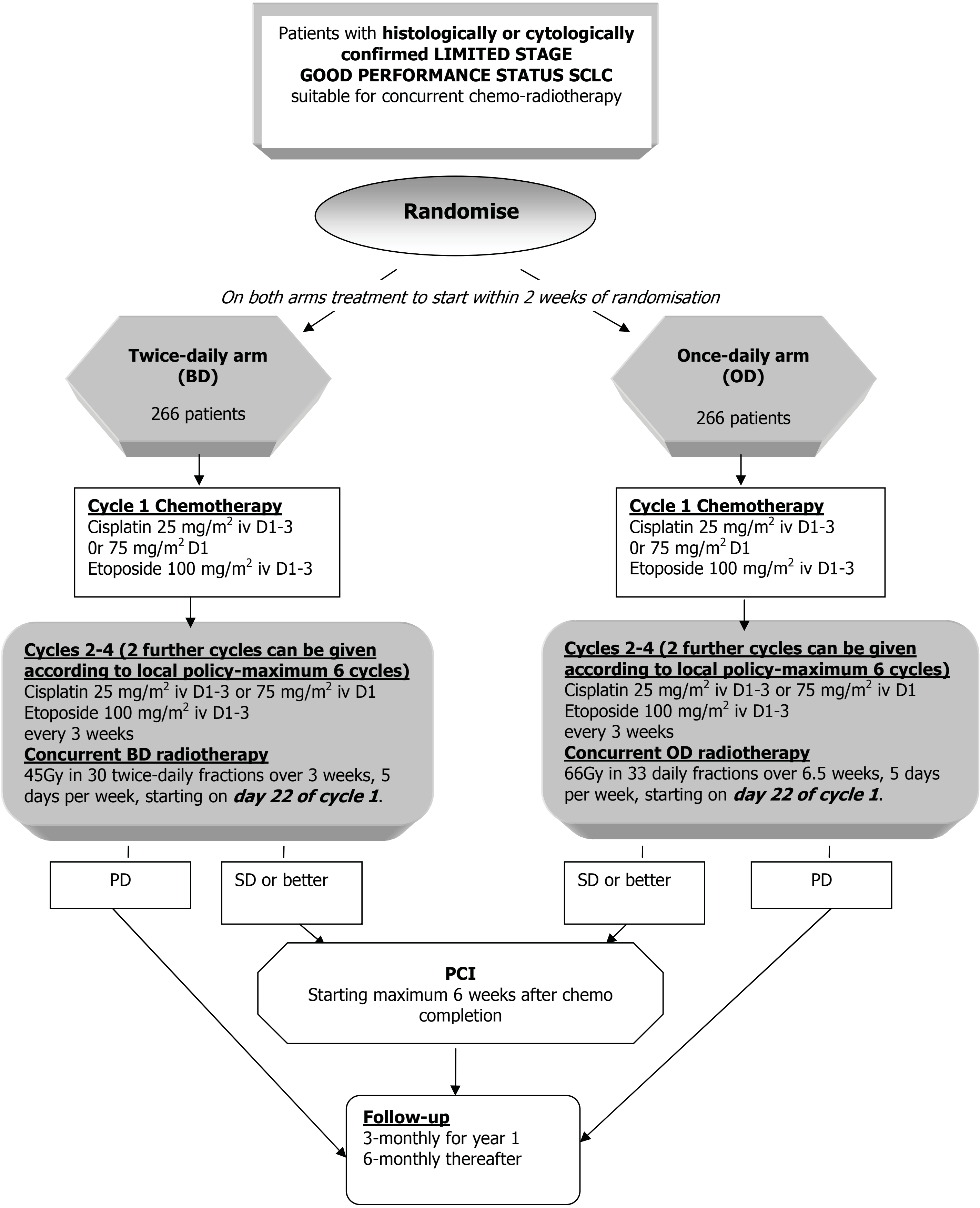

1.0 BACKGROUND

Introduction

Of the 40 000 patients diagnosed with lung cancer each year in Britain, 15 to 20% will have small cell lung cancer. At the time of diagnosis, 30% of patients with small cell carcinoma will have limited stage disease, - that can be encompassed within a tolerable radiation therapy field. However, even in this early stage of disease the outcome is poor, with a median survival of 16 to 24 months with current forms of treatment (1-3).
Combining chemotherapy and thoracic radiotherapy is now the standard treatment for limited stage small cell lung cancer. Indeed two meta-analyses have shown that radiotherapy associated with chemotherapy improves the median survival, the 3 year survival rate and the local control (4,5). In patients who present with small volume limited stage SCLC (ie confined to lung and mediastinum only) there is a real possibility of long-term survival if optimum treatment is given. In this group of patients, a US trial (3) comparing once vs twice daily concurrent chemo-radiotherapy reported >40% survival at 2 years in both arms. This compares with around 15% in the recent UK MRC LU21 trial (6) which used sequential chemo-radiotherapy. There is a real need to improve these UK figures.

Although thoracic radiotherapy is now integrated in the routine treatment of LS SCLC there are numerous questions surrounding the optimum way to combine radiotherapy and chemotherapy. Several important questions remain unanswered including the optimal total radiation dose, radiation fractionation, timing of radiation, sequencing of radiation and volume of irradiation.

Numerous prospective studies have tested various doses, duration, fractionation and timing of thoracic radiotherapy

- Three randomised trials (1,7,8) are in favour of early thoracic irradiation, whereas 3 other randomised studies (9,10,28) did not demonstrate a difference between early and late chest radiotherapy.
- A recent meta-analysis has evaluated early versus late timing of thoracic radiotherapy in combined modality therapy for limited-stage small cell lung cancer. Early radiation therapy was defined as prior to 9 weeks after initiation of chemotherapy (i.e. at the latest with the third cycle of chemotherapy). There was a benefit in overall survival for early radiation therapy versus late radiation therapy (11). In contrast a recent Cochrane review (29) which also included 7 trials (replacing Gregor et al with James et al) concluded that it is unclear whether the timing of chest radiotherapy (beginning within 30 days of the start of chemotherapy or later) affects long term survival. The evidence for starting concurrent radiotherapy with cycle 1 chemotherapy is controversial. In the UK, starting radiotherapy with the first cycle of chemotherapy poses considerable logistical issues. Therefore it appears reasonable to start the radiotherapy treatment during the second or the third cycle of chemotherapy.
- Concurrent chemo-radiotherapy seems to be superior to sequential treatment (7). Indeed if radiotherapy is given early and concurrently with chemotherapy, not only very sensitive cells, but also some less sensitive or highly proliferative cells will be killed, because of the synchronous action of the chemotherapy drugs, together with the effect of radiation. When radiation is given later, often not concurrently with chemotherapy, only the very sensitive cancer cells will be killed by radiotherapy, but not the very rapidly proliferating ones. The Japan Clinical Oncology Group Study (7) is the only trial which has randomised patients between sequential and concurrent chemo-radiotherapy (Sequential: 4 cycles 3-weekly cisplatin 80mg/m2 d1, etoposide 100mg/m2 d1-3, followed by 45Gy/30f BD, Concurrent: 4 cycles 4-weekly cisplatin 80mg/m2 d1, etoposide 100mg/m2 d1-3, with 45Gy/30f BD starting on day 2 of cycle 1). Although the trial showed a survival benefit at 5 years for the concurrent arm (23.7% vs 18.3%) this was not statistically significant (p=0.097) possibly due to the fact that only 228 patients were included in the trial. However the survival benefit was achieved despite the fact that the chemotherapy dose-intensity was inferior in the concurrent arm (given 4-weekly) compared to the sequential arm (given 3-weekly).

- Hyperfractionated radiotherapy has been shown to be more efficacious than standard fractionation in in-vitro studies (13). Fractionating the dose into 2 treatments each day has radiobiological advantages. Small cell lung cancer cells lines are sensitive to small doses of radiation. In addition the use of small doses per fraction will diminish the late damages to the normal tissues. The value of twice daily (BD) thoracic radiotherapy given early and concurrently with PE chemotherapy was examined in a landmark trial (3). The regimens were: once-daily (OD): 4 cycles 3-weekly cisplatin 60mg/m2 d1, etoposide 120mg/m2 d1-3, 45Gy/25f starting d1 of first cycle, BD 4 cycles 3-weekly cisplatin 60mg/m2 d1, etoposide 120mg/m2 d1-3, 45Gy/15f starting d1 of first cycle). This study showed that twice-daily treatment beginning with the first cycle of chemotherapy significantly improves survival as compared with concurrent once daily radiotherapy (p=0.04). After a median follow-up of 8 years, the survival rates for patients receiving once-daily chemotherapy were 41% at 2 years and 21% at 5 years. For patients receiving twice-daily radiotherapy corresponding figures were 47% and 26%. However grade 3 oesophagitis was significantly more frequent with twice-daily radiotherapy, occurring in 27% of patients, as compared with 11% in the once-daily group (p<0.001). No long term strictures were reported.
  One of the main criticisms of the study is that in the once-daily thoracic irradiation arm the rate of local failure was very high (52%), which demonstrated that a total dose of 45 Gy given once-daily is too low. The only other trial comparing OD and BD radiotherapy in this group of patients reported no survival differences although the long-term results for both arms were still encouraging with 5 year survival rates of >20% in each arm (14,15). The regimens compared were OD: 6 cycles 4-weekly cisplatin 30mg/m2 d1-3 and etoposide 100mg/m2 d1-3, with 50.4Gy/28f given concomitantly with chemotherapy cycles 4 and 5, BD: same chemotherapy but with 24Gy/16f BD starting d1 cycle 4, then a 28 day break followed by another 24Gy/16f BD (starting d1 cycle 5). Because of the split-course policy the twice-daily radiotherapy took as long to deliver as conventional once-daily radiotherapy (overall radiotherapy treatment time was 38 days in both arms), resulting in no overall acceleration in the hyper-fractionated arm. An additional potential weakness of the split-course approach is that tumour re-population is likely to occur during the break in treatment allowing chemotherapy resistant clones to develop. The Turrisi trial showed improved survival for the BD arm (47% vs 41% at 2 years, 26% vs 21% at 5 years). However as overall radiotherapy treatment time differed substantially between the two groups (3 weeks for the BD regimen and 5 weeks for the OD regimen), it was unclear whether the survival differences were due to altered fractionation or shorter treatment time or both.

- As far as the total dose of radiation concurrent with chemotherapy is concerned, Choi et al showed that the maximum tolerated dose for thoracic irradiation is at least 70 Gy in 35 fractions over 7 weeks for daily thoracic irradiation and 45 Gy in 30 fractions over 3 weeks for twice-daily thoracic irradiation (16).

Rationale for a phase III trial

The fractionation and overall time of treatment questions have been specifically addressed by the landmark Turissi study published in 1999 (3).
We are currently in the position that as a result of the Takada and Turrisi trials concurrent BD chemo-radiotherapy is widely accepted as standard treatment in the US and Europe for this group of patients. Although the Turrisi trial was published in 1999 it has not been repeated and it is very important to discern whether the survival benefit seen with BD fractionation is a true effect, especially as the RT schedule in control arm of the trial would now be considered sub-optimal, resulting in a very high (52%) local failure rate. More recent studies by Choi et al using once daily radiotherapy and Komaki et al (16, 17) using a concomitant boost technique have suggested that doses of 70Gy over 7 weeks and 61.8Gy over 5 weeks are possible, the former being delivered with 5 cycles of full dose chemotherapy.
Moreover since the Turrisi trial was designed in the late 1980s, progress has been made in radiotherapy techniques. The radiotherapy used in any contemporary trial would be CT-planned conformal treatment with individual blocking, careful calculation of target, lung and oesophageal doses based on modern data on tolerance and response, with verification using on-line portal imaging, correction of set-up errors, and allowance made for the effects of respiratory movement on the position of the target volume. None of these were routine from 1989-1992 when the Turrisi trial was carried out. In addition there has been concern that the toxicity of twice-daily thoracic irradiation concomitant with chemotherapy is too high. In the Turrisi trial grade 3 oesophagitis (defined as inability to swallow more than liquids or to require hospitalisation) occurred

in 27% of patients in the twice-daily group compared with the 11% in the once daily group. However, oesophagitis has not caused permanent or life-threatening damage in the trials using non-anthracycline based regimens.

Before twice-daily thoracic irradiation is accepted as a standard for the treatment of limited stage small cell lung cancer, it is important to compare this schedule with a higher dose of radiation delivered once daily. Indeed the toxicity of twice-daily thoracic irradiation concomitant with chemotherapy is high and the cost and inconvenience of such treatment is also to be considered. Using the modern facilities of conformal 3D radiotherapy and limiting as much as possible the amount of normal tissues irradiated, doses higher than 50 Gy should be evaluated.

In view of the lack of data in the literature addressing the question of the dose and fractionation for limited stage small cell lung cancer, we propose to perform a new randomised phase III trial to try to establish a standard chemoradiotherapy regimen for LS SCLC with good performance status. There are currently no international trials for this group of patients, and thus an opportunity exists to set up a global trial to answer this important question. The results of the trial will be crucial in determining the best international standard treatment for routine clinical use in the treatment of patients with limited-stage SCLC and good performance status. In addition the translational studies carried out in parallel to CONVERT will indicate the hypotheses which need testing in the next generation of trials in this disease.

Choice of chemotherapy regimen

The combination of cisplatin and etoposide (PE) is the standard chemotherapy treatment in this group of patients. The use of PE is supported by 2 reviews: firstly a large systematic review of 7173 randomised patients which found that the inclusion of either cisplatin (22 trials) or etoposide (37 trials) in a chemotherapy regimen was associated with a significant survival improvement, as was the inclusion of both drugs compared to chemotherapy regimens with neither (18), and secondly a meta-analysis of 19 trials of 4054 patients which confirmed an increased survival with the inclusion of platinum of 2.6% and 4.4% at six months and one year respectively (19), and also, in a subset analysis of nine trials, showed a similar survival benefit for the inclusion of etoposide. A subsequent randomised trial of PE vs epirubicin, cyclophosphamide and vincristine in 440 patients supported this conclusion by showing that PE resulted in a better survival (20). Attempts to improve on this combination have met with limited success (21-23). The combination of Etoposide and Cisplatin has become in recent years the treatment of choice as it can be used concurrently at full doses with radiation therapy with acceptable toxicity (1,3,7). It is also simple to use in ambulatory patients.

The Canadian small cell lung cancer guidelines published in 2004 (24) are based on a systematic review and give a range of recommended regimens for concurrent chemo-radiotherapy, including cisplatin 25mg/m2 days 1-3 or 75 mg/m2 d1 with etoposide 100mg/m2 d1-3. There is no good evidence for higher doses of etoposide which result in an increased risk of haematological toxicity, the use of GCSF, and dose delays. The guidelines state that there is insufficient evidence to comment on whether 4 cycles are the same as 6, whether oral is equivalent to iv etoposide, or whether carboplatin can be substituted for cisplatin. Therefore there is a strong rationale for using 4-6 cycles of cisplatin 25mg/m2 d1-3 (or cisplatin 75mg/m2) and etoposide 100mg/m2 d1-3.

Choice of radiotherapy regimen

- BD regimen

Commencing radiotherapy on day 1 of chemotherapy is not feasible as the process of CT planning, volume contouring, treatment optimisation, and set-up checking takes 1-2 weeks. Hence the most feasible BD regimen would be 4-6 cycles of cisplatin 25 mg/m2 d1-3 or 75 mg/m2 d1 and etoposide 100 mg/m2 d1-3, q3w with 45Gy in 30 fractions over 3 weeks commencing on day 1 of cycle 2 of chemotherapy. This would differ from the BD regimen in the Turrisi trial in that the cisplatin dose would be increased (25mg/m2 d1-3 or 75 mg/m2 d1 rather than 60mg/m2 d1), the etoposide dose would be decreased (100mg/m2 d1-3 rather than 120 mg/m2 d1-3), and radiotherapy would start with cycle 2 rather than cycle 1.

- High dose OD regimen

A schedule of 66Gy in 33 daily fractions over 6.5 weeks has been piloted successfully with good compliance and toxicity profile (presented as a poster at the BTOG meeting in Dublin 2005) and is the subject of an existing single centre phase II study in Manchester.

2.0 TRIAL DESIGN

2.1 TRIAL OBJECTIVES

Primary end-point

- Overall survival

Secondary end points

- Local progression-free survival
- Metastasis-free survival
- CTCAE v3.0 toxicity
- Chemotherapy dose intensity
- Radiotherapy dose intensity

2.2 OVERALL DESIGN

This is a multicentre randomised phase III trial.
Patients are randomised to one of two treatment arms with 1:1 randomisation:

A 2-arm randomised trial comparing:

- 45Gy in 30 fractions BD radiotherapy schedule (given concurrently with cisplatin/etoposide)
- 66Gy in 33 fractions OD radiotherapy schedule (given concurrently with cisplatin/etoposide)

2.3 RELATED SUB-STUDIES

- **Correlative molecular studies for the CONVERT trial**

Progress in treatment of SCLC has been hampered by limited understanding of the molecular biology of this disease. It is usually diagnosed on a small biopsy specimen or fine needle aspirate insufficient for detailed molecular studies. Consequently, existing SCLC tumour banks include relatively small series (<100 patients) of samples collected over many years from patients who are heterogeneous with respect to stage and treatment received. The CONVERT trial provides a potentially unique opportunity to prospectively collect a large number of biospecimens from patients of uniform (limited) stage, who are exposed to the same chemotherapy, treated with one of two radiotherapy schedules, and for whom there will be robust clinical outcome data. Although it will still be problematic to obtain large tumour biopsy specimens for many patients, advances in genomic and proteomic technology will enable studies to be performed on blood/serum samples in addition to small biopsy specimens.

- **Biospecimens to be collected**

All patients will be asked to consent for collection of tumour samples (paraffin embedded*)* and blood samples as part of the trial. Blood samples (for genomic and proteomic analysis) will be collected at three timepoints: at baseline prior to any treatment, on day 22 of treatment and on completion of treatment.

- **Molecular studies to be performed**

The objectives of the molecular studies will be to gain improved insight into the molecular biology of SCLC, discover and/or validate candidate biomarkers for response, resistance or toxicity of treatment, and identify novel treatment targets for therapeutic control. The latter is a major objective since the treatments to be evaluated in this trial are anticipated to result in 55% 2 year survival at best. The specific correlative studies have yet to be defined but we anticipate that studies will employ a range of techniques including immunohistochemistry, proteomic profiling (blood/serum) and genetic studies on host and tumour DNA. It is unlikely that there will be sufficient tissue available (and no fresh frozen tissue) for gene expression profiling by cDNA or oligonucleotide microarray. Given that this biobank will be a unique resource the molecular analyses will be conducted where possible taking into account preliminary data and hypotheses generated from studies in SCLC cell lines and xenografts, and from current studies in large patient populations that aim to evaluate host genomic biomarkers of radiation toxicity or cancer predisposition. Samples will be collected globally then stored and analysed in the UK.

3.0 TRIAL ENTRY

3.1 CENTRE ELIGIBILITY

All participating centres must:

a) operate a formal system of Quality Assurance in Radiotherapy (QART), appendix 9.
b) confirm that the initial stage of the radiotherapy quality assurance exercise has been successfully conducted (appendix 10) and that the CONVERT QA questionnaire was completed
c) have conformal radiotherapy planning facilities (defined as multislice CT scanning planning on maximum 5 mm slices, customized blocking, DVH calculations for target and organs at risk, portal image verification) and a radiotherapy verification protocol
d) have discussed suitable patients in a Multi-Disciplinary Meeting, including a clinician with a special interest in lung cancer
e) have a formal protocol for neutropenic sepsis

A centre agreement and checklist must be completed by each centre, to ensure that they are able to comply with the protocol and data collection requirements, prior to site-specific assessment.

3.2 CENTRE ENTRY CRITERIA

3.2.1 Centre Accrediation

The following documentation must be received at the Christie Hospital Clinical Trials Unit in order for an institution to become an approved CONVERT institution:

• Confirmation that the initial stage of the radiotherapy Quality Assurance (QA) exercise has been successfully completed (See Section 3.1)

• Confirmation of favourable local research ethics committee site-specific assessment (SSA)

• A copy of the most recent version of the patient information sheet (appendix 11) and consent form (appendix 12) on local headed paper

• Signed memorandum of understanding (signed by the institution PI)

• Completed Delegation Log (signature list and delegation of responsibilities, appendix 14)

• Full contact details for all site personnel (appendix 13)

Once all of this documentation has been received, confirmation of institution approval will be sent to the Prinicipal Investigator at each institution by the trial team at the Christie Hospital Clinical Trials Unit.

3.3 PATIENT ENTRY

3.3.1 Pre- randomisation evaluation

a) History and examination.
b) Assessment of performance status and dyspnoea score (appendix 1 and 3)
c) Contrasted CT scan of the thorax and upper abdomen (within 4 weeks prior to randomisation)
d) Contrast-enhanced CT (or MRI) scan brain (within 4 weeks prior to randomisation)
e) Pleural aspiration and cytology if possible if patient presents with a pleural effusion
f) Pulmonary function tests [FVC, FEV1, KCO and DLCO/VA)]
g) FBC and serum biochemistry including LDH.
h) Assessment of renal function (see appendix 5)
i) ECG
j) Bone scan need only be performed if there is a specific clinical indication
k) Pregnancy test (if applicable)
l) PET scan or PET-CT, if available. Centres who decide to use PET scans for staging will have to continue doing so for all patients entered in the trial.

3.3.2 Inclusion/exclusion criteria

Queries about eligibility criteria should be addressed prior to randomisation. Patients are eligible for the trial if all of the following criteria are met:

a) Either sex, age ≥18 years
b) Performance status ECOG grade 0-1 (appendix 1). Patients with PS 2 whose general condition is explained by obstructive/bulky disease likely to improve after the first cycle of chemotherapy can be included at the discretion of the local investigator. Patients with PS 2 as a result of comorbid conditions will be excluded.
c) Histologically or cytologically confirmed SCLC
d) No patients with mixed small-cell and non-small-cell histologic features
e) No history of previous malignancy in the last 5 years (except non melanomatous skin or in-situ cervix carcinoma). Patients with previous malignancies (except breast cancer) and in remission for at least 5 years can be included.
f) Limited stage disease (Veterans Administration Lung Cancer Study Group) ie patients whose disease can be encompassed within a radical radiation portal.
g) No pleural or pericardial effusions proven to be malignant
h) RT target volume acceptable by the local radiotherapist
i) Pulmonary function
    a. FEV1 >1 litre or 40% predicted value
    b. KCO (DLCO/VA) >40%predicted
j) Maximum of one of the following adverse biochemical factors:
    a. Serum alkaline phosphatase more than >1.5 times the upper limit of normal (ULN)
    b. Serum sodium < Lower limit of Normal
    c. Serum LDH > ULN

k) Normal serum creatinine and calculated creatinine clearance ≥ 50 ml/min. If calculated creatinine clearance is <50 ml/mn according to the Cockroft and Gault formula (appendix 5), an EDTA clearance should be performed
l) Adequate haematological function
    a. Neutrophils >1.5 x 10^9/l
    b. Platelets >100 x 10^9/l
m) Adequate liver function: ALT & AST <= 2.5 x ULN
n) No other previous or concomitant illness or treatment which in the opinion of the clinician will interfere with the trial treatments or comparisons
o) No prior surgical resection of the primary tumour, no prior radiotherapy for lung cancer
p) Considered fit to receive any of the trial regimens
q) Female patients must satisfy the investigator that they are not pregnant, or are not of child-bearing potential, or are using adequate contraception. Men must also use adequate contraception, as etoposide is clastogenic.
r) Patients must not be breastfeeding
s) Patient has read the patient information sheet and has signed the consent form.
t) Patients available for follow-up

3.4 RANDOMISATION

Randomisation will be administered centrally by the trials office at the Christie Hospital. This is a multi-centre randomized phase III trial. Patients will be randomized on a 1:1 basis to one of two treatment arms. The allocation method will be minimization with a random element. Randomisation will be implemented in a bespoke computer application at the randomisation centre. The factors controlled for in the allocation will be:

Institution
Planned number of cycles (4 or 6)
Performance status (0/1 or 2) {PS2 equivalent to KP 50, 60}

The arm that would result in less imbalance will be allocated with probability 0.75. There will be a 'burn-in' period of 20 cases in which allocation will be completely random.

Randomisation can only be performed **after** confirmation that the patient is eligible (including recording of LDH, sodium and alkaline phosphatase results) and that the patient has signed consent. The system used does not permit any editing of fields by users after arm allocation has been performed.

Randomisation lines:
Tel: +44 (0)161 446 3311 (open Mon-Fri, 9am - 5pm BST)
Fax: +44 (0)161 446 8148

Randomisation by telephone: The researcher calls the randomisation line and supplies a verbal password for the study. A checklist is checked over the telephone before randomisation. Randomisation will be performed using the computer system, callers will be advised of the patient's trial number and treatment allocation. Please record these on the eligibility checklist and return a copy to the trials office (fax 0161 446 8148). An e-mail confirmation will be sent to pre-arranged e-mail addresses for each centre.

Randomisation by fax: The randomization form must be completed, signed by an authorised member of staff and faxed to the fax randomisation line. On receipt of the fax staff at the trials office will check the eligibility checklist and perform the randomisation. An e-mail confirmation will be sent to pre-arranged e-mail addresses for that centre.

This trial is a comparison of treatment polices, once a patient has been randomised that patient remains in the trial and full documentation is always required. Inevitably some patients will stop treatment early, either because the patient does not wish to continue or because the responsible clinician feels it is not in the patient's best interest to continue. For patients who stop treatment early the responsible clinician should continue to complete as much clinical information as possible. As a minimum, follow-up data on progression and survival status should be returned regularly (unless explicit consent for this is withdrawn).

4.0 TREATMENT PLAN (For approximate timing of treatments see appendix 6)

Prior to commencing treatment patients who are current smokers should be advised of the benefits of stopping smoking, and should be offered any available support to quit. Stopping smoking is associated with a reduction in the risk of radiation induced side effects and in some patients also results in improved survival

4.1 CHEMOTHERAPY

Patients should start chemotherapy within two weeks of randomisation and within two weeks of a clinical assessment of fitness (section 4.1.1). Chemotherapy is cisplatin and etoposide and is given for 4 to 6 cycles. One cycle is given prior to radiotherapy. The second cycle in both arms is given concurrently with radiotherapy if possible. Radiotherapy will start on day 22 of cycle 1. In the BD radiotherapy arm, the third to sixth cycles (if applicable) are given after completion of the thoracic radiotherapy. In OD radiotherapy arm, the third & fourth cycles of PE are also given concurrently and the fifth & sixth cycles (if applicable) are given after completion of the thoracic radiotherapy.

Centres will be given the choice to stop chemotherapy after 4 cycles or to continue to up to 6 cycles. Centres who decide to give 6 cycles will have to continue doing so for all patients entered in the trial (unless it is decided that it is not in the patients best interest to receive cycle 5 and 6 or due to patient's choice)

4.1.1 Clinical assessments (see section 6.5)

Assessments should be repeated as necessary, and are required prior to each chemotherapy cycle:

- Clinical examination
- Assessment of performance status (see appendix 1), 'dyspnoea score' (see appendix 3) and toxicity
- FBC, U&Es and LFTs:
    - Bone marrow reserve should be adequate for chemotherapy (i.e. absolute neutrophil count > 1.5 x 10^9/l and platelet count > 100 x 10^9/l)
    - Biochemistry
- Assessment of renal function:
    - Adequate renal function - defined by GFR $\geq$ 50 ml/min (measured by EDTA). The Cockcroft and Gault formula (see appendix 5) may be used to estimate GFR, but if < 50 ml/min then EDTA should be performed.
- Disease status – a CXR will be done prior to cycle 1, 3 and 5 (if 6 cycles of chemotherapy are given). If a CXR shows suspicion of progressive disease a CT scan thorax and abdomen should be done to confirm progression before stopping chemotherapy. A formal assessment with repeat CT scan should be made within 4 weeks after completion of the concurrent chemoradiotherapy.

4.1.2 Chemotherapy Administration

The chemotherapy doses should be based on the patient's calculated pre-treatment body surface area using actual body weight. A detailed plan for administering a cycle of chemotherapy including suggested hydration and anti emetics is given in appendix 6.

In summary, centres can opt for one of the two chemotherapy regimen. Cycles are given at 3 weeks intervals:

- Etoposide 100 mg/m^2 iv D1-3
  Cisplatin 75 mg/m^2 iv D1

Or

- Etoposide 100 mg/m^2 iv D1-3
  Cisplatin 25 mg/m^2 iv D1-3

Patients should be encouraged to take a high oral fluid intake on the day prior to cisplatin chemotherapy.

4.1.3 Dose modifications

The policy should be to delay and give at full dose, rather than reduced dose. The dose modification schedule should be followed, but clinical judgement should be used in individual cases. Common side effects are listed in appendices 7 & 8.

Haematological toxicity

Dose modifications are based on each pre-treatment blood count.

ANC x 10^9/l		Platelets x 10^9/l	Cisplatin /Etoposide
> 1.5	And	>100	Full dose
≤ 1.5	Or	≤ 100	Delay until recovery
Febrile neutropenia or treatment delay for grade 4 neutropenia > 7 days	Or	Grade 4 platelets requiring medical intervention <u>or</u> ≥ grade 2 bleeding with thrombocytopenia	First event: full dose and GCSF support (see paragraph 4.6) is recommended **or** 20% dose reduction<br>Second event: 30% dose reduction<br>Third event: off trial

- If chemotherapy cannot be administered after a three-week delay because of haematological toxicity, chemotherapy should be discontinued.
- Use of GCSF see paragraph 4.6
- If Hb<10 a blood transfusion will be required

Non-haematological toxicity

Hepatic toxicity

AST/ALT		Bilirubin	Cisplatin	Etoposide
2-5 x ULN	and	< 1.5 x ULN	Full dose	Full dose
>5 x ULN	or	>1.5 x ULN	Delay one week then reassess using the same criteria; if delayed for two weeks discontinue	

Renal toxicity

- Request GFR *(EDTA clearance)* before each course of chemotherapy ***If*** creatinine clearance < 50 ml/min
- If GFR re-estimated please dose according to the table:

GFR	Cisplatin	Etoposide
≥ 50 ml/min	Full dose	Full dose
40-49 ml/min	50% dose reduction or substitute with carboplatin AUC 5	20% dose reduction
< 40 ml/min	Off trial	Off trial

Other toxicities (see appendix 2)

Peripheral neuropathy ≥ grade 2	Substitute carboplatin AUC 5 or 50% cisplatin dose reduction after recovery to ≤ grade 1; 100% dose of etoposide
Any grade 3-4 toxicities except mucocitis and alopecia	25% dose reduction for cisplatin and etoposide after recovery to ≤ grade 1

4.1.4 Carboplatin dosing

If carboplatin is to be used, the dose uses target area under the curve of 5 and should be calculated according to the Calvert formula:

Dose (in mg) = Target area under curve x (GFR+ 25)

Carboplatin and etoposide will be given 3 weekly

4.2 THORACIC RADIOTHERAPY

Thoracic radiotherapy should start 21 days after day 1 of the first cycle of chemotherapy (day 22). A delay with the administration of the second cycle of chemotherapy **should not delay** the start of radiotherapy

4.2.1 Clinical assessment (see section 6.5)

Weekly assessments are required during treatment.

- Clinical examination
- Haematological assessments.

4.2.2 General radiotherapy details

Patients should be treated on a linear accelerator operating at 4-10 MV.

The total dose of radiotherapy will be

- BD arm: 45 Gy in 30 twice daily fractions of 1.5 Gy
- OD arm: 66 Gy in 33 daily fractions of 2 Gy

The total dose is prescribed at the ICRU reference point and given according to the recommendations of the EORTC radiotherapy group (25) and ICRU 50 (26). Treatment will be planned with inhomogeneity corrections.

IMRT will be permitted for the centres routinely using it for the treatment of lung cancer.

i. **Radiation Quality Assurance:**

The radiotherapy quality assurance program will be run by the Mount Vernon Hospital QA team (appendix 10). Planning information, copies of portal images (digital print-outs are acceptable) and dose distribution, including Dose Volume Histogram (DVH), with a copy of the treatment prescription should be available for central review. Details of radiotherapy practice will be established by completion of a questionnaire.

Treatment machine beam output data on the linear accelerators used to treat patients in the trial should have been audited by a recognised ASTRO/ESTRO or UK quality assurance programme within 12 months of the first patient in the centre entering the trial, and be rechecked at least once every 18 months

We recommend that daily verifications should be done with open orthogonal images incorporating stable anatomical structures such as the spine for the first 3 days of treatment followed by weekly verifications. If available cone beam imaging can be used as an alternative to open orthogonal images. The orthogonal images will be checked by a senior radiographer and if possible reviewed by a qualified radiation oncologist, and compared with either the digitally reconstructed images or simulator images. The correction decision will be left to local policy. Differences of less than 0.5 cm from the initial images will be allowed. We suggest that if the difference is more than 0.5 cm the orthogonal images will be repeated prior to patients treatment. The Radiotherapy Quality Assurance Group will approve and monitor each centre procedures.

ii. Patient treatment position:

Supine, with arms above head. Immobilisation using chest board and fixed arm position. The patient should be breathing normally.

iii. Patient data acquisition:

A planning CT scan should be performed in the treatment position, whilst the patient undertakes a normal respiration, using 5 mm slices or less through the entire target volume. The whole thorax (cricoid to L2) should be covered using at least 1 cm slices to allow dose-volume histograms to be calculated for the lung, heart and the oesophagus.
Radiotherapy should be started within 3 weeks of planning.

iv. Planning target volume (PTV)

The CT data will be transferred to the planning system.

- The GTV (gross tumour volume) will be contoured by a qualified radiation oncologist specialised in thoracic malignancies. The contouring should be carried out using the mediastinal and the lung windows. The GTV is defined as identifiable tumour and involved lymph nodes (nodal involvement on CT scan is defined as nodes ≥ 1 cm in short axis). If PET scan is available for staging, the GTV should include PET positive lymph nodes.
- The CTV (clinical target volume) comprises the GTV with a 0.5 cm margin of radiologically normal tissue in all directions. It will take into account microscopic spread. Manual adjustment of CTV is permitted to reduce dose to the spinal cord for example, when disease is adjacent to a structure such as a vertebra but is not thought to invade the structure
- The PTV comprises the CTV with a 1 cm margin superiorly and inferiorly, and 0.8 cm margin laterally, at the 95% isodose. The CTV to PTV expansion should not be reduced as it is allowing for set up errors and organ motion.

Prophylactic nodal irradiation should not be employed.

Field reductions will not be allowed.

v. Treatment planning

Use of 3D conformal technique is required and beam's eye views may be useful in the design of individual shielding. Dose volume histograms (DVH) for the PTV, normal lung, oesophagus, spinal cord and heart will be calculated in order to obtain full knowledge of the 3D dose distribution.

vi. Dose specification and fractionation:

The dose will be specified at the ICRU reference point and fully corrected for heterogeneity. The dose distribution within the PTV should ideally be within ± 5% of the prescribed dose, and no more than ± 7% of the prescribed dose.

vii. Definition of the organs at risk

The spinal cord, lungs, oesophagus and heart will be contoured for dose-volume histograms.

- Both the right and left lungs should be contoured as one structure. Contouring should be carried out using pulmonary windows. All inflated lung should be contoured.
- The spinal cord will be contoured based on the bony limits of the spinal canal. The spinal cord should be contoured starting at least 10 cm above the superior extent of the PTV and continuing on every CT slice to at least 10 cm below the inferior extent of the PTV.
- The oesophagus will be contoured using mediastinal windowing on CTscan to correspond to the mucosal, submucosa, and all muscular layers out to the fatty adventitia. The oesophagus should be fully contoured (from cricoid cartilage to the gastro-oesophageal junction)
- The heart will be contoured along with the pericardial sac. The superior aspect (or base) for purposes of contouring will begin at the level of the superior aspect of the left atrium and extend inferiorly to the apex of the heart.

viii. Beams

Isocentric treatment technique.
The number of beams will vary according to the position and the volume of the PTV in the thorax and to the maximum dose tolerated by the organs at risk.
The treatment plan will be checked by a qualified radiation oncologist after discussion with the planning team. The dose-volume histogram will help to guide that choice.

ix. Set-up verification

Cone beam or orthogonal images will be obtained on days 1 to 3 (or 2 to 4) and weekly thereafter.

4.3 TWICE DAILY THORACIC RADIOTHERAPY

4.3.1 Schedule

- **45 Gy** in **30 twice daily fractions** over a period of **19 days** (radiotherapy to start on a Monday)**,** 5 consecutive days a week
- The optimal overall treatment time should be 19 days, up to 21 days is a protocol deviation and should be recorded, above 21 days is a protocol violation.
- The interfraction interval will be 6 to 8 hours.
- using conformal radiotherapy and 4 to 10 MV photons emitted from linear accelerators.

- Thoracic radiotherapy will start on cycle 1 day 22, if possible concurrently with the second cycle of chemotherapy (within 24 hours of day 1, cycle 2 of PE).
- Concurrent chemotherapy will be administered during the intervals between the 2 daily radiotherapy fractions.

4.3.2 Normal tissue constraints

To reduce late damages to the normal tissues the following rules will be applied

- Dose: **1.5 Gy** per fraction
- Maximum spinal cord dose will not exceed **42 Gy.** The spinal cord position must be identified throughout the PTV
- The percentage of lung minus PTV receiving more than 20 Gy will not exceed **35%** (V20=35%, based on dose-volume histograms). The mean lung dose will be recorded
- The heart can receive the total dose (TD) to < 30% of its volume. For > 50% of cardiac volume, dose < 50% of TD is recommended

4.3.3 Treatment Delays

Every effort should be made to deliver the prescribed dose of radiotherapy in 19 days.

If unavoidable delays occur, that could increase the overall treatment time beyond 19 days, e.g. due to machine breakdown, compensation should if possible be made by one of the following mechanisms:

- treating on a weekend day, ***or***
- adjusting fraction size to deliver the total prescribed dose within 19 days;
  However, fraction size should remain < 2.25 Gy

If the radiation schedule is interrupted for more than 1 week due to intercurrent illness consideration should be given to discontinuing treatment. Further treatment will depend upon the clinical situation and is at the discretion of the responsible clinician. Interruptions for < 1 week due to intercurrent illness or radiation toxicity will be recorded and treatment should be completed as planned.

4.4 HIGH DOSE ONCE DAILY THORACIC RADIOTHERAPY

4.4.1 Schedule

- **66 Gy** in **33 daily fractions** over a period of **45 days** (radiotherapy to start on a Monday)**,** 5 consecutive days a week
- The optimal overall treatment time should be 45 days. Up to 47 days is a protocol deviation and should be recorded, above 47 days is a protocol violation.
- using conformal radiotherapy and 4 to 10 MV photons emitted from linear accelerators.

- Thoracic radiotherapy will start on cycle 1 day 22, if possible concurrently with the second cycle of chemotherapy (within 24 hours of day 1, cycle 2 of PE).

4.4.2 Normal tissue constraints

To reduce late damages to the normal tissues the following rules will be applied

- Dose: **2 Gy** per fraction
- Maximum spinal cord dose will not exceed **48 Gy.** The spinal cord position must be identified throughout the PTV
- The percentage of lung minus PTV receiving more than 20 Gy will not exceed **35%** (V20=35%, based on dose-volume histograms). The mean lung dose will be recorded.
- The heart can receive the total dose (TD) to < 30% of its volume. For > 50% of cardiac volume, dose < 50% of TD is recommended

4.4.3 Treatment Delays

Every effort should be made to deliver the prescribed dose of radiotherapy in 45 days.

If unavoidable delays occur, that could increase the overall treatment time beyond 45 days, e.g. due to machine breakdown, compensation should if possible be made by one of the following mechanisms:

- giving two fractions on a subsequent day, with a minimum interval of six hours between fractions, ***or***
- treating on a weekend day, ***or***
- adjusting fraction size to deliver the total prescribed dose within 45 days;

However, fraction size should remain < 3 Gy.

If the radiation schedule is interrupted for more than 1 week due to intercurrent illness consideration should be given to discontinuing treatment. Further treatment will depend upon the clinical situation and is at the discretion of the responsible clinician. Interruptions for < 1 week due to intercurrent illness or radiation toxicity will be recorded and treatment should be completed as planned.

4.5 PROPHYLACTIC CRANIAL IRRADIATION

No later than 6 weeks after the last cycle of chemotherapy , patients without evidence of progressive disease on CXR or CT scan and with no clinical evidence of brain metastases will be given PCI.

Simulation is mandatory for whole brain irradiation. Patients should be treated in supine position. Immobilisation by individual masks or other means is recommended. Treatment will be delivered with megavoltage machines of energies ranging from 4-10 MV photons. Treatment with a single beam is not acceptable. Doses are specified at the mid-plane of two opposed lateral whole brain fields, prescribed to the isocenter. The dose and fractionation of PCI will be left to the discretion of each principal investigator to allow for variation in local practice.

4.6 CONCOMITANT MEDICATION

- Prophylactic antibiotics with fluoroquinolones (choice left to local investigator) are recommended to start on day 8 of cycle 1 chemotherapy and to continue for 7 days to minimise the risk of

neutropenic sepsis and respiratory infection. Consideration should be given to continuing prophylactic antibiotics after subsequent cycles if bronchial obstruction is present.

- Anti-emetics will be routinely used using a combination of a steroid and a 5-HT_3 antagonist.

- The use of GCSF is optional
    - GCSF will be prescribed, if clinically indicated, as per ASCO 2006 guidelines or in line with local policy / guidelines for treatment of febrile or prolonged neutropenia.
    - Secondary prophylaxis with GCSF is recommended
        - weight<70 kg: 300 µg days 5 to 12
        - weight>70 kg: 5 µg/kg days 5 to 12
    - GM-CSF will not be used.

- The use of erythropoietin for the treatment of anaemia will not be permitted

4.7 CRITERIA FOR STOPPING TREATMENT

Protocol treatment may be stopped in the following instances:

- Evidence of progressive disease according to RECIST criteria on CT scan (appendix 4)
- Unacceptable toxicity
- An early toxicity assessment (after 20 patients have completed treatment in each arm) for safety reasons will be carried out. Data will be reviewed by the TMG.
- Intercurrent illness which, in the clinician's opinion, would require discontinuation of protocol therapy.
- If subsequent histological/cytological review is contrary to the original diagnosis
- Patient's request

For patients with measurable disease, evidence of minor disease progression (stable disease by RECIST criteria) is not a requirement to stop treatment in an otherwise clinically and symptomatically stable patient.

4.8 THERAPY AFTER PROTOCOL TREATMENT HAS STOPPED

Treatment after relapse or on withdrawal of patient from protocol treatment is at the discretion of the participating clinician, as deemed appropriate according to the clinical situation. Palliative radiotherapy to areas outside the original radiotherapy treatment volume and second-line chemotherapy can be used according to local practice.

4.9 PATIENTS NOT MEETING THE RADIOTHERAPY PLANNING REQUIREMENTS

If the V20 lung, dose to the spinal cord and heart do not meet the protocol requirements, the treatment decision will be left to the local investigator, as some adjustments may be necessary regarding the radiotherapy treatments. We strongly recommend that the total dose of radiotherapy is dropped to meet the normal tissue constraints.

These patients, who would have been randomised prior to the first cycle of chemotherapy, will be included in the intention to treat analysis. A subanalysis will be done excluding patients with protocol violations.

5.0 PHARMACOVIGILANCE

5.1 DEFINITIONS

Adverse Event (AE): any untoward medical occurrence in a patient or clinical trial subject administered a medicinal product and which does not necessarily have a causal relationship with this treatment. *An AE can therefore be any unfavourable and unintended sign (including an abnormal laboratory finding), symptom, or disease temporally associated with the use of an investigational medicinal product (IMP), whether or not considered related to the IMP.*

Adverse Reaction (AR): all untoward and unintended responses to an IMP related to any dose administered. *All AE's judged by either the reporting investigator or the sponsor as having reasonable causal relationship to a medicinal product qualify as adverse reactions. The expression reasonable causal relationship means to convey in general that there is evidence or argument to suggest a causal relationship.*

Unexpected Adverse Reaction: an AR, the nature and severity of which is not consistent with the applicable product information (i.e. summary of product characteristics (SmPC) for cisplatin and etoposide which are authorised products*). When the outcome of the adverse reaction is not consistent with the applicable product information this adverse reaction should be considered as unexpected. Side effects documented in the SmPC, which occur in a more severe form that anticipated, are also considered to be unexpected.*

Serious Adverse Event (SAE)**:** for this trial an Adverse Event will be considered serious and require expedited reporting if it:

- results in **death** (within 30 days of last dose of chemotherapy or radiotherapy, irrespective of causality)
- is **life-threatening*** (and is considered related to study treatment)
- requires **hospitalisation****, or prolongation of existing hospitalisation (and is considered related to study treatment).
- results in persistent or significant **disability or incapacity,** (and is considered related to study treatment)
- is a **congenital anomaly** or **birth defect**

In addition the following will also be considered serious adverse events:

- **grade 3 and 4 radiation oesophagitis**
- **grade 3 and 4 radiation pneumonitis**

SERIOUS ADVERSE EVENTS REQUIRE REPORTING TO THE CHIEF INVESTIGATOR WITHIN 24 HOURS, SEE SECTION 5.3 FOR FURTHER INFORMATION ON REPORTING PROCEDURES.

Please note the following additional guidance with relation to definition of serious adverse events:

- Serious events not meeting the criteria outlined above: Medical judgement should be exercised in deciding whether an AE/AR is serious in other situations. Important AE/AR's that are not immediately

life-threatening or do not result in death or hospitalisation but may jeopardise the subject or may require intervention to prevent one of the other outcomes listed in the definition above, should also be considered serious.

- *Life-threatening events: The term 'life-threatening' in the definition of 'serious' refers to an event in which the patient was at risk of death at the time of the event; it does not refer to an event which hypothetically might have caused death if it were more severe.
- **Events requiring or prolonging hospitalisation: Hospitalisation is defined as an inpatient admission, regardless of length of stay, even if the hospitalisation is a precautionary measure for continued observation. Hospitalisations for a pre-existing condition, including elective procedures that have not worsened, do not constitute an SAE. Hospitalisations for palliative care, chemotherapy or radiotherapy, procedures or diagnostic procedures do not need to be reported.
- Progressive disease or death due to progressive SCLC does not need reporting as an SAE.

5.2 CAUSALITY

Most adverse events and adverse drug reactions, that occur in this trial, whether they are serious or not, will be expected treatment related toxicities due to either of the drugs used in this trial (see appendix 8 for list). The assignment of causality should be made using the definitions in the table below.

If any doubt about the causality exists the investigator should inform the trials centre who will notify the Chief Investigators. The pharma company/ies and/or other clinicians may be asked to advise in difficult cases.

In the case of discrepant views on causality between the investigator and others, all parties will discuss the case.

Relationship	Description	RESPONSE
Unrelated	There is no evidence of any causal relationship	Yes or No
Unlikely	There is little evidence to suggest there is a causal relationship (e.g. the event did not occur within a reasonable time after administration of the trial medication). There is another reasonable explanation for the event (e.g. the patient's clinical condition, other concomitant treatment).	Yes or No
Possible	There is some evidence to suggest a causal relationship (e.g. because the event occurs within a reasonable time after administration of the trial medication). However, the influence of other factors may have contributed to the event (e.g. the patient's clinical condition, other concomitant treatments).	Yes or No
Probable	There is evidence to suggest a causal relationship and the influence of other factors is unlikely.	Yes or No
Definitely	There is clear evidence to suggest a causal relationship and other possible contributing factors can be ruled out.	Yes or No
Not assessable	There is insufficient or incomplete evidence to make a clinical judgement of the causal relationship.	Yes or No

5.3 REPORTING PROCEDURES

All adverse events/reactions should be reported. Depending on the nature of the event the reporting procedures below should be followed. Any questions concerning adverse event reporting should be directed to the trials centre in the first instance.

5.3.1 Non-serious adverse events

All toxicities, whether expected or not, should be recorded in the toxicity section of the relevant case report form. These should be reported on the case report forms and sent to the Trial Centre within one month of the form being due. Progressive disease is not considered to be an adverse event.

5.3.2 Serious adverse events

All SAEs and SARs must be reported immediately by the local Investigator to the Chief Investigator. The site should:

- Either, complete the SAE case report form & send it immediately (within 24 hours or the next working day, preferably by fax 0161 446 8148), signed and dated to the trial centre together with relevant treatment forms and anonymised copies of all relevant investigations.
- Or, contact the Trial Centre by phone and then send the completed SAE form to the Trial Centre within the following 24 hours as above.

An SAE form should be completed for all SAEs (as defined in section 5.1). The form asks for nature of event, date of onset, severity, corrective therapies given, outcome and causality (i.e. unrelated, unlikely, possible, probably, definitely and not assessable). The responsible investigator should sign the causality of the event. Additional information should be sent within 5 days if the reaction has not resolved at the time of reporting.

The Trial Centre will notify the Main REC of all SUSARs occurring in the trial within 15 days of notification, and will provide the Main REC with an annual report of all SAEs. Investigators should report any SAEs and/or SARs as required by their Main Research Ethics Committee and/or Research & Development Office.

To report an SAE, an SAE form must be completed and returned **within 24 hours** of the clinician becoming aware of the event.

Fax: 0161 446 8148 for the attention of Dr C Faivre-Finn

Please note, all toxicities (whether or not they are SAEs) should also be recorded in the toxicity sections of the relevant case report forms.

6.0 ASSESSMENTS

6.1 ASSESSMENTS DURING TREATMENT

Please see section 4.0 for additional clinical visits required during treatment for each arm. During treatment the following information will be required and relevant details reported on case report forms (3-weekly):

- Clinical examination, performance status (see appendix 1), dyspnoea score (see appendix 3) and weight
- Haematology and biochemistry results
- Calculated creatinine clearance before each cycle of chemotherapy (see section 4.1.1)
- Details of any non cancer related treatment received
- Assessment of toxicity graded according to CTCAE v3.0 (see appendix 2)

6.2 ASSESSMENTS AT FOLLOW-UP

After completion of treatment, patients should be reviewed weekly until acute side effects have resolved then 3 monthly until 1 year, 6 monthly thereafter.

Follow up visits at more frequent intervals should be undertaken at the discretion of the participating clinician.

Assessments to include:

- Clinical examination, performance status (see appendix 1), dyspnoea score (see appendix 3) and weight
- Assessment of any continuing or new toxicity (see appendix 2)
- Assessment of disease status
- Assessment of response by a contrasted CT scan thorax and abdomen within 4 weeks of cycle 4 (even if 6 cycles are given), 6 months after randomisation and 12 months after randomisation.
- Details of any additional cancer treatment received
- Late toxicity (CTCAE v3.0)

Participating sites should make all necessary efforts to ensure that follow-up information is obtained for all participants e.g. this may include methods such as tracking the patient through a national cancer registry, GP records network etc if contact is lost.

6.3 COST EFFECTIVENESS

The radiotherapy schedules being compared may have different adverse event profiles and efficacy, and that might translate into different costs, effects and cost-effectiveness.
Therefore the following data will be collected routinely on clinical report forms:

- details of protocol medications
- use of antibiotics, blood & platelet transfusions, GCSF
- management of serious adverse events
- hospital admission episodes

6.4 Summary of protocol assessment visits

PROTOCOL ACTIVITIES	SCREEN	CYCLE 1 TREATMENT	ADDITIONAL CYCLES			END OF TREATMENT (within 4 weeks of last cycle)	FOLLOW-UP[f]
			Chemotherapy – both arms (3 weekly)	Once Daily XRT Arm (45 days)	Twice Daily XRT Arm (19 days)		
**Check eligibility**	X						
**Informed Consent**	X						
**Medical History**	X						
**Physical Examination**	X	X	X[a]			X	X
**Pulmonary function tests**	X						
**Bronchoscopy or CT guided biopsies**	X						
**Pleural Aspiration / cytology**	X[d]						
**CT Planning Scan**	X						
**Randomisation**	X						
**Haematology**	X	X	X[a]			X	
**Biochemistry**	X	X	X[a]			X	
**Creatinine Clearance**	X	X	X[a]				
**CXR**		X	Prior to cycle 3,5				X
**ECG**	X						
**CT Scan– Chest/Upper Abdo**	X[e]					After cycle 4	X (6 months & 12 months after randomisation, and on relapse)
**CT/MR Scan-Brain**	X [e]						
**Chemotherapy**		X	X[b]				
**Radiotherapy**				X	X		
**PCI (if indicated)**						X[c]	
**Adverse Events/Toxicities**	X	X (baseline)	X	X	X	X	X
**Radiation Toxicities**			X	X	X	X	X
**Research blood specimen**		X (days 1 & 22)				X	
**Tumour sample**		X					X (relapse)

[a] Measured as per local policy but at a minimum of 3-weekly
[b] Cycle 1 of chemotherapy to start within 2 weeks of randomisation and assessment of clinical fitness
[c] PCI- to be performed a maximum of 6 weeks after chemotherapy completion
[d] Pleural aspiration/ cytology- to be performed if possible in patients with pleural effusion
[e] Screening chest/ upper abdo CT & screening brain CT/MR- to be performed within 4 weeks prior to randomisation
[f] Follow-up visit scheduling- weekly review until resolution of acute side effects, then 3-monthly until 1 year post randomisation, 6 monthly thereafter (or more frequently at discretion of the investigator)

6.5 STUDY FORMS

**Eligibility checklist**	To be completed at the time of randomisation, prior to phoning the trials office.	**Form Pre-a**
**Pre-treatment form**	To be completed at baseline prior to cycle 1	**Form Pre-b**
**Tumour assessment form**	To be completed at baseline prior to cycle 1	**Form Pre-c**
**Treatment form**	To be completed on day 1 of each cycle. Data collection includes: protocol treatment(s) received, toxicity, reasons for reduction/delay/omission, performance status<br><br>***Complete forms for all cycles (1-4 or 6)***	**Form A**
**Toxicity form**	To be completed at the end of each cycle given, prior to next cycle and 30 days after completion of last cycle of chemotherapy<br><br>***Complete forms post cycle to capture all data***	**Form B**
**Radiotherapy worksheet**	To be completed during and after completion of radiotherapy	**Form C**
**Post treatment form**	To be completed 30 days after last cycle of chemotherapy	**Form Post treatment**
**Follow-up forms**	To be completed at each follow-up visit 3 months after post cycle 4(6) visit	**Form FU**
**Serious Adverse Event form**	To be completed for all serious adverse events	**Form SAE**
**Prog/Rel/Death Form**	To be completed on death of patient.	**Form Prog/Rel/Death**

Essential Forms

**Pre randomisation**	**Pre treatment**	**Pre Cycle 1-6**	**Post Cycle 1-6**	**Post cycle 4 (6)**	**3, 6, 9, 12 months (then 6 monthly until death)**
Pre-a	Pre-b<br>Pre-c	A	B<br>C (2– BD)<br>C (4- OD)	Post treatment	FU

Additional Forms (if required)

**Any serious events**	**Progression**	**Relapse**	**Death**
SAE-SUSAR	Prog/Rel/Death	Prog/Rel/Death	Prog/Rel/Death

6.6 DATA HANDLING

A copy of all trial forms will be returned to the trials centre for statistical analysis. The remaining copy is to be retained at the local centre.

All forms will be scanned and entered into a study defined database for which some consistency checking will be programmed in. Data managers will check for missing and invalid data using SQL queries and statistical programs. Any queries will be highlighted on the forms and returned to the centres for correction. The data will at all times be password protected.
The CRF forms will be due as described in section 6.5.
On completion of the study the data will be written onto CD and archived in a safe and secure location within the department.
Paper copies of the CRF's will be retained on site for at least fifteen years following the last patient entered or if all are deceased may be archived off site. All paper data will be destroyed after fifteen years on the approval of the chief investigator.

The trials centre staff will be in regular contact with local centre personnel to check on progress and to help with any queries that may arise. Incoming forms will be checked for completeness, consistency, timeliness and compliance with the protocol. Centres may be withdrawn from further recruitment in the event of serious and persistent non-compliance.

7.0 STATISTICAL CONSIDERATIONS

7.1 Sample size calculation

It is considered that a survival benefit of 12% at 2 years (in favour of once-daily radiotherapy) would be clinically significant. Using Freedman's sample size calculation based on a 2-arm trial, with a 5% significance level, 2-sided test, 80% power, and hazard ratio of 0.70, a 12% overall survival benefit at 2 years from 44% with the control arm to 56% in the experimental arm, requires a total of 506 patients. The number of deaths required is 247. An additional 5% will be added to allow for ineligible patients giving a total of 532 patients required.

7.1.1 Primary end-point

- Overall survival

Secondary end points

- Local progression-free survival
- Metastasis-free survival
- CTCAE v3.0 toxicity
- Chemotherapy dose intensity
- Radiotherapy dose intensity

7.2 General Considerations

For data analyses, patient data will be grouped by treatment arm according to the treatment assignments made via the Christie Hospital randomization line(described in Section 3.3).

7.2.1 Interim analyses

The only planned interim analyses will be performed for the independent Data Monitoring Committee (see section 9.0). A report will be given to the IDMC approximately 12 months after the first patient is randomised and then when requested by the IDMC.

7.3 Qualifications for Efficacy Analysis

7.3.1 Qualifications for Analysis of Time-to-Event and Other Efficacy

Parameters

The following are the qualifications for analysis of time-to-event efficacy parameters:

• All randomized patients will be included in the analysis of OS and Local PFS (ITT).

Overall survival and local progression-free survival.

Overall Survival is the time between date of randomisation and date of death of any cause. Survivors will be censored on the last date known to be alive. ***Local progression-free survival (local control)*** will be calculated from the date of randomisation to the date of first clinical evidence of progressive disease at the primary site, or death.
Kaplan-Meier curves (Kaplan and Meier 1958) will be drawn for each treatment group. Overall survival and local progression-free survival will be compared using the Mantel-Cox version of the log rank test.

• All randomized patients treated with at least one study dose of cisplatin and etoposide will be included in the comparison of proportions of grade 3 and 4 toxicities.

Toxicity will be assessed according to NCI Common Terminology Criteria for Adverse Events v 3.0 (see appendix 2). The proportions of patients experiencing a grade of 3 or above acute toxicity, including acute radiation morbidity, or late radiation morbidity will be compared between the treatment groups using Chi-squared and Fisher Exact tests. Acute toxicity will be defined as toxicities occurring from commencement of treatment to 3 months after completion, late toxicity will be defined as toxicities occurring between 3 months and 2 years after completion of treatment.

7.3.2 Qualifications for Analysis of Tumour Response

All patients meeting the following criteria will be evaluated for tumour response and included in the analysis of tumour response rates:

• treatment with at least one dose of cisplatin and etoposide.

Response will be assessed according to RECIST criteria (appendix 4) and the proportion of patients in each treatment group whose best response up to, approximately 28 days post cycle 4 or, if stopped prior to cycle 4, approximately 28 days after last chemotherapy cycle given, from randomisation is complete or partial will be compared using Chi-squared and Fisher Exact tests.

7.4 Qualifications for Safety Analysis

Safety analyses will be performed for all randomized patients treated with at least one dose of study drug. Adverse effects will be summarized and compared between the two arms.

7.5 Qualifications for Dose Intensity Analysis

The relative chemotherapy and radiotherapy dose intensity will be summarised by calculating the median, standard deviation, interquartile range and range for patients in each randomised treatment group. The RDI will be compared between the treatment groups by using the Wilcoxon Rank Sum test.

Chemotherapy RDI:
Relative dose intensity (RDI) is defined as the dose intensity achieved by a patient relative to a schedule that would have given the intended PE chemotherapy on a fixed 3-weekly schedule. This is given a proportion of the fixed 3 weekly schedule and will be calculated assuming that each component of the chemotherapy schedule is equipotent. The duration of the chemotherapy for this calculation will run from the first day of chemotherapy to the last dose of the final cycle. The RDI will therefore be calculated as:

$$\frac{1}{2}\left(\frac{\text{Platinum}_{AT}}{\text{Platinum}_{PT}}+\frac{\text{Etoposide}_{AT}}{\text{Etoposide}_{PT}}\right).\left(\frac{\text{Time}_{PT}}{\text{Time}_{AT}}\right)$$

where Platinum_{AT}, Etoposide_{AT}, are the actual total received doses and Platinum_{PT}, Etoposide_{PT} are the respective planned total doses.

Radiotherapy RDI:
Relative dose intensity (RDI) is defined as the dose intensity achieved by a patient relative to a schedule that would have given the intended radiotherapy on a fixed 45 day schedule for OD and 19 day schedule for BD. The duration of the radiotherapy for this calculation will run from the first day of radiotherapy to the last day of the radiotherapy. The RDI will therefore be calculated as:

$$\left(\frac{\text{totaldose}_{AT}}{\text{totaldose}_{PT}}\right)\cdot\left(\frac{\text{time}_{PT}}{\text{time}_{AT}}\right)$$

where totaldose_{AT}, is the actual total dose received and totaldose_{PT} is the respective planned total dose received.

7.6 Patient Disposition
A detailed description of patient disposition will include a summary of the following:
- all patients entered and enrolled: overall, by treatment arm, and by country
- reasons for patients entered, but not enrolled
- all enrolled patients treated with study drug, by treatment arm
- reasons patients enrolled, but not treated with study drug
- reasons patients discontinued study drug treatment
- all important protocol violations.

7.7 Patient Characteristics
Patient characteristics will include a summary of the following:
- patient demographics
- baseline disease characteristics
- number of chemotherapy cycles given

Other patient characteristics will be summarized as deemed appropriate.

7.8 Concomitant Therapy
Use of antibiotics and growth factors will be summarized for all randomized patients per treatment arm.

7.9 Subsequent Therapy
Subsequent treatment will be summarized for all randomized patients per treatment arm.

7.10 Treatment Compliance
Dose omissions, reductions, and delays will be summarized for all randomized patients per treatment arm.

7.11 Health Outcomes
These analyses will be performed in an exploratory manner and hence will not introduce multiplicity to primary and other secondary efficacy analyses.
- details of protocol medications
- other anti-cancer treatments

- management of serious adverse events
- hospital admission episodes

8.0 INFORMED CONSENT, ETHICAL & REGULATORY CONSIDERATIONS

8.1 ETHICAL APPROVAL

This trial will adhere to the principles outlined in the International Conference on Harmonisation Good Clinical Practice (ICH GCP) guidelines. It will be conducted in compliance with the protocol, MRC GCP, the Data Protection Act (DPA G0027154) and other regulatory requirements, as appropriate.

Multicentre Research Ethics Committee (MREC) approval has been obtained for this trial, and Site Specific Assessments (SSAs) will be performed at participating centres. The trials centre will maintain contact with NRES and will submit any protocol amendments. The trials centre will forward any resulting documentation to local centres.

8.2 PATIENT INFORMED CONSENT

The local investigator is required to explain the nature and purpose of the trial to the patient prior to trial entry. A detailed patient information sheet and consent form will be given to the patient and written informed consent obtained before trial entry.

8.3 PROTOCOL COMPLIANCE

Christie CTU office staff will be in regular contact with local centre personnel to check on progress and to help with any queries that may arise. Incoming forms will be checked for completeness, consistency, timeliness and compliance with the protocol. Centres may be withdrawn from further recruitment in the event of serious and persistent non-compliance.

8.4 INDEMNITY & COMPENSATION

The CONVERT trial is an investigator led and designed trial co-ordinated by the Christie Clinical Trials Unit and MRC Clinical Trials Unit (Cancer Division). The principal investigator, local investigators and co-ordinating centres do not hold insurance against claims for compensation for injury caused by participation in a clinical trial and they cannot offer any indemnity. The Association of the British Pharmaceutical Industry (ABPI) guidelines will not apply. However, in terms of liability, NHS Trust and Non-Trust Hospitals have a duty of care to patients treated, whether or not the patient is taking part in a clinical trial. Therefore compensation is available in the event of clinical negligence being proven.

8.5 DATA PROTECTION

The trials centre will act to preserve patient confidentiality and will not disclose or reproduce any information by which patients could be identified. Data will be stored in a secure manner and our trials are registered in accordance with the Data Protection Act 1998 with the Data Protection Officer at the Christie Hospital.

8.6 DURATION OF TRIAL

The 'active' phase of the trial will finish when the last patient has completed their protocol treatment. The planned duration of the 'active' phase is 4 years. The 'follow up' phase of the trial will then begin; patients will be followed until death.

8.7 PUBLICATION POLICY

Data from all centres will be analysed together and published as soon as possible. Individual participants may not publish data concerning their patients that are directly relevant to questions posed by the trial until the Trial Management Group (TMG) has published its report. The TMG will form the basis of the Writing Committee and advise on the nature of publications.

All publications shall include a list of participants, and if there are named authors, these should include the principal investigator, clinical trial coordinator(s), and statistician(s) involved in the trial and contributors of more than 5% of participants. If there are no named authors then a writing committee will be identified.

9.0 INDEPENDENT DATA MONITORING AND ETHICS COMMITTEE (IDMC), INDEPENDENT TRIAL STEERING COMMITTEE (TSC) AND TRIAL MANAGEMENT GROUP (TMG)

The data will be reviewed (at least annually) by an IDMC, consisting of at least two clinicians not entering patients into the trial and an independent statistician. The IDMC will be asked to recommend whether the accumulated data from the trial, together with results from other relevant trials, justifies continuing recruitment of further patients. A decision to discontinue recruitment, in all patients or in selected subgroups will be made only if the result is likely to convince a broad range of clinicians including participants in the trial and the general clinical community. If a decision is made to continue, the IDMC will advise on the frequency of future reviews of the data on the basis of accrual and event rates. The IDMC will make confidential recommendations to the TSC.

The role of the TSC is to act on behalf of the funder, to provide overall supervision for the trial, to ensure that it is conducted in accordance with GCP, and to provide advice through its independent Chairman. This independent committee will review the recommendations from the IDMC and will decide on continuing or stopping the trial, or modifying the protocol.

The Trial Management Group coordinates and manages the trial's day-to-day activities. The TMG is comprised of health professionals as listed on page 3 of this protocol.

10.0 REFERENCES

1. Murray N, Coy P, Pater JL, et al.: Importance of timing for thoracic irradiation in the combined modality treatment of limited-stage small-cell lung cancer. Journal of Clinical Oncology 11(2): 336-344, 1993.
2. Johnson BE, Bridges JD, Sobczeck M, et al. Patients with limited-stage small-cell lung cancer treated with concurrent twice-daily chest radiotherapy and etoposide/cisplatin followed by cyclophosphamide, doxorubicin, and vincristine. Journal of Clinical Oncology 14(3): 806-813, 1996.
3. Turrisi AT III, Kim K, Blum R, et al.: Twice-daily compared with once-daily thoracic radiotherapy in limited small-cell lung cancer treated concurrently with cisplatin and etoposide. New England Journal of Medicine 340(4): 265-271, 1999.
4. Pignon JP, Arriagada R, Ihde DC, et al.: A meta-analysis of thoracic radiotherapy for small-cell lung cancer. N Engl J Med 1999, 340, 265-271
5. Warde P, Payne D: Does thoracic irradiation improve survival and local control in limited-stage small-cell carcinoma of the lung? A meta-analysis. J Clin Oncol 10(6): 890-895, 1992.
6. Thatcher N, Qian W, Clark P, et al. Ifosfamide, carboplatin, and etoposide with midcycle vincristine versus standard chemotherapy in patients with small-cell lung cancer and good performance status: clinical and quality-of-life results of the British Medical Research Council multicenter randomized LU21 trial. J Clin Oncol 2005; 23:8371-79
7. Takada M, Fukuoka M, Kawahara M, et al. Phase III Study of Concurrent Versus Sequential Thoracic Radiotherapy in Combination with Cisplatin and Etoposide for Limited-Stage Small Cell Lung Cancer : The Japan Clinical Oncology Group Study 9104. J Clin Oncol 20 (14): 3054-60, 2002.
8. Jeremic B, Shibamoto Y, Acimovic L et al. Initial versus delayed accelerated hyperfractionated radiation therapy and concurrent chemotherapy in limited small-cell lung cancer: a randomized study. J Clin Oncol 1997 15 (3): 893-900.
9. Gregor A, Drings P, Burghouts J et al.Randomised trial or alterning versus sequential radiotherapy/chemotherapy in limited disease patients with small cell lung cancer: a EORTC Lung Cancer Cooperative Group study. J Clin Oncol 1997; 15: 2840-49.
10. Work E, Nielson OS, Bentzen SM et al. Randomised study of initial versus late chest irradiation combined with chemotherapy in limited stage small cell lung cancer. J Clin Oncol 1997 15: 3030-37.
11. Fried B, Morris D, Hensing T et al. Systematic review evaluating the timing of thoracic radiation therapy in combined modality therapy for limited stage small-cell lung cancer. J Clin Oncol 2004, 22, 4785-93
12. Pijls-Johannesma MCG et al. Early versus late chest radiotherapy for limited stage small cell lung cancer. The Cochrane Database of Systematic Reviews; Issue 4. Art. No: CD004700, 2004.
13. Carney DN, Mitchel BJ, Kinsella TJ. In vitro radiation and chemotherapy sensitivity of established cell lines of human small cell lung cancer and its large cell morphologic variants. Cancer Research 1983 43: 2806-11
14. Bonner JA, Sloan JA, Shanahan TG, Brooks BJ, Marks RS, Krook JE, Gerstner JB, Maksymiuk A, Levitt R, Mailliard JA, Tazelaar HD, Hillman S and Jett JR. Phase III comparison of twice-daily split-course irradiation versus once-daily irradiation for patients with limited stage small-cell lung carcinoma. J Clin Oncol 1999, 17, 2681-2691
15. Schild SE et al. Long-term results of a phase III trial comparing once-daily radiotherapy with twice-daily radiotherapy in limited-stage small-cell lung cancer. Int J Radiat Oncol Biol Phys 2004, 59, 943-51
16. Choi NC, Herndon III JE, Rosenman J, Carey RW, Chung CT, Bernard S, Leone L, Seagren S, Green M. Phase I study to determine the maximum-tolerated dose of radiation in standard daily and hyperfractionated-accelerated twice-daily radiation schedules with concurrent chemotherapy for limited-stage small-cell lung cancer. J Clin Oncol 1998, 16, 3528-3537
17. Komaki R, Swann RS, Ettinger DS, et al. Phase I study of thoracic radiation dose escalation with concurrent chemotherapy for patients with limited small-cell lung cancer: Report of Radiation Therapy Oncology Int J Radiat Oncol Biol Phys 2005 ;62(2):342-50
18. Mascaux C, Paesmans M, Berghmans T, Branle F, Lafitte JJ, Lemaitre F, Meert AP, Vermylen P and Sculier JP. A systematic review of the role of etoposide and cisplatin in the chemotherapy of small cell lung cancer with methodology assessment and meta-analysis. Lung Cancer 2000, 30, 23-36
19. Pujol J-L, Carestia L and Daures J-P. Is there a case for cisplatin in the treatment of small-cell lung cancer? A meta-analysis of randomised trials of a cisplatin-containing regimen versus a regimen without this alkylating agent. Br J Cancer 2000, 83, 8-15

20. Sundstrom S. Bremnes RM. Kaasa S. Aasebo U. Hatlevoll R. Dahle R. Boye N. Wang M. Vigander T. Vilsvik J. Skovlund E. Hannisdal E. Aamdal S. Norwegian Lung Cancer Study Group. Cisplatin and etoposide regimen is superior to cyclophosphamide, epirubicin, and vincristine regimen in small-cell lung cancer: results from a randomized phase III trial with 5 years' follow-up. J Clin Oncol 2002, 20, 4665-4672
21. Bremnes RM, Sundstrom S, Vilsvik J, Aasebo U; Norwegian Lung Cancer Study Group. Multicenter phase II trial of paclitaxel, cisplatin, and etoposide with concurrent radiation for limited-stage small-cell lung cancer. J Clin Oncol 2001, 19, 3532-3538
22. Thatcher N, Girling DJ, Hopwood P, Sambrook RJ, Qian W, Stephens RJ. Improving survival without reducing quality of life in small-cell lung cancer patients by increasing the dose-intensity of chemotherapy with granulocyte colony-stimulating factor support: results of a British Medical Research Council Multicenter Randomized Trial. Medical Research Council Lung Cancer Working Party. J Clin Oncol 2000, 18, 395-404

    Pujol JL, Douillard JY, Riviere A, Quoix E, Lagrange JL, Berthaud P, Bardonnet-Comte M, Polin V, Gautier V, Milleron B, Chomy F, Chomy P, Spaeth D, Le Chevalier T. Dose-intensity of a four-drug chemotherapy regimen with or without recombinant human granulocyte-macrophage colony-stimulating factor in extensive-stage small-cell lung cancer: a multicenter randomized phase III study. J Clin Oncol 1997, 15, 2082-2089
23. Johnson DH, Carbone DP. Increased dose-intensity in small-cell lung cancer: a failed strategy? J Clin Oncol 1999, 17, 2297-2299
24. Laurie SA, Logan D, Markman BR, Mackay JA, Evans WK. Practice guidelines for the role of combination chemotherapy in the initial management of limited-stage small cell lung cancer. Lung Cancer 2004, 43, 223-240
25. Senan, S., et al., Literature-based recommendations for treatment planning and execution in high-dose radiotherapy for lung cancer. Radiother Oncol, 2004. 71(2): 139-46.
26. ICRU (The International Commission on Radiation Units and Measurements) Report 50: Prescribing, Recording and Reporting Photon Beam Therapy. Landberg T, Chavandra J, Dobbs HJ, Hanks G, Johansson KA, Muller R, Purdy J. 1993. ICRU Publications, Bethesda.
27. Royston P, and Babiker A. A menu-driven facility for complex sample size calculation in randomized controlled trials with a survival or a binary outcome. Stata Journal 2002, 2, 151-163
28. Perry MC, Eaton WL, Propert KJ, et al. Chemotherapy with or without radiation therapy in limited small-cell carcinoma of the lung. N Engl J Med. 1987 Apr 9;316(15):912-8
29. Pijls-Johannesma MCG et al. Early versus late chest radiotherapy for limited stage small cell lung cancer. The Cochrane Database of Systematic Reviews; Issue 4. Art. No: CD004700, 2004.

11.0 GLOSSARY

**ADL**	Activities of Daily Living
**AE**	Adverse event
**ALT**	Alanine transaminase
**ANC**	Absolute Neutrophil Count
**AR**	Adverse reaction
**AST**	Aspartate aminotransferase
**ASTRO**	American Society for Therapeutic Radiology and Oncology
**AUC**	Area Under the Curve
**BD**	Twice daily
**CI**	Chief Investigator
**CR**	Complete Response
**CRF**	Case Report Form
**CT**	Computed Tomography
**CTU**	Clinical Trial Unit
**CXR**	Chest X-Ray
**D**	Day
**DLCO**	Gas Transfer Factor for Carbon Monoxide (Lung diffusion test)
**DPA**	Data Protection Act
**DVH**	Dose Volume Histograms
**ECG**	Electrocardiogram
**ECOG**	Eastern Cooperative Oncology Group
**EDTA**	Ethylene Diamine Tetra Acetate
**ESTRO**	European Society for Therapeutic Radiology and Oncology
**FBC**	Full Blood Count
**FEV1**	Forced Expiratory Volume in 1 second
**GCSF**	Granulocyte Colony Stimulating Factor
**GFR**	Glomerular Filtration Rate
**γGT**	Gamma Glutamiyl –Transferase
**GTV**	Gross Tumour Volume
**Gy**	Gray (unit of absorbed radiation dose)
**Hb**	Haemoglobin
**ICH GCP**	International Conference of Harmonisation - Good Clinical Practice
**ICRU**	The International Commission on Radiation Units and Measurements
**IDMC**	Independent Data Monitoring and Ethics Committee
**IMP**	Investigational Medicinal Product
**IPEM**	Institute of Physics and Engineering in Medicine
**IR(ME)R**	Ionising Radiations (Medical Exposure) Regulations
**ITT**	Intention-to-treat
**iv**	Intravenous
**KCL**	Potassium Chloride
**LD**	Longest Diameter
**LDH**	Lactic Dehydrogenase
**LFT**	Liver Function Tests
**LS**	Limited stage
**M**	Distant metastasis
**mmols**	Millimoles
**mmols/L**	Millimoles per litre
**μmol/L**	Micromoles per litre
**MHRA**	Medicines and Healthcare Related products Agency
**MRC**	Medical Research Council
**MV**	Megavolts
**N**	Nodal Involvement
**NCI CTCAE**	National Cancer Institute Common Terminology Criteria for Adverse Events
**NHS**	National Health Service

**NRES**	National Research Ethics Service
**OS**	Overall Survival
**PA**	Posteroanterior
**PCI**	Prophylactic cranial irradiation
**PD**	Progressive Disease
**PET**	Positron Emission Tomography
**PFS**	Progression-Free Survival
**PI**	Principal Investigator (named clinician at each centre)
**PO**	By mouth
**PR**	Partial Response
**PS**	Performance Status
**PTV**	Planning Target Volume
**PE**	Cisplatin and etoposide
**QL**	Quality of Life
**QALYS**	Quality-Adjusted Life-Years
**QART**	Quality Assurance in Radiotherapy
**REC**	Research Ethics Committee
**RECIST**	Response Evaluation Criteria in solid Tumours
**RT (#)**	Radiotherapy (fractions)
**SAE**	Severe Adverse Event
**SCLC**	Small cell lung cancer
**SD**	Stable Disease
**SmPC**	Summary of product characteristics
**SSA**	Site Specific Assessment
**SUSAR**	Suspected Unexpected Serious Adverse Events
**T**	Primary Tumour
**TD**	Total Dose
**TMG**	Trial Management Group
**TSC**	Trial Steering Committee
**U+E**	Urea + Electrolytes
**ULN**	Upper Limit of Normal
**V_{20} lung**	Volume of lung tissue receiving $\geq$ 20 Gy

APPENDIX 1

PERFORMANCE STATUS SCALE (ECOG)

GRADE

0 - Fully active, able to carry on all predisease performance without restriction (Karnofsky 90-100).

1 - Restricted in physically strenuous activity but ambulatory and able to carry out work of a light or sedentary nature, e.g. light house work, office work (Karnofsky 70-80).

2 - Ambulatory and capable of all self-care but unable to carry out any work activities. Up and about more than 50% of waking hours (Karnofsky 50-60).

3 - Capable of only limited self-care, confined to bed or chair more than 50% of waking hours (Karnofsky 30-40).

4 - Completely disabled. Cannot carry on any self-care. Totally confined to bed or chair (Karnofsky 10-20).

ref: - Oken, M.M., Creech, R.H., Tormey, D.C., Horton, J., Davis, T.E., McFadden, E.T., Carbone, P.P.: Toxicity And Response Criteria Of The Eastern Cooperative Oncology Group. Am J Clin Oncol 5:649-655, 1982.

ASSESSMENT OF PERFORMANCE STATUS (PS)

Performance Status (PS) is a critical prognostic factor in small cell lung cancer and an essential guide for therapy. Measurement of PS is an evaluation of the patient's activity levels at a specific point in time, and should not be influenced by either the patient's self perception based on previous levels of activity or subjective impressions of the patient's general appearance by the assessor. Specific questions by the clinician should include enquiry about how much time the patient has spent sitting or lying down during daylight hours in the past week (this can be expressed as a percentage or fraction of the day) and the most strenuous activity undertaken by the patient in the past few days. A list of activities performed in the previous three days can be useful. If the clinician is uncertain as to the patient's exact PS, then the patient should be asked to keep an activity diary for seven days and reassessed a week later.

APPENDIX 2

Selected NCI Common Terminology Criteria for Adverse Events v3.0 (revised 2003)

Full CTCAE available at http://ctep.cancer.gov (under Reporting Guidelines)

		Grade				
**Adverse Event**	**Short name**	**1**	**2**	**3**	**4**	**5**
**Allergy**						
Allergic reaction/ Hypersensitivty (including drug fever)	Allergic reaction	Transient flushing or rash, drug fever < 38 ° C	Rash, flushing urticaria, dyspnoea, drug fever ≥38 °C	Symptomatic bronchospasm, with or without urticaria; parenteral medication(s) indicated; allergy -related edema/angioedema; or hypotension	Anaphylaxis	Death
Drug fever should be coded under allergy/hypersensitivity. If fever is due to infection, code infection only						
**Blood/bone marrow**						
Haemoglobin (g/dl)	Haemoglobin	10 - <LLN	8.0 - 9.9	6.5 - 7.9	<6.5	Death
Neutrophils/ granulocytes (x 10^9/L)	Neutrophils	1.5 - <LLN	1.0 - 1.4	0.5 - 0.9	<0.5	Death
Leucocytes (total WBC) (x 10^9/L)	Leucocytes	3.0 - <LLN	2.0 - 2.9	1.0 - 1.9	<1.0	Death
Platelets - (x 10^9/L)	Platelets	75.0 - <LLN	50.0 - 74.9	25 - 49.9	<25.0	Death
**Cardiac**						
Cardiac General – Other (Specify)	Cardiac General – Other (Specify)	Mild	Moderate	Severe	Life threatening; disabling	Death
**Constitutional symptoms**						
Fatigue (asthenia, lethargy, malaise)	Fatigue	Mild fatigue over baseline	Moderate, some difficulties with ADL	Severe, interfering with ADL	Disabling	-
Fever (in absence of neutropenia ANC <1.0 x 10^9/L)	Fever	38.0 – 39.0 ^{0}C	>39.0 – 40.0 ^{0}C	> 40.0 ^{0}C for ≤ 24 hours	> 40.0 ^{0}C for > 24 hrs	Death
Fever felt to be caused by a drug should be coded as allergic reaction/hypersensitivity (including drug fever). If fever is due to infection, code infection only						

		Grade				
**Adverse Event**	**Short name**	**1**	**2**	**3**	**4**	**5**
Rigors/chills	Rigors/chills	Mild	Moderate, narcotics indicated	Severe or prolonged, not responsive to narcotics	-	-
**Dermatological/skin**						
Dry Skin	Dry Skin	Asymptomatic	Symptomatic, not interfering with ADL	Interfering with ADL	-	-
Hair loss/ Alopecia (scalp or body)	Alopecia	Thinning or patchy	Complete	-	-	-
Pruritus/itching	Pruritus/itching	Mild or localised	Intense or widespread	Intense or widespread and interfering with ADL	-	-
Rash/desquamation	Rash	Macular or papular eruption or erythema without associated symptoms	Macular or papular eruption or erythema with pruritis or other associated symptoms; localised desquamation or other lesions covering <50% of body surface area (BSA)	Severed, generalised erythroderma or macular, papular or vesicular eruption, desquamation covering ≥50% BSA	Generalised exfoliative, ulcerative, or bullous dermatitis	Death
Rash:<br>Dermatitis associated with radiation<br>*- Select:*<br>- Chemoradiation<br>- Radiation	Dermatitis<br>*- Select*	Faint erythema or dry desquamation	Moderate to brisk erythema; patchy moist desquamation, mostly confined to skin folds and creases; moderate oedema	Moist desquamation other than skin folds and creases; bleeding induced by minor trauma or abrasion	Skin necrosis or ulceration of full thickness dermis; spontaneous bleeding from involved site	Death
Rash:<br>Erythema multiforme (e.g. Stevens-Johnson syndrome, toxic epidermal necrolysis)	Erythema multiforme	-	Scattered, but not generalised eruption	Severe (e.g. generalised rash or painful stomatitis); IV fluids, tube feedings or TPN indicated	Life threatening, disabling	Death
Please note:		*Rash due to allergy is coded under Allergic reaction/ Hypersensitivty (including drug fever).*<br>*Rashes due to radiation, erythema multiforme, or the hand-foot skin reaction are coded separately. See full CTCAE*				
Dermatology/Skin<br>- Other (Specify)	Dermatology/Skin<br>- Other (Specify)	Mild	Moderate	Severe	Life threatening; disabling	Death

		Grade				
**Adverse Event**	**Short name**	**1**	**2**	**3**	**4**	**5**
**Gastro-Intestinal**						
Anorexia	Anorexia	Loss of appetite without alteration in eating habits	Oral intake altered without significant weight loss or malnutrition; oral nutritional supplements indicated	Associated with significant weight loss/malnutrition; IV fluids, tube feeding or TPN indicated	Life threatening consequences	Death
Constipation	Constipation	Occasional or intermittent symptoms; occasional use of stool softeners, laxatives, dietary modification, or enema	Persistent symptoms with regular use of laxatives or enemas indicated	Symptoms interfering with ADL; obstipation with manual evacuation indicated	Life-threatening consequences (eg obstruction, toxic megacolon)	Death
Diarrhoea	Diarrhoea	Increase of <4 stools/day over baseline; mild increase in ostomy output compared to baseline	Increase of 4-6 stools/day over baseline; IV fluids indicated < 24 hrs; moderate increase in ostomy output compared to baseline; not interfering with ADL	Increase of ≥ 7 stools/day over baseline; incontinence; IV fluids or parenteral support ≥ 24hrs; hospitalization; severe increase in ostomy output compared to baseline; interfering with ADL	Life-threatening consequences e.g. haemodynamic collapse	Death
Ileus, GI (functional obstruction of bowel, i.e. neuroconstipation)	Ileus	Asymptomatic, radiographic findings only	Symptomatic; altered GI function (e.g. altered dietary habits); IV fluids indicated <24hrs	Symptomatic and severely altered GI function; IV fluids, tube feeding, or TPN indicated ≥ 24hrs	Life-threatening consequences	Death
Mucositis/Stomatitis/ (clinical exam)<br>- *Select:*<br>- Anus<br>- Oesophagus<br>- Etc	Mucositis (clinical exam) – *Select*	Erythema of the mucosa	Patchy ulceration or pseudomembranes	Confluent ulceration/pseudomebranes, or bleeding with minor trauma	Tissue necrosis, significant spontaneous bleeding, or life threatening consequences	Death

		Grade				
**Adverse Event**	**Short name**	**1**	**2**	**3**	**4**	**5**
Nausea	Nausea	Loss of appetite without alteration in eating habits	Oral intake decreased without signs of weight loss/dehydration, malnutrition; IV fluids indicated < 24 hrs	Inadequate oral caloric or fluid intake; IV fluids, tube feeding , or TPN indicated ≥ 24 hrs	Life threatening consequences	Death
Oesophagitis	Oesophagitis	Asymptomatic pathologic, radiographic, or endoscopic findings only	Symptomatic; altered eating/swallowing (e.g. altered dietary habits, oral supplements); IV fluids indicate < 24 hrs	Symptomatic and severely altered eating/swallowing (e.g. indadequate oral intake); IV fluids tube feedings, or TPN indicated ≥ 24 hrs	Life-threatening consequences	Death
Vomiting	Vomiting	1 episode in 24 hrs	2-5 episodes in 24 hrs. IV fluids for <24 hrs	≥ 6 episodes in 24 hrs or IV fluids or TPN ≥ 24 hrs	Life threatening consequences	Death
**Infection**						
Febrile neutropenia (fever of unknown origin without clinically or microbiologically documented infection (ANC <1.0 x 10^9L, fever ≥ 38.5 ^{0}C)	Febrile neutropenia	-	-	Present	Life threatening consequences eg septic shock, hypotension, acidosis, necrosis	Death
Infection (documented clinically or microbiologically) with Grade 3 or 4 neutrophils (ANC <1.0 x 10^9L) - *Select*	Infection (documented clinically) - *Select*	-	Localized, local intervention indicated	IV antibiotic, antifungal or antiviral interventional or operative intervention	Life threatening consequences eg septic shock, hypotension, acidosis, necrosis	Death
Infection with normal ANC or Grade 1 or 2 neutrophils - *Select*	Infection with normal ANC - *Select*	-	Localized, local intervention indicated	IV antibiotic, antifungal or antiviral intervention or operative intervention	Life threatening consequences eg septic shock, hypotension, acidosis, necrosis	Death
Please refer to the end of the CATEGORY on the full CTCAE ver 3.0 for list of AEs to select						

		Grade				
**Adverse Event**	**Short name**	**1**	**2**	**3**	**4**	**5**
**Muscolosketetal/soft tissue**						
Muscolosketetal/soft tissue - Other (Specify)	Muscolosketetal - Other (Specify)	Mild	Moderate	Severe	Life Threatening, Disabling	Death
**Neurological**						
Dizziness	Dizziness	With head movements or nystagmus only, not interfering with function	Interfering with function, but not with ADL	Interfering with ADL	Disabling	-
Myelitis	Myelitis	Asymptomatic, mild signs eg Babinski's or L'Hermitte's sign	Weakenss or sensory loss not interfering with ADL	Weakenss or sensory loss interfering with ADL	Disabling	Death
Neuropathy: sensory	Neuropathy: sensory	Asymptomatic; loss of deep tendon reflexes or parasthesia (including tingling) not interfering with function	Sensory alteration of paresthesia (including tingling), interfering with function, but not interfering but not ADL	Sensory alteration or parasthesia interfering with ADL	Disabling	Death
Somnolence/ Depressed level of consciousness	Somnolence	-	Somnolence or sedation interfering with function, but not with ADL	Obtundation or stupor, difficult to arouse, interfering with ADL	Coma	Death
**Pain**-*select*						
Pain<br>- *Select*	- Pulmonary/ Upper respiratory<br>- *Select*<br>- Chest wall<br>- Chest/thorax NOS	Mild pain not interfering with function	Moderate pain; pain or analgesics interfering with function, but not interfering with ADL	Severe pain; pain or analgesics severely interfering with ADL	Disabling	-

		Grade				
**Adverse Event**	**Short name**	**1**	**2**	**3**	**4**	**5**
**Pulmonary/ upper respiratory**						
Pneumonitis/pulmonary infiltrates	Pneumonitis	Asymptomatic, radiographic findings only	Symptomatic, not interfering with ADL	Symptomatic, interfering with ADL; O_2 indicated	Life-threatening; ventilatory support indicated	Death
Pneumonitis fibrosis	Pneumonitis fibrosis	Minimal radiographic findings (or patchy bibasilar changes) with estimated radiographic proportion of the total lung volume that is fibrotic of <25 %	Patchy or bi-basilar changes with estimated radiographic proportion of the total lung volume that is fibrotic of 25-<50 %	Dense or widespread infiltrates/consolidation with estimated radiographic proportion of the total lung volume that is fibrotic of 50 - <75 %	Estimated radiographic proportion of the total lung volume that is fibrotic is ≥ 75 %; honeycombing	Death
Cough	Cough	Symptomatic, non-narcotic medication only indicated	Symptomatic and narcotic medication indicated	Symptomatic and significantly interfering with sleep or ADL	–	–
Dyspnea (shortness of breath)	Dyspnea	Dyspnea on exertion, but can walk 1 flight of stairs without stopping	Dyspnea on exertion but unable to walk 1 flight of stairs or 1 city block (0.1km) without stopping	Dyspnea with ADL	Dyspnea at rest; intubation/ ventilator indicated	Death
**Renal**						
Creatinine	Creatinine	>ULN – 1.5 x ULN	>1.5 – 3.0 x ULN	> 3.0 – 6.0 ULN	>6.0 x ULN	Death
**Vascular**						
Thrombosis/thrombus/ embolism	Thrombosis/thrombus/ embolism	-	Deep vein thrombosis or cardiac thrombosis; intervention (e.g. anticoagulation, lysis) not indicated	Deep vein thrombosis, or cardiac thrombosis; intervention (e.g. anticoagulation, lysis) indicated	Embolic event including pulmonary embolism or life-threatening thrombus	Death

APPENDIX 3

MRC DYSPNOEA SCORE

GRADE

0 - Climbs hills or stairs without dyspnoea

1 - Walks any distance on flat without dyspnoea

2 - Walks over 100 yards without dyspnoea

3 - Dyspnoea on walking 100 yards or less

4 - Dyspnoea on mild exertion, eg undressing

5- Dyspnoea at rest

Ref: MRC Lung Cancer Working Party. 'A randomised trial of three or six courses of etoposide cyclophosphamide methotrexate and vincristine or six courses of etoposide and ifosfamide in small cell lung cancer (SCLC) 1: survival and prognostic factors. (1993) BJC 68, 1150-56

APPENDIX 4

RESPONSE CRITERIA (RECIST)

Baseline documentation of "Target" and "Non-Target" lesions

- All measurable lesions up to a maximum of five lesions per organ and 10 lesions in total, representative of all involved organs should be identified as ***target lesions*** and recorded and measured at baseline.
- Target lesions should be selected on the basis of their size (lesions with the longest diameter) and their suitability for accurate repeated measurements (either by imaging techniques or clinically).
- A sum of the longest diameter (LD) for *all target lesions* will be calculated and reported as the baseline sum LD. The baseline sum LD will be used as reference by which to characterize the objective tumor.
- All other lesions (or sites of disease) should be identified as ***non-target lesions*** and should also be recorded at baseline. Measurements of these lesions are not required, but the presence or absence of each should be noted throughout follow-up.

Response Criteria

	**Evaluation of target lesions**
**Complete Response (CR):**	Disappearance of all target lesions
**Partial Response (PR):**	At least a 30% decrease in the sum of the LD of target lesions, taking as reference the baseline sum LD
**Progressive Disease (PD):**	At least a 20% increase in the sum of the LD of target lesions, taking as reference the smallest sum LD recorded since the treatment started or the appearance of one or more new lesions
**Stable Disease (SD):**	Neither sufficient shrinkage to qualify for PR nor sufficient increase to qualify for PD, taking as reference the smallest sum LD since the treatment started
	**Evaluation of non-target lesions**
**Complete Response (CR):**	Disappearance of all non-target lesions and normalization of tumor marker level
**Incomplete Response/ Stable Disease (SD):**	Persistence of one or more non-target lesion(s) or/and maintenance of tumor marker level above the normal limits
**Progressive Disease (PD):**	Appearance of one or more new lesions and/or unequivocal progression of existing non-target lesions (1)

(1) Although a clear progression of "non target" lesions only is exceptional, in such circumstances, the opinion of the treating physician should prevail and the progression status should be confirmed later on by the review panel (or study chair).

Evaluation of best overall response

The best overall response is the best response recorded from the start of the treatment until disease progression/recurrence (taking as reference for PD the smallest measurements recorded since the treatment started). In general, the patient's best response assignment will depend on the achievement of both measurement and confirmation criteria

Target lesions	Non-Target lesions	Evaluation of non-target lesions	Overall response
CR	CR	No	CR
CR	Incomplete response/SD	No	PR
PR	Non-PD	No	PR
SD	Non-PD	No	SD
PD	Any	Yes or No	PD
Any	PD	Yes or No	PD
Any	Any	Yes	PD

- Patients with a global deterioration of health status requiring discontinuation of treatment without objective evidence of disease progression at that time should be classified as having "symptomatic deterioration". Every effort should be made to document the objective progression even after discontinuation of treatment.
- In some circumstances it may be difficult to distinguish residual disease from normal tissue. When the evaluation of complete response depends on this determination, it is recommended that the residual lesion be investigated (fine needle aspirate/biopsy) to confirm the complete response status.

Ref: Therasse et al 'New guidelines to evaluate the response to treatment in solid tumours' (2000) JNCI. 92 (3); 205-16

A quick reference guide is given at: **www3.cancer.gov/bip/RECIST.htm**

APPENDIX 5

COCKCROFT & GAULT FORMULA

If creatinine measured in µmol/l:

Males: $$\frac{1.23 \times (140 - \text{age}) \times \text{weight(kg)}}{\text{serum creatinine (µmol/l)}}$$

Females: $$\frac{1.05 \times (140 - \text{age}) \times \text{weight(kg)}}{\text{serum creatinine (µmol/l)}}$$

If creatinine measured in mg/%

Males: $$\frac{(140 - \text{age}) \times \text{wt (kg)}}{72 \times \text{serum creatinine}}$$

Females: $$\frac{(140 - \text{age}) \times \text{wt (kg)} \times 0.85}{72 \times \text{serum creatinine}}$$

APPENDIX 6

SUGGESTED SCHEME FOR ADMINISTRATION OF CHEMOTHERAPY CYCLES

Etoposide and Cisplatin every 21 days for 4-6 cycles

Etoposide

100 mg/m^2 days 1-3 BSA daily administered as an intravenous infusion over 1 hour every 3 weeks

Cisplatin

75 mg/m^2 day 1 or 25 mg/m^2 days 1-3 BSA administered as an intravenous infusion over 2 hours every 3 weeks

OPTION 1: Cisplatin given on day 1 only
Day 1

Administer anti-emetics according to local guidelines

Time	Drug	Fluid
0.00	-	1 litre 0.9% sodium chloride over 2 hours with 20 mmol of KCl
When urine output $\geq$ 100ml/hour:		
2.00	cisplatin 75mg/m^2	Given in 1 litre normal saline with 1g MgCl 20mmol over 2 hours
4.00	Etoposide 100mg/m^2	1 litre 0.9% sodium chloride over 1 hour
5.30	Post – cisplatin hydration	1 litre iv normal saline with KCl 20mmol and magnesium sulphate 10 mmol over 2 hours

Maintain oral intake of 1-2 litres of fluid for 6 hours after iv fluids discontinued.

Days 2 and 3

Administer anti-emetics according to local guidelines.

0.00	Etoposide 100 mg/m^2	1 litre 0.9% sodium chloride over 1 hour

OPTION 2: Cisplatin given day 1 to 3

Days 1-3

Time	Drug	Fluid
0.00	Cisplatin 25 mg/m2 d1-3	500ml normal saline over 1 hour
1.00	Etoposide 100 mg/m2 d1-3	500ml normal saline over 1 hour

Patients to have an oral intake of 2 litres of fluid days 1 to 3

APPENDIX 7

CHEMOTHERAPY AGENTS - safety profiles

Etoposide - http://emc.medicines.org.uk (as per SmPC)

Safety profile
The main toxicities associated with etoposide are:

Gastrointestinal toxicity: Nausea and vomiting are the major gastro-intestinal toxicities. The severity of such nausea and vomiting is generally mild to moderate with treatment discontinuation required in 1% of patients. Diarrhoea, anorexia and mucositis may occur. Constipation and swallowing disorder have been observed rarely.

Haematoxicity: Myelosuppression is dose limiting, with granulocyte nadirs occurring 5 to 15 days after drug administration and platelet nadirs occurring 9 to 16 days after drug administration. Bone marrow recovery is usually complete by day 21, and no cumulative toxicity has been reported.

Allergic reactions: Seldom hypersensitivity reactions caused by benzyl alcohol may occur. Anaphylactic-like reactions characterised by flushing, tachycardia, bronchospasm, and hypotension have been reported (incidence 0.7-2%), also apnoea followed by spontaneous recurrence of breathing after withdrawal of etoposide infusion, increase in blood pressure. The reactions can be managed by cessation of the infusion and administration of pressor agents, corticosteroids, antihistamines and/or volume expanders as appropriate.

Hypotension: Transient hypotension following rapid intravenous administration has been reported in 1% to 2% of patients. It has not been associated with cardiac toxicity or electrocardiographic changes. To prevent this rare occurrence, it is recommended that etoposide is administered by slow intravenous infusion over a 30- to 60-minute period. If hypotension occurs, it usually responds to supportive therapy after cessation of the administration. When restarting the infusion, a slower administration rate should be used.

Other toxicities: Reversible alopecia, sometimes progressing to total baldness was observed in up to 70% of patients. The following adverse reactions have been infrequently reported:
peripheral neuropathy, paresthesia, increased liver function tests with high doses, radiation "recall" dermatitis, hand-foot syndrome, central nervous effects (fatigue, drowsiness) 0-3%, hyperuricemia, taste impairment, fever, rash, urticaria, skin discoloration, pruritus, abdominal pain. The following adverse events have been reported after administration of etoposide (a causal relationship has not been established): Stevens-Johnson syndrome, rhythm disorders, myocardial infarction, reversible loss of vision.

Cisplatin - http://emc.medicines.org.uk (as per SmPC)

Safety profile
The main toxicities associated with cisplatin are:

Nephrotoxicity: Renal toxicity has been noted in about one third of patients given a single dose of cisplatin when prior hydration has not been employed. It is first noted during the second week after a dose and is manifested by elevations in plasma urea and serum creatinine, serum uric acid and/or decrease in creatinine clearance.

Renal toxicity becomes more prolonged and severe with repeated courses of the drug. Renal function must return to acceptable levels before another dose of cisplatin can be given.

Renal function impairment has been associated with renal tubular damage. The administration of cisplatin using a 6-8 hour infusion with intravenous hydration and mannitol has been used to reduce nephrotoxicity. However renal toxicity still can occur after utilisation of these procedures.

Gastrointestinal toxicity: Nausea and vomiting occur in the majority of patients, usually starting within 1 hour of treatment and lasting up to 24 hours. Anorexia, nausea and occasional vomiting may persist for up to a week.

Ocular toxicity: There have been reports of optic neuritis, papilloedema and cerebral blindness following treatment with cisplatin. Improvement and/or total recovery usually occurs following immediate discontinuation. Blurred vision and altered colour perception have been reported after the use of regimens with higher doses of cisplatin or greater dose frequencies than those recommended.

Ototoxicity: Ototoxicity has occurred in up to 31% of patients treated with a single dose of cisplatin 50 mg/m^2. Ototoxicity may be more severe in children and more frequent and severe with repeated doses.

Careful monitoring should be performed prior to initiation of therapy and prior to subsequent doses of cisplatin.

Unilateral or bilateral tinnitus, which is usually reversible, and/or hearing loss in the high frequency range may occur.

The overall incidence of audiogram abnormalities is 24%, but large variations exist. These abnormalities usually appear within 4 days after drug administration and consist of at least a 15 decibel loss in pure tone threshold. The damage seems to be cumulative and is not reversible. The audiogram abnormalities are most common in the 4000-8000 Hz frequencies.

Haematoxicity: Myelosuppression is observed in about 30% of patients treated with cisplatin. Leucopenia and thrombocytopenia are more pronounced at higher doses. The nadirs in circulating platelets and leucocytes generally occur between days 18-23 (range 7.3 to 45) with most patients recovering by day 39 (range 13 to 62). Leucopenia and thrombocytopenia are more pronounced at doses greater than 50 mg/m^2. Anaemia (decreases of greater than 2 g% haemoglobin) occurs at approximately the same frequency, but generally with a later onset than leucopenia and thrombocytopenia. Subsequent courses of cisplatin should not be instituted until platelets are present at levels greater than 100,000/mm^2 and white cells greater than 4,000/mm^2. A high incidence of severe anaemia (range 9-40%) requiring transfusion of packed red cells has been observed in patients receiving combination chemotherapy including cisplatin.

Anaphylaxis: Reactions possibly secondary to cisplatin therapy have been occasionally reported in patients who were previously exposed to cisplatin. Patients who are particularly at risk are those with a prior history or family history of atopy. Facial oedema, wheezing, tachycardia, hypotension and skin rashes of urticarial non-specific maculopapular type can occur within a few minutes of administration. Serious reactions seem to be controlled by IV adrenaline, corticosteroids or antihistamines.

Serum electrolyte disturbances: Hypomagnesemia, hypocalcemia, hyponatremia, hypokalemia and hypophosphatemia have been reported to occur in patients treated with cisplatin and are probably related to renal tubular damage. Hypomagnesaemia and hypocalcaemia may result in tetany. Generally, normal serum electrolyte levels are restored by administering supplemental electrolytes and discontinuing cisplatin. Inappropriate antidiuretic hormone syndrome has also been reported.

Neurotoxicity: Usually characterised by peripheral neuropathies and paresthesia in both upper and lower extremities. Peripheral neuropathy, while reversible, may take a year or more to recover. Loss of taste and seizures have also been reported. Neuropathies resulting from cisplatin treatment may occur after prolonged therapy; however, neurological symptoms have been reported to occur after a single dose. The neuropathy may progress after stopping treatment.

Hyperuricemia: Hyperuricemia occurring with cisplatin is more pronounced with doses greater than 50 mg/m^2. Allopurinol effectively reduces uric acid levels.

Other toxicities: Vascular toxicities coincident with the use of cisplatin in combination with other antineoplastic agents have been reported rarely. These events may include myocardial infarction, cerebrovascular accident, thrombotic microangiopathy (haemolytic uraemic syndrome) or cerebral arteritis. There are also reports of Raynaud's phenomenon occurring in patients treated with the combination of bleomycin, vinblastine with or without cisplatin. It has been suggested that hypomagnesaemia developing coincident with the use of cisplatin may be an added, although not essential factor, associated with this event. However the cause of this Raynaud's phenomenon is currently unknown.

Other toxicities reported to occur infrequently are cardiac abnormalities including tachycardia, postural hypotension and arrhythmia.

Local soft tissue toxicity has been reported rarely following extravasation of cisplatin. Infiltration of solutions of cisplatin may result in tissue cellulitis, fibrosis and necrosis.

Carboplatin - http://emc.medicines.org.uk (as per SmPC)

Safety profile
The main toxicities associated with carboplatin are:

Haematological toxicity: Myelosuppression is the dose-limiting toxicity of carboplatin. At maximum tolerated dosages of carboplatin administered as a single agent, thrombocytopenia, with nadir platelet counts of less than 50 x 10^9/L, occurs in about a quarter of the patients. The nadir usually occurs between days 14 and 21, with recovery within 35 days from the start of therapy. Leucopenia has also occurred in approximately 14% of patients but its recovery from the day of nadir (day 14-28) may be slower and usually occurs within 42 days from the start of therapy. Neutrogena with granulocyte counts below 1 x 10^9/L occurs in approximately one fifth of patients. Anaemia with haemoglobin values below 11g/dL has been observed in more than two-thirds of patients with normal base-line values.
Myelosuppression may be more severe and prolonged in patients with impaired renal function, extensive prior treatment, poor performance status and age above 65. Myelosuppression is also worsened by therapy combining carboplatin with other compounds that are myelosuppressive.
Myelosuppression is usually reversible and not cumulative when carboplatin is used as a single agent and at the recommended dosages and frequencies of administration.
Infectious complications have occasionally been reported. Haemorrhagic complications, usually minor, have also been reported.

Nephrotoxicity: Renal toxicity is usually not dose-limiting in patients receiving carboplatin, nor does it require preventive measures such as high volume fluid hydration or forced diuresis. Nevertheless, increasing blood urea or serum creatinine levels can occur. Renal function impairment, as defined by a decrease in the creatinine clearance below 60 ml/min, may also be observed. The incidence and severity of nephrotoxicity may increase in patients who have impaired kidney function before carboplatin treatment. It is not clear whether an appropriate hydration programme might overcome such an effect, but dosage reduction or discontinuation of therapy is required in the presence of severe alteration of renal function tests.

Decreases in serum electrolytes (sodium, magnesium, potassium and calcium) have been reported after treatment with carboplatin but have not been reported to be severe enough to cause the appearance of clinical signs or symptoms.
Cases of hyponatraemia have been reported. Haemolytic uraemic syndrome has been reported rarely.

Gastrointestinal toxicity: Nausea without vomiting occurs in about 15% of patients receiving carboplatin; vomiting has been reported in over half of the patients and about one-fifth of these suffer severe emesis. Nausea and vomiting usually disappear within 24 hours after treatment and are usually responsive to (and may be prevented by) anti-emetic medication. A fifth of patients experience no nausea or vomiting.

Cases of anorexia have been reported.

Allergic reactions: Infrequent allergic reactions to carboplatin have been reported, e.g., erythematous rash, fever with no apparent cause or pruritus. Rarely, anaphylaxis, angio-oedema and anaphylactoid reactions, including bronchospasm, urticaria and facial oedema have occurred.

Ototoxicity: Subclinical decrease in hearing acuity, consisting of high-frequency (4000-8000 Hz) hearing loss determined by audiogram, has been reported in 15% of the patients treated with carboplatin. However, only 1% of patients present with clinical symptoms, manifested in the majority of cases by tinnitus. In patients who have been previously treated with cisplatin and have developed hearing loss related to such treatment, the hearing impairment may persist or worsen.
At higher than recommended doses in combination with other ototoxic agents, clinically significant hearing loss has been reported to occur in paediatric patients when carboplatin solution was administered.

Neurotoxicity: The incidence of peripheral neuropathies after treatment with carboplatin is 4%. In the majority of the patients neurotoxicity is limited to paraesthesia and decreased deep tendon reflexes. The frequency and intensity of this side effect increases in elderly patients and those previously treated with cisplatin.

Paraesthesia present before commencing carboplatin therapy, particularly if related to prior cisplatin treatment, may persist or worsen during treatment with carboplatin.

Ocular toxicity: Transient visual disturbances, sometimes including transient sight loss, have been reported rarely with platinum therapy. This is usually associated with high dose therapy in renally impaired patients.

Other: Abnormalities of liver function tests (usually mild to moderate) have been reported with carboplatin in about one-third of the patients with normal baseline values. The alkaline phosphatase level is increased more frequently than SGOT, SGPT or total bilirubin. The majority of these abnormalities regress spontaneously during the course of treatment.

Infrequent events consisting of taste alteration, asthenia, alopecia, fever and chills without evidence of infection have occurred.

APPENDIX 8

EXPECTED ADVERSE EVENTS

	Thoracic Radiotherapy	Cisplatin	Etoposide
**Haematological**			
Anaemia	✓	✓	✓
Leucopenia	✓	✓	✓
Neutropenia			✓
Thrombocytopenia	✓	✓	✓
Febrile neutropenia		✓	✓
**Gastrointestinal**			
Oesophagitis	✓(unless grade 3 or 4)		
Abnormal liver transaminases		✓	✓
Anorexia	✓	✓	✓
Constipation			✓
Diarrhoea		✓	✓
Nausea	✓	✓	✓
Vomiting		✓	✓
**Renal**			
Hyperuricemia		✓	✓
Nephrotoxicity		✓	
**Allergic**			
Rash		✓	✓
Pruritus		✓	✓
Anaphylactic-like (flushing facial oedema, wheezing, tachycardia, hypotension, skin rash, fever)		✓	✓
**Respiratory**			
Bronchospasm		✓	✓
Pneumonitis	✓(unless grade 3 or 4)		
Pulmonary fibrosis	✓		
Dyspnoea	✓	✓	
**Neurotoxicity**			
Peripheral neuropathy		✓	✓
Loss of taste		✓	
Convulsions		✓	
**Skin**			
Desquamation	✓		
Erythema	✓		
**Other**			
Flu like symptoms	✓		
Fatigue	✓	✓	✓
Alopecia		✓	✓
Oral toxicity (soreness & erythema)		✓	✓
Ototoxicity-Tinnitus, hearing loss		✓	
Ocular toxicity		✓	
Vascular toxicity – cerebrovascular accident, myocardial infarction, cerebral arteritis		✓	
Cardiotoxicity – tachycardia,arrhythmia, postural hypotension		✓	
Hypotension			✓
Serum electrolyte disturbances		✓	

APPENDIX 9

COMPLIANCE WITH IR(ME)R 2000 REGULATIONS AND OTHER QA REQUIREMENTS.

All participating centres must comply with the requirements of the IR(ME)R 2000 Regulations and the Medical and Dental Guidance Notes, 2002.

Participating centres must have prior approval by the Local Research Ethics Committee. This will include general approval of the protocols for patient inclusion, patient consent and the justification of radiation exposures (for planning and treatment purposes).

Participating centres must operate a formal system of Quality Assurance in Radiotherapy (QART), which may include registration to an external standard (such as ISO9001:2000).

As part of the QART system, and in compliance with IR(ME)R 2000, participating centres must follow written protocols for radiotherapy treatment planning, prescription and delivery. In these protocols, there should be clear description of compliance with regard to the role of the employer, referrer and operator. The process for justification and authorisation of planning and treatment exposures must be clearly described (appendix 9).

Participating centres must participate in an external programme of dosimetry audit (such as that performed by IPEM). There must be no unresolved dosimetry discrepancies.

References:

IR(ME)R: Ionising Radiation (Medical Exposure) Regulations 2000 (SI 2000 No 1059), HMSO, London.

Medical and Dental Guidance Notes, 2002, Institute of Physics and Engineering in Medicine, York.

International Standard EN ISO 9001:2000. Quality Management Systems – Requirements. BSI, London, 2000.

APPENDIX 10

RADIOTHERAPY QUALITY ASSURANCE

Participating centres should be experienced in the selection of patients with limited stage small cell lung cancer (SCLC) for treatment with curative intent using high dose radiotherapy and chemotherapy, and the administration of such treatment.

The trial will be subject to a quality assurance programme. Centres will have to pass an initial assessment before patients may be randomised in the trial and there will be further assessments afterwards.

The initial assessment will consist of :

- Completion and return of a questionnaire detailing the facilities available to the centre.
- Return of 2 radiotherapy treatment plans for a patient with limited stage SCLC, previously treated in the centre with radical intent, who satisfied the eligibility criteria for the CONVERT trial, and has been replanned according to the CONVERT protocol for each treatment arm; 66Gy in 33 daily fractions and 45Gy in 30 fractions treated twice per day.

If there are several participating clinicians in each centre then a centre may begin to randomise patients after the Principal Investigator has passed the initial QA assessment but all clinicians will be required to submit plans for patients previously treated by themselves before they may independently randomise patients.

The Trial Office will subsequently send each participating centre clinical histories with results of investigations; bronchoscopy, respiratory function tests, CT and PET scans, along with diagnostic and planning CT scans on a compact disc.
In one exercise the participating clinicians will outline the Gross Tumour Volume (GTV). The GTV will be expanded to give the Clinical Target Volume (CTV) and the Planning Target Volume (PTV).
In another exercise the GTV will already be outlined and the dosimetrist will perform expansions to give CTV and PTV (part of a phantom exercise).

Further exercises will include a right sided lung cancer with ipsilateral mediastinal lymphadenopathy and a left sided lung cancer with contralateral mediastinal lymphadenopathy. Clinicians will outline the GTV, expansions will then be performed to give the PTV and dosimetrists will plan for treatment.

During the trial plans will be randomly requested from each clinician in each centre as part of continuing quality assurance.

Participating centres will have to agree to address uncertainties revealed by the QA programme.

The quality assurance program is based at Mount Vernon Hospital
Responsible clinician Dr Ethan Lyn (ethanlyn@mac.com)

APPENDIX 11

SITE PERSONNEL FORM

Full contact details of all trial personnel

Hospital: ______________________________ **Principal Investigator:** ______________________

Address: ______________________________ **Main contact person** ______________________
(e.g. data queries, general correspondence)

____

____

Complete this form for all trial personnel and return with a brief CV for each
Notify the Christie Hospital CTU of any contact or trial personnel changes
Use additional sheets if necessary

Name ____
(title, first name, surname, position)
Department ____
Phone ____________________
Fax ____________________
E-mail ____
Address ____
(if different from above) ____

Name ____
(title, first name, surname, position)
Department ____
Phone ____________________
Fax ____________________
E-mail ____
Address ____
(if different from above) ____

Name ____
(title, first name, surname, position)
Department ____
Phone ____________________
Fax ____________________
E-mail ____
Address ____
(if different from above) ____

CONVERT: Signature list and delegation of responsibilities

APPENDIX 12

Institution: ____

This form must be completed by all personnel managing patients and those responsible for completing CRFs (e.g oncologists, surgeons, pathologists and research nurses/data managers). <u>Only staff who are included on this form will be authorised to sign CRFs.</u>

Use the codes below to complete the Study Responsibilities column. Enter the number(s) that correspond to the responsibilities of each individual. For responsibilities that are not listed as codes, please specify.

1) Medical care of patients
2) Adverse event reporting
3) Ethics/regulatory approval
4) Registration/Randomisation
5) Informed consent
6) CRF completion
7) Response to data queries
8) Trial master file maintenance
9) Drug accountability
10) Pathology specimen processing
11) Pharmacy
12) Laboratory

**Name**	**Job title**	**Sample signature**	**Sample initials**	**Date commenced work on trial**	**Date ended work on trial**	**Study Responsibilities** (see note and codes above)	**Authorisation of principal investigator**

Please notify us of any changes to trial personnel by updating this form and sending it to the Christie Hospital CTU.

APPENDIX 13

RECORD OF CHANGES MADE TO PROTOCOL

This version of the protocol supercedes version 2, dated 13FEB2008.

The following changes have been implemented in this version of the protocol:

Page 4: trial manager and research nurse contact details updated

Page 15: Details of pulmonary function tests required at pre-randomisation evaluation updated:

*"Pulmonary function tests [~~FEV1 and transfer factor (KCO or DLCO/VA)~~ **FVC, FEV1, KCO and DLCO/VA)**]*

Pages 15 & 30: Removal of CXR from assessments required at screening. Previously the table of assessments listed a CXR at both screening & cycle 1. It is not necessary to perform a chest X-ray at both timepoints, therefore the CXR at screening has been removed.

Page 16: Addition to eligibility criteria:

"Adequate liver function: ALT & AST <= 2.5 x ULN"

Page 30: Correction of timepoints in summary of protocol assessments for research tumour sample- a tumour sample is NOT required at the end of treatment, only at baseline and on relapse (if available).

Page 30: Clarification of timepoints in summary of protocol assessments for research blood samples- at cycle 1 these are to be taken on days 1 & 22, as per information included in the patient information sheet.

Pages 26-27: Definitions and guidance relating to what constitutes a serious adverse event have been updated. Grade 4 and/or life threatening expected adverse events listed in appendix 8 are no longer exempt from expedited reporting. Deaths should be considered SAEs if they occur with 30 days of last dose of chemotherapy or radiotherapy, regardless of causality. Events which are life threatening/ result in hospitalization or disability should be reported as SAEs only if considered related to study treatment. Hospitalisations for palliative care, chemotherapy, radiotherapy, procedures or diagnostic procedures do not need to be reported as SAEs. Ordering/ format of other sections of text previously present in this section has been updated slightly for clarity.

Pages 29 & 30: Timing of follow-up CT scan of chest & abdomen: clarification in table of assessments that 6 month follow-up scan refers to 6 months after randomisation date. Addition of further scan at 12 months after randomisation date.

Page 49: In selected NCI Common Terminology Criteria for Adverse Events (v3.0) the category 'Renal-other' has been replaced by 'Creatinine'. All text copied verbatim from CTCAE v3.0.

Page 50: Addition of further category to MRC dyspnoea score that had been omitted in error:

"5- Dyspnoea at rest"

Page 61: Removal of radiotherapy quality assurance questionnaire from appendix 10. The information to be collected in this questionnaire has now been expanded by the QA team. The QA team will provide this questionnaire to participating sites as a document separate from the protocol.

CONVERT trial protocol: Version 2, dated 13FEB2008 superseded Version 1, dated 01NOV2007. RECORD OF CHANGES MADE TO PROTOCOL

The following changes have been implemented in this version of the protocol:

Page 1: addition of logos for collaborating groups

Page 4: addition of contact details for collaborating groups

Page 4: randomisation details updated- randomisation will be by minimization, not by permuted blocks within 4 strata:
"Patients will be randomly allocated to treatment, ~~using permuted blocks within 4 strata~~ ***a minimization procedure."***

Page 7: addition of statement clarifying that recruitment target of 532 patients is a total for all countries, including any contribution of patients from collaborating groups.

Page 14: clarification of eligible clinicians: *"all centres must … have discussed suitable patients in a Multi-Disciplinary Meeting, including a ~~lung cancer specialist~~* ***clinician with a special interest in lung cancer"***

Page 16: randomisation details updated- randomisation will be by mimimization, not by permuted blocks within 4 strata:

"Randomisation will be administered centrally by the trials office at the Christie Hospital. This is a multi-centre randomized phase III trial. Patients will be randomized on a 1:1 basis to one of two treatment arms. ***The allocation method will be ~~permuted blocks within 4 strata~~ minimization with a random element.*** *Randomisation will be implemented in a bespoke computer application at the randomisation centre. The factors controlled for in the allocation will be:*

Institution
Planned number of cycles (4 or 6)
Performance status (0/1 or 2) {PS2 equivalent to KP 50, 60}

The arm that would result in less imbalance will be allocated with probability 0.75. There will be a 'burn-in' period of 20 cases in which allocation will be completely random."

Page 18: Statement added about benefits of stopping smoking*: "Prior to commencing treatment patients who are current smokers should be advised of the benefits of stopping smoking, and should be offered any available support to quit. Stopping smoking is associated with a reduction in the risk of radiation induced side effects and in some patients also results in improved survival."*

Page 18: correction of timing of radiotherapy versus chemotherapy cycles in OD arm: *"In OD radiotherapy arm, the third* ***and fourth*** *cycle***s** *of PE ~~is~~* ***are*** *also given concurrently and the ~~fourth to~~* ***fifth and sixth*** *cycles (if applicable) are given after completion of the thoracic radiotherapy."*

Page 22: missing units added to statement about contouring of spinal cord: *"The spinal cord will be contoured based on the bony limits of the spinal canal. The spinal cord should be contoured starting at least 10 cm above the superior extent of the PTV and continuing on every CT slice to at least 10cm below the inferior extent of the PTV."*

Page 22: section vii, clarification of lung contouring- collapsed lung should not be contoured: *"Both the right and left lungs should be contoured as one structure. Contouring should be carried out using pulmonary windows. All inflated* ~~***and collapsed***~~ *lung should be contoured."*

Page 25: clarification of procedure for patients not meeting the radiotherapy planning requirements: *"If the V20 lung,* ~~***maximum length of oesophagus receiving 40 Gy, V35 oesophagus,***~~ *dose to the spinal cord* ***and heart*** *do not meet the protocol requirements, the treatment decision will be left to the local investigator, as some adjustments may be necessary regarding the radiotherapy treatments* ~~***(eg total dose prescribed, margins…).***~~ ***We strongly recommend that the total dose of radiotherapy is dropped to meet the normal tissue constraints."***

Page 33: clarification of sample size calculation: *"Using Freedman's sample size calculation based on a 2-arm trial, with a 5% significance level, 2-sided test, 80% power, and hazard ratio of 0.70, a 12% overall survival benefit at 2 years from 44% with the control arm to 56% in the experimental arm, requires a total of 506 patients.* ***The number of deaths required is 247."***

Page 36: stratification section deleted- as per changes listed above to randomisation section (page 16) patients will not be allocated to strata, but will be allocated using minimization.

Page 49: addition of cough and dyspnea to the list of 'Selected NCI Common Toxicity Criteria for Adverse Events v3.0'. All text taken from the full CTCAE at http://ctep.cancer.gov

Page 64: Reformatting of signature list & delegation log.

CONVERT trial protocol: Version 3, dated 10JUN2008 superseded Version 2, dated 13FEB2008
RECORD OF CHANGES MADE TO PROTOCOL

The following changes have been implemented in this version of the protocol:

Page 4: trial manager and research nurse contact details updated

Page 15: Details of pulmonary function tests required at pre-randomisation evaluation updated:
*"Pulmonary function tests ~~[FEV1 and transfer factor (KCO or DLCO/VA)~~ **FVC, FEV1, KCO and DLCO/VA)]***

Pages 15 & 30: Removal of CXR from assessments required at screening. Previously the table of assessments listed a CXR at both screening & cycle 1. It is not necessary to perform a chest X-ray at both timepoints, therefore the CXR at screening has been removed.

Page 16: Addition to eligibility criteria:
"Adequate liver function: ALT & AST <= 2.5 x ULN"

Page 30: Correction of timepoints in summary of protocol assessments for research tumour sample - a tumour sample is NOT required at the end of treatment, only at baseline and on relapse (if available).

Page 30: Clarification of timepoints in summary of protocol assessments for research blood samples - at cycle 1 these are to be taken on days 1 & 22, as per information included in the patient information sheet.

Pages 26-27: Definitions and guidance relating to what constitutes a serious adverse event have been updated. Grade 4 and/or life threatening expected adverse events listed in appendix 8 are no longer exempt from expedited reporting. Deaths should be considered SAEs if they occur with 30 days of last dose of chemotherapy or radiotherapy, regardless of causality. Events which are life threatening/ result in hospitalization or disability should be reported as SAEs only if considered related to study treatment. Hospitalisations for palliative care, chemotherapy, radiotherapy, procedures or diagnostic procedures do not need to be reported as SAEs. Ordering/ format of other sections of text previously present in this section has been updated slightly for clarity.

Pages 29 & 30: Timing of follow-up CT scan of chest & abdomen: clarification in table of assessments that 6 month follow-up scan refers to 6 months after randomisation date. Addition of further scan at 12 months after randomisation date.

Page 49: In selected NCI Common Terminology Criteria for Adverse Events (v3.0) the category 'Renal-other' has been replaced by 'Creatinine'. All text copied verbatim from CTCAE v3.0.

Page 50: Addition of further category to MRC dyspnoea score that had been omitted in error:
"5- Dyspnoea at rest"

Page 61: Removal of radiotherapy quality assurance questionnaire from appendix 10. The information to be collected in this questionnaire has now been expanded by the QA team. The QA team will provide this questionnaire to participating sites as a document separate from the protocol.

CONVERT Statistical Analysis Plan

The analysis will be based on intention-to-treat principles (ITT). Patient data will be grouped by treatment arm according to the treatment assignments made via the MAHSC-CTU randomisation line. The primary data analysis is planned for January 2015.

The stratification factors include centre, PS (0–1 vs 2) and biochemical factors (eg, LDH, sodium, alkaline phosphatase). Comparison of data by centre will be scrutinised to identify any data inconsistencies, it will also be used to identify those centres planning to give either four or six cycles of chemotherapy. Analysis will be carried out to identify any differences between the two schedules. Other factors have been identified in previous studies to be prognostic factors and will be used to calculate the Manchester score, which gives three groupings of good, intermediate and poor prognosis; these scores will be used to compare OS and response rate.

The only planned interim analyses have been performed for the Independent Data Monitoring Committee (IDMC). Reports have been submitted to the IDMC on an annual basis starting 12 months after the first patient was randomised.

The following are the qualifications for analysis of time-to-event efficacy parameters:

‣ All randomised patients will be included in the analysis of overall survival (OS) and local progression free survival (ITT).

OS is the time between date of randomisation and date of death of any cause. Survivors will be censored on the last date known to be alive. Local progression-free survival (local control) will be calculated from the date of randomisation to the date of first clinical evidence of progressive disease at the primary site, or death. Kaplan-Meier curves will be drawn for each treatment group. Overall survival and local progression-free survival will be compared using the Mantel-Cox version of the log rank test.

‣ All randomised patients treated with at least one study dose of cisplatin and etoposide will be included in the comparison of proportions of grade 3 and 4 toxicities.

Toxicity will be assessed according to NCI Common Terminology Criteria for Adverse Events V.3.0. The proportion of patients experiencing a grade of 3 or above acute toxicity, including acute and/or late radiation morbidity, will be compared between the treatment groups using $\chi 2$ and Fisher Exact tests. Acute toxicity will be defined as toxicities occurring from commencement to 3 months after completion of treatment; late toxicity will be defined as toxicities occurring between 3 months and 2 years after completion of treatment. All patients treated with at least one dose of cisplatin and etoposide will be evaluated for tumour response and included in the analysis of tumour response rates.

Response will be assessed according to Response Evaluation Criteria In Solid Tumours (RECIST) criteria. The proportion of patients in each treatment group whose best response (up to approximately 28 days post cycle 4 or, if stopped prior to cycle 4, approximately 28 days after last chemotherapy cycle given) from randomisation is complete or partial will be compared using $\chi 2$ and Fisher Exact tests.

Safety analyses will be performed for all randomised patients treated with at least one dose of chemotherapy. Adverse effects will be summarised and compared between the two arms. The relative chemotherapy and RT dose intensity (RDI) will be summarised by calculating the median, SD, IQR and range for patients in each randomised treatment group. The RDI will be compared between the treatment groups by using the Wilcoxon Rank Sum test.

A detailed description of patient disposition will include a summary of the following:

- All patients entered and enrolled: overall, by treatment arm and by country
- Reasons for patients entered but not enrolled
- All enrolled patients treated with study drug, by treatment arm
- Reasons patients enrolled but not treated with study drug
- Reasons patients discontinued study drug treatment
- All important protocol violations.

CONVERT Statistical Analysis Plan

The analysis will be based on intention-to-treat principles (ITT). Patient data will be grouped by treatment arm according to the treatment assignments made via the MAHSC-CTU randomisation line. The primary data analysis is planned for January 2015.

The stratification factors include centre, PS (0–1 vs 2) and biochemical factors (eg, LDH, sodium, alkaline phosphatase). Comparison of data by centre will be scrutinised to identify any data inconsistencies, it will also be used to identify those centres planning to give either four or six cycles of chemotherapy. Analysis will be carried out to identify any differences between the two schedules. Other factors have been identified in previous studies to be prognostic factors and will be used to calculate the Manchester score, which gives three groupings of good, intermediate and poor prognosis; these scores will be used to compare OS and response rate.

The only planned interim analyses have been performed for the Independent Data Monitoring Committee (IDMC). Reports have been submitted to the IDMC on an annual basis starting 12 months after the first patient was randomised.

The following are the qualifications for analysis of time-to-event efficacy parameters:
‣ All randomised patients will be included in the analysis of overall survival (OS) and local progression free survival (ITT).
OS is the time between date of randomisation and date of death of any cause. Survivors will be censored on the last date known to be alive. Local progression-free survival (local control) will be calculated from the date of randomisation to the date of first clinical evidence of progressive disease at the primary site, or death. Kaplan-Meier curves will be drawn for each treatment group. Overall survival and local progression-free survival will be compared using the Mantel-Cox version of the log rank test.

‣ All randomised patients treated with at least one study dose of cisplatin and etoposide will be included in the comparison of proportions of grade 3 and 4 toxicities.
Toxicity will be assessed according to NCI Common Terminology Criteria for Adverse Events V.3.0. The proportion of patients experiencing a grade of 3 or above acute toxicity, including acute and/or late radiation morbidity, will be compared between the treatment groups using $\chi 2$ and Fisher Exact tests. Acute toxicity will be defined as toxicities occurring from commencement to 3 months after completion of treatment; late toxicity will be defined as toxicities occurring between 3 months and 2 years after completion of treatment. All patients treated with at least one dose of cisplatin and etoposide will be evaluated for tumour response and included in the analysis of tumour response rates.

Response will be assessed according to Response Evaluation Criteria In Solid Tumours (RECIST) criteria. The proportion of patients in each treatment group whose best response (up to approximately 28 days post cycle 4 or, if stopped prior to cycle 4, approximately 28 days after last chemotherapy cycle given) from randomisation is complete or partial will be compared using $\chi 2$ and Fisher Exact tests.

Safety analyses will be performed for all randomised patients treated with at least one dose of chemotherapy. Adverse effects will be summarised and compared between the two arms. The relative chemotherapy and RT dose intensity (RDI) will be summarised by calculating the median, SD, IQR and range for patients in each randomised treatment group. The RDI will be compared between the treatment groups by using the Wilcoxon Rank Sum test.

A detailed description of patient disposition will include a summary of the following:

- All patients entered and enrolled: overall, by treatment arm and by country
- Reasons for patients entered but not enrolled
- All enrolled patients treated with study drug, by treatment arm
- Reasons patients enrolled but not treated with study drug
- Reasons patients discontinued study drug treatment
- All important protocol violations.
