# Supplementary material for: Association of Chemoradiotherapy With Outcomes Among Patients With Stage I to II vs Stage III Small Cell Lung Cancer: Secondary Analysis of a Randomized Clinical Trial
Source: JAMA Oncol. 2018 Dec 6;5(3):e185335. doi: 10.1001/jamaoncol.2018.5335 (PMC6439849; doi:10.1001/jamaoncol.2018.5335)
Supplement: Supplement 2. — eTable 1. Tumor and Nodal Staging in Stage I-II Patients eTable 2. Baseline and Treatment Characteristics in Stage I-II and Stage III According to Trial Arm eTable 3. Type of Tumor Progression in Stage I-II and Stage III eTable 4. Site of Tumour Progression in Stage I-II and Stage III eTable 5. Comparison of Received Radiotherapy Dose According to Treatment Arm in Stage I-II and Stage III eTable 6. Comparison of Acute Toxicity in Stage I-II and Stage III eTable 7. Comparison of Late Toxicity in Stage I-II and Stage III eFigure. Overall Survival According to Trial Arm and Staging [18F]FDG PET [file jamaoncol-5-e185335-s002.pdf]

Supplementary Online Content

Salem A, Mistry H, Hatton M, et al. Association of chemoradiotherapy with outcomes among patients with stage I to II vs stage III small cell lung cancer: secondary analysis of a randomized clinical trial. *JAMA Oncol.* Published online December 6, 2018. doi:10.1001/jamaoncol.2018.5335

eTable 1. Tumor and Nodal Staging in Stage I-II Patients

eTable 2. Baseline and Treatment Characteristics in Stage I-II and Stage III According to Trial Arm

eTable 3. Type of Tumor Progression in Stage I-II and Stage III

eTable 4. Site of Tumour Progression in Stage I-II and Stage III

eTable 5. Comparison of Received Radiotherapy Dose According to Treatment Arm in Stage I-II and Stage III

eTable 6. Comparison of Acute Toxicity in Stage I-II and Stage III

eTable 7. Comparison of Late Toxicity in Stage I-II and Stage III

eFigure. Overall Survival According to Trial Arm and Staging [18F]FDG PET

This supplementary material has been provided by the authors to give readers additional information about their work.

© 2018 American Medical Association. All rights reserved.

eTable 1. Tumor and Nodal Staging in Stage I-II Patients

**Tumour and nodal stage**	**Number of patients (%)[a]**
T1a/bN0	4 (5)
T2bN0	29 (34)
T3N0	15 (17)
T1a/bN1	16 (19)
T2a/bN1	22 (26)

[a] Percentage does not add to 100% due to approximation

© 2018 American Medical Association. All rights reserved.

eTable 2. Baseline and Treatment Characteristics in Stage I-II and Stage III According to Trial Arm

	Category	Stage I-II (n=86)		Stage III (n=423)	
		Once-daily (n=51)	Twice-daily (n=35)	Once-daily (n=204)	Twice-daily (n=219)
Median age (range)	n/a	61<br>(39-77)	62<br>(29-76)	64<br>(34-81)	62<br>(36-81)
Sex (%)	Male<br>Female	32 (63)<br>19 (37)	19 (54)<br>16 (46)	105 (51)<br>99 (49)	121 (55)<br>98 (45)
Smoking history (%)	Never<br>Ex-smoker<br>Current smoker	1 (2)<br>25 (49)<br>25 (49)	1 (3)[a]<br>24 (69)[a]<br>10 (29)[a]	2 (1)<br>127 (62)<br>75 (37)	2 (1)<br>142 (65)<br>75 (34)
ECOG PS (%)	0<br>1<br>2	28 (55)<br>21 (41)<br>2 (4)	21 (60)<br>13 (37)<br>1 (3)	85 (42)<br>113 (55)<br>6 (3)	99 (45)<br>113 (52)<br>7 (3)
MRC dyspnoea score (%)	0<br>1-2<br>3-4<br>Not assessed	25 (49)<br>17 (33)<br>6 (12)<br>3 (6)	13 (37)<br>18 (51)<br>2 (6)<br>2 (6)	61 (30)<br>106 (52)<br>23 (11)<br>14 (7)	78 (36)[a]<br>109 (50)[a]<br>17 (8)[a]<br>15 (7)[a]
Staging [^{18}F]FDG PET (%)	No<br>Yes<br>Not known	17 (33)<br>34 (67)<br>0 (0)	11 (31)<br>24 (69)<br>0 (0)	93 (46)<br>111 (54)<br>0 (0)	96 (44)<br>122 (56)<br>1 (<1)
Planned number of chemotherapy cycles (%)	4<br>6	36 (71)<br>15 (29)	24 (69)<br>11 (31)	135 (66)<br>69 (34)	152 (69)<br>67 (31)
Median GTV<br>(cm^3, range)	n/a	35·2<br>(5·8-593)	45·9<br>(2·2-389·2)	95·9<br>(0·5-447·8)	88·85<br>(1·6-513·4)
Radiotherapy (%)	Concurrent<br>Sequential<br>No radiotherapy	46 (90)<br>1 (2)<br>4 (8)	33 (94)<br>1 (3)<br>1 (3)	180 (88)<br>4 (2)<br>20 (10)	202 (92)[a]<br>3 (1)[a]<br>14 (6)[a]
IMRT (%)	Yes<br>No	9 (18)<br>42 (82)	5 (14)<br>30 (86)	33 (16)<br>171 (84)	34 (16)<br>185 (84)
PCI (%)	Yes<br>No	46 (90)<br>3 (6)	32 (91)<br>3 (9)	162 (79)[a]<br>33 (16)[a]	184 (84)<br>26 (12)

© 2018 American Medical Association. All rights reserved.

	Missing data	2 (4)	0 (0)	9 (4)[a]	9 (4)
	**Category**	**Stage I-II (n=86)**		**Stage III (n=423)**	
		**Once-daily (n=51)**	**Twice-daily (n=35)**	**Once-daily (n=204)**	**Twice-daily (n=219)**
Median minimum PTV % dose (D_{min} %) (range)	n/a	89 (36-99)	91 (32-100)	87 (0-100)	88 (0-100)

Abbreviations: IMRT, intensity modulated radiation therapy, MRC, Medical Research Council

[a] Percentages do not add to 100% due to approximation

© 2018 American Medical Association. All rights reserved.

eTable 3. Type of Tumor Progression in Stage I-II and Stage III

Type of progression	Stage I-II (n=86)[a]	Stage III (n=423)[a]
Loco-regional (%)	12 (14)	73 (17)
Distant (%)	23 (27)	170 (40)
Death (%)	12 (14)	64 (15)

[a] At the time of analysis, 35 (41%) of 86 stage I-II and 243 (57%) of 423 stage III patients had disease progression

© 2018 American Medical Association. All rights reserved.

eTable 4. Site of Tumor Progression in Stage I-II and Stage III

Site of tumour progression	Stage I-II[a]	Stage III[a]
Local (%)	15 (21)	98 (20)
Nodal (%)	11 (15)	92 (19)
Distant (%)	46 (64)	293 (61)
Contralateral lung (%)	*3 (4)*	*15 (3)*
Liver (%)	*14 (19)*	*67 (14)*
Bone (%)	*7 (10)*	*40 (8)*
Brain (%)	*6 (8)*	*77 (16)*
Others (%)	*16 (22)*	*94 (19)*

a In patients with more than one site of tumour progression recorded on the case report forms, these were registered separately in each category (even if more than one site of local/ regional and distant progression). For this reason, the number of relapse events registered in stage III patients is more than the number of patients.

© 2018 American Medical Association. All rights reserved.

eTable 5. Comparison of Received Radiotherapy Dose According to Treatment Arm in Stage I-II and Stage III

	**Stage I-II (once-daily/ twice-daily)**	**Stage III (once-daily/ twice-daily)**	**Chi-sq (*P* value)**
**Radiotherapy fractions received (once-daily/ twice-daily)**			
<33/<30	12 (4/8)	74 (44/30)	·60
33/30	69 (43/26)	314 (139/175)	
>33/>30	0 (0/0)	1 (1/0)	
**Radiotherapy dose received (once-daily/ twice-daily)**			
<28 Gy (%)	0 (0) (0/ 0)	0 (0) (0/ 0)	·11
<60/ <44 Gy (%)	1 (1) (1/ 0)	27 (6) (25/ 2)	
≥60/ ≥44 Gy (%)	80 (93) (46/ 34)	362 (86) (159/ 203)	
No radiotherapy (%)	5 (6) (4/ 1)	34 (8) (20/ 14)	

© 2018 American Medical Association. All rights reserved.

eTable 6. Comparison of Acute Toxicity in Stage I-II and Stage III

**Toxicity**	**Grade**	**Stage I-II**	**Stage III**	**Chi-sq (*P* value)**
Oesophagitis (%)	0	31 (38)	70 (18)	**<·001**
	1	22 (28)	75 (19)	
	2	18 (23)	162 (42)	
	3	9 (11)	81 (21)	
	4	0 (0)	1 (<1)	
Pneumonitis (%)	0	70 (88)[a]	355 (92)[a]	·13
	1	7 (9)[a]	21 (5)[a]	
	2	3 (4)[a]	4 (1)[a]	
	3	0 (0)[a]	5 (1)[a]	
Dermatitis (%)	0	54 (68)	284 (74)	·08
	1	24 (30)	76 (20)	
	2	2 (2)	23 (6)	
Neutropenia (%)	0	13 (15)	56 (14)	·07
	1	4 (5)	33 (8)	
	2	12 (14)	32 (8)	
	3	14 (17)	116 (28)	
	4	41 (49)	175 (42)	

[a] Percentages do not add to 100% due to approximation

© 2018 American Medical Association. All rights reserved.

eTable 7. Comparison of Late Toxicity in Stage I-II and Stage III

Toxicity	Grade	Stage I-II	Stage III	Chi-sq (*P* value)
Oesophagitis (%)	0	72 (91)	312 (83)[a]	·22
	1	6 (8)	35 (9)[a]	
	2	1 (1)	23 (6)[a]	
	3	0 (0)	4 (1)[a]	
Pneumonitis (%)	0	52 (65)	254 (68)[a]	·57
	1	17 (21)	71 (19)[a]	
	2	7 (9)	39 (10)[a]	
	3	3 (4)	7 (2)[a]	
	4	1 (1)	1 (<1)[a]	
Dermatitis (%)	0	75 (94)	350 (93)	·72
	1	5 (6)	22 (6)	
	2	0 (0)	3 (1)	
Myelitis (%)	0	77 (97)	367 (98)	1·00
	1	2 (3)	7 (2)	
Pulmonary Fibrosis (%)	0	41 (51)	188 (51)	·85
	1	30 (38)	150 (41)	
	2	8 (10)	27 (7)	
	3	1 (1)	4 (1)	
Oesophageal Fistula (%)	0	80 (100)	375 (100)	n/a
	1	0 (0)	0 (0)	
Oesophageal Stricture (%)	0	78 (98)	364 (97)	·92
	1	1 (1)	7 (2)	
	2	1 (1)	3 (1)	
	3	0 (0)	1 (<1)	

[a] Percentages do not add to 100% due to approximation

© 2018 American Medical Association. All rights reserved.
