# Supplementary material for: Association of Chemoradiotherapy With Outcomes Among Patients With Stage I to II vs Stage III Small Cell Lung Cancer: Secondary Analysis of a Randomized Clinical Trial
Source: JAMA Oncol. 2018 Dec 6;5(3):e185335. doi: 10.1001/jamaoncol.2018.5335 (PMC6439849; doi:10.1001/jamaoncol.2018.5335)
Supplement: Supplement 3. — Data Sharing Statement [file jamaoncol-5-e185335-s003.pdf]

Data Sharing Statement

Salem. Association of Chemoradiotherapy With Outcomes Among Patients With Stage I to II vs Stage III Small Cell Lung Cancer. *JAMA Oncol*. Published December 06, 2018. 10.1001/jamaoncol.2018.5335

Data
Data available: No
